# Associations of Serum Lipid Traits With Fracture and Osteoporosis: A Prospective Cohort Study From the UK Biobank

**DOI:** 10.1002/jcsm.13611

**Published:** 2024-10-29

**Authors:** Xi Xiong, David T. W. Lui, Chengsheng Ju, Ziyi Zhou, Chao Xu, Paul Welsh, Naveed Sattar, Carlos Celis‐Morales, Jill P. Pell, Ian C. K. Wong, Carlos K. H. Wong, Frederick K. Ho

**Affiliations:** ^1^ Centre for Safe Medication Practice and Research, Department of Pharmacology and Pharmacy, Li Ka Shing Faculty of Medicine The University of Hong Kong Hong Kong SAR China; ^2^ School of Health and Wellbeing University of Glasgow Glasgow UK; ^3^ Li Ka Shing Faculty of Medicine, Department of Medicine, School of Clinical Medicine The University of Hong Kong Hong Kong SAR China; ^4^ Research Department of Practice and Policy, School of Pharmacy University College London London UK; ^5^ Institute of Cardiovascular Science University College London London UK; ^6^ School of Cardiovascular and Metabolic Health University of Glasgow Glasgow UK; ^7^ Centro de Investigación en Medicina de Altura (CEIMA) Universidad Arturo Prat Iquique Chile; ^8^ Laboratory of Data Discovery for Health (D^2^4H) Hong Kong Science and Technology Park Hong Kong SAR China; ^9^ Aston Pharmacy School Aston University Birmingham UK; ^10^ Advanced Data Analytics for Medical Science (ADAMS) Limited Hong Kong China; ^11^ Department of Family Medicine and Primary Care, School of Clinical Medicine, Li Ka Shing Faculty of Medicine The University of Hong Kong Hong Kong SAR China; ^12^ Faculty of Epidemiology and Population Health, Department of Infectious Disease Epidemiology & Dynamics London School of Hygiene and Tropical Medicine London UK

**Keywords:** apolipoprotein A, apolipoprotein B, fracture, high‐density lipoprotein cholesterol, low‐density lipoprotein cholesterol, triglycerides

## Abstract

**Background:**

Previous studies reveal inconsistent associations between serum lipid traits and the risks of fractures and osteoporosis in the general population.

**Methods:**

This prospective cohort study analysed data from 414 302 UK Biobank participants (223 060 women and 191 242 men, aged 37–73 years) with serum lipid measurements: apolipoprotein A (Apo A), apolipoprotein B (Apo B), total cholesterol (TC), high‐density lipoprotein cholesterol (HDL‐C), low‐density lipoprotein cholesterol (LDL‐C), triglycerides (TG) and lipoprotein A (Lp(a)). Multivariable Cox proportional hazard models with penalized cubic splines were used to explore potential nonlinear associations of each lipid trait with the risks of fractures and osteoporosis. Subgroup analyses by age, sex, BMI categories and pre‐existing cardiovascular disease were conducted. Mediation analyses using the g‐formula were performed to quantify to which extent bone mineral density (BMD) may mediate the association between serum lipids and fracture risk.

**Results:**

Over a median follow‐up period of 13.8 years, 25 918 (6.8%) of the 383 530 participants without prior fracture had incident fracture cases, and 7591 (4.1%) of the 184 919 participants with primary care data and without baseline osteoporosis were diagnosed with osteoporosis. TG had nonlinear associations with fractures and osteoporosis, whereas Apo B, TC and LDL‐C had linear associations. There were also nonlinear associations of Apo A and HDL‐C with fractures. Individuals in the highest quintiles for Apo A (fracture: HR 1.15 [95% CI 1.10, 1.21]; osteoporosis: HR 1.13 [1.02, 1.25]) and HDL‐C (fracture: HR 1.27 [1.20, 1.34]; osteoporosis: HR 1.31 [1.18, 1.46]) were associated with higher risks of fractures and osteoporosis. Conversely, those in the highest quintile for Apo B (fracture: HR 0.85 [0.81, 0.89]; osteoporosis: HR 0.86 [0.79, 0.94]), LDL‐C (fracture: HR 0.89 [0.85, 0.93]; osteoporosis: HR 0.91 [0.83, 1.00]) and TG (fracture: HR 0.78 [0.74, 0.82]; osteoporosis: HR 0.75 [0.68, 0.82]) were associated with lower risks. The associations of Apo A (ratio of HR [RHR] 1.05 [1.02, 1.09]) and HDL‐C (RHR 1.06 [1.03, 1.09]) with fracture risk were more pronounced in men compared to women. Except for TG and Lp(a), the associations between serum lipids and fractures appear to be partially mediated through BMD (mediation proportions: 5.30% to 40.30%), assuming causality.

**Conclusions:**

Our study reveals a complex interplay between different lipid markers and skeletal health, potentially partially mediated through BMD. Routine lipid profile assessments, including HDL‐C and Apo A among other lipid traits, may be integrated into the strategies for fracture risk stratification.

## 1 Introduction

Osteoporosis, a prevalent metabolic disorder in the aging population, is characterized by diminished bone mineral density and increased susceptibility to fractures, resulting in substantial burdens on individuals and healthcare systems [1]. Concurrently, clinical studies have revealed a noteworthy interconnection between fractures, osteoporosis and cardiovascular diseases (CVDs) [2, 3]. This association is thought to be attributed to shared risk factors and/or common pathophysiological pathways underlying both conditions [4]. Notably, atherosclerosis and vascular calcification have emerged as key links between fractures, osteoporosis, and CVDs [4].

While the influence of serum lipids on atherosclerotic plaque formation and the development of cardiovascular diseases is well‐established, the precise relationship between serum lipids and fractures or osteoporosis remains a subject of contention. The potential roles of lipids in the pathogenesis of fractures and osteoporosis are rather complicated. The deterioration of trabecular microarchitecture due to impaired nutrient delivery and bone remodelling has been reported in patients with diabetes with vascular complications [5]. Dyslipidaemia and chronic hyperglycaemia contribute to oxidative stress and inflammation, leading to the formation of advanced glycation end products that stiffen bone collagen and increase fracture risk [5]. Additionally, dyslipidaemia promotes bone marrow adiposity, further weakening bone structure [5, 6]. It may also directly influence bone mineral density by affecting osteoblastic [7] or osteoclastic [8] activities, altering important hormones like parathyroid hormone and disturbing the metabolism of crucial nutrients, namely calcium and vitamin D [4].

Many clinical studies have endeavoured to unravel the relationship between blood lipids and the risk of fractures or osteoporosis, but the findings have been inconclusive and conflicting [9, 10, 11, 12, 13, 14, 15, 16, 17]. A recent publication from the ASPREE trial demonstrated that higher concentrations of high‐density lipoprotein cholesterol (HDL‐C) are associated with increased fracture risk in older people without cardiovascular disease [9]. However, the potential pathophysiological explanations for this association regarding changes in HDL‐C and bone mineral density (BMD) are inconsistent in Mendelian randomization studies [16, 18]. Moreover, although HDL‐C is crucial for maintaining vascular health and preventing cardiovascular diseases, this large‐scale study did not evaluate other atherogenic lipoproteins, such as apolipoprotein A (Apo A) [19]. Only a few cross‐sectional studies have investigated the association of apolipoprotein B (Apo B) with BMD or osteoporosis, and these findings are varied by specific sites and sex [20, 21]. Previous studies have also reported the associations of other lipid profiles, such as low‐density lipoprotein cholesterol (LDL‐C) [10, 11, 12, 13, 18], total cholesterol (TC) [11, 13, 16, 18] and triglycerides (TG) [13, 16, 18], but these were not confirmed by the analysis of ASPREE data [9]. Notably, these studies are limited by the small sample size [10, 11, 12, 13, 14], cross‐sectional nature [11, 12, 13] or restriction to a single lipid trait [12, 14]. Furthermore, few investigations have explored the underlying mechanisms of these relationships using population‐based data [16, 18].

To address these gaps in the evidence, we used large‐scale prospective data from the UK Biobank to systematically quantify the relationships between serum lipid traits and the risks of fractures and osteoporosis. Additionally, we examined to what extent BMD may mediate any such associations, assuming causality.

## 2 Methods

### 2.1 Study Design and Participants

The UK Biobank, a large‐scale prospective population study conducted from April 2007 to December 2010, recruited 502 366 participants aged 40–69 years, achieving a 5.5% response rate. Recruitment took place at 22 assessment centres across England, Scotland and Wales. During baseline assessments, participants completed a self‐administered touchscreen questionnaire, underwent face‐to‐face interviews and had various physical measurements taken by trained staff, including height, weight and blood pressure. Additionally, they participated in computer‐assisted interviews and provided biological samples as part of the comprehensive assessment. Participants' self‐reported data spanned areas such as ethnicity, smoking status, alcohol intake, medical history and regular medication use, with comorbidities and medical history further verified during interviews. With participant consent, these baseline data were linked to hospital admission data and mortality records, as well as to primary care data where available. This extensive linkage supports detailed long‐term follow‐up and enables a comprehensive study of health outcomes. Our analysis was based on data acquired in November 2023.

We excluded participants with missing serum lipid measurements or age information. In the analyses of the risk of fractures, we further excluded participants who reported prevalent fractures at baseline. In the analyses of the risk of osteoporosis, we further excluded participants without primary care data on osteoporosis incidence and those with a history of osteoporosis at baseline (Figure S1).

### 2.2 Exposure Measurements

In the UK Biobank study, an extensive evaluation of serum lipid characteristics was undertaken using blood specimens acquired during initial participant enrolment. This evaluation encompassed the analysis of a range of lipid traits, including apolipoprotein A (Apo A), apolipoprotein B (Apo B), total cholesterol (TC), high‐density lipoprotein cholesterol (HDL‐C), low‐density lipoprotein cholesterol (LDL‐C), triglycerides (TG) and lipoprotein A (Lp(a)). Apo A measured in our study is specifically Apolipoprotein A1. The UK Biobank Data‐Field IDs used in the study were described in the Supplementary Methods. These lipid traits were quantified through immunoturbidimetric analysis using the Beckman Coulter AU5800, an automated haematology analyser. The apolipoproteins were measured in g/L, circulating cholesterol and TG in mmol/L, and Lp(a) in nmol/L. The UK Biobank has detailed the methodologies for handling these serum samples and conducting the necessary assays [22]. The UK Biobank study collected and processed biological samples, gathering 45 mL of blood and 9 mL of urine from each participant using the vacutainer system. Automated processes and a detailed Laboratory Information Management System were employed to ensure precision and uniformity in data handling, focusing on standardization and stringent quality control. This systematic approach guaranteed consistent and reliable measurement of the serum lipid profiles within this extensive participant group.

### 2.3 Outcome Ascertainment

The primary outcomes investigated were the incidence of fractures and osteoporosis. Additional outcomes included major osteoporotic, hip and clinical vertebral fractures. Incidence of fracture was determined using hospital admission data or death certificates from England (covering the period from 1997 to September 2021), Scotland (from 1981 to July 2021) and Wales (from 1998 to February 2018). Follow‐up of all participants ended on death, the final date of the available hospital admission data or upon the diagnosis of an incident fracture, whichever occurred first.

Incidence of osteoporosis was ascertained through primary care data, hospital admission records and death certificate to ensure the inclusion of osteoporosis that did not require hospital admission. Hence, the analysis of osteoporosis risk was confined to participants with available linked primary care records. At the time of the study, primary care data encompassed about 45% of the UK Biobank cohort, equating to approximately 230 000 participants, with the selection purely based on the computing system used by general practices. Detailed information on the procedures for linking primary care records can be found at http://biobank.ndph.ox.ac.uk/showcase/showcase/docs/primary_care_data.pdf. The primary care data cut‐off dates were May 2017 for Scotland, September 2017 for Wales and August 2017 for England. For these individuals, follow‐up was terminated at either the time of death, the final date of primary care data for their respective country or upon an osteoporosis diagnosis, whichever occurred first.

The specific International Classification of Diseases and Related Health Problems, 10th Revision (ICD‐10) codes used for identifying fractures and osteoporosis were predetermined and are detailed in Supplementary Table S1.

### 2.4 Mediators

Based on findings of Mendelian randomization research [16, 23], this study included three possible mediators for fractures: (1) bone mineral density of the right femoral neck (FN), (2) bone mineral density of the left FN and (3) bone mineral density of the lumbar spine (LS).

In 2014, a subset of UK Biobank participants was invited for additional imaging studies, including abdominal MRI and dual‐energy X‐ray absorptiometry (DXA). By early 2020, over 45 000 people had completed a DXA scan [24]. DXA provides accurate measurements of bone mineral density at specific sites, such as the proximal femur and lumbar spine, as well as an overall assessment of body composition, including bone, fat and lean mass. The imaging enhancement employs an iDXA instrument (GE‐Lunar, Madison, WI, USA) to perform comprehensive scans of various body regions within a 20‐minute protocol. Bone mineral density measurements were automatically derived from the scanner and directly transferred to the UK Biobank with minimal post‐processing required [24]. The BMD data measured in 2014 were used for the mediation analysis in our study.

### 2.5 Statistical Analyses

The baseline characteristics are presented as means with standard deviations (SDs) for continuous variables, and as counts with percentages for categorical variables. Non‐linear associations were investigated by Cox proportional hazard models with exposure variables fitted on penalized cubic splines. The penalized spline is a variant of the basic spline that differs from restricted cubic splines regarding knot placement and number sensitivity [25]. The medians of each serum lipid trait were set as reference points. Likelihood ratio tests were employed to assess the non‐linearity of exposure–outcome associations and overall statistical significance. Exposures were also evaluated as continuous variables with hazard ratios (HRs) and 95% confidence intervals (CIs) determined at one standard deviation (SD) increments, and as categorical variables using the lowest quintile as the reference for HR calculation in the multivariable Cox proportional hazards models. We verified the proportional hazards assumption using statistical tests based on Schoenfeld residuals, which indicated that age and sex violated this assumption. To address this, we treated age and sex as strata in the subsequent analysis. Since the hazard ratio represents an average of the actual hazard ratios over the entire follow‐up period, we assessed the short‐ and long‐term risks of fracture and osteoporosis associated with serum lipid concentrations over follow‐up periods of 1, 2, 5 and 10 years.

Age, sex, ethnicity and deprivation index were adjusted for in the minimally adjusted model. In the final model, we additionally adjusted for lifestyle behaviours, obesity‐related markers, health conditions and medication history. The definitions of covariates and types of variables included in the models were described in the Supplementary Methods. Because we are interested in the relative hazard by lipid traits, cause‐specific HRs were used to address competing risks related to death from other causes, for which individuals were censored at the time of death [26]. We did not account for fractures as a competing risk because the occurrence of a fracture does not prevent the development of osteoporosis. Furthermore, we assessed the proportion of fractures or osteoporosis attributable to serum lipid levels above the 1st quintile, the population attributable fraction estimates for quintiles 2–5 were aggregated.

### 2.6 Subgroup Analyses

Subgroup analyses were performed according to baseline characteristics like age (<60 or ≥60 years), sex (male or female), BMI category, presence of CVD and use of lipid‐lowering medications. The associations between serum lipid traits and fractures were examined using Cox regression models with penalized cubic splines to determine whether there was evidence of a non‐linear association in each subgroup. The model incorporated interaction terms between these stratifying factors and serum lipid concentrations (per quintile and per 1 standard deviation increase) to investigate the potential modifying effects on a multiplicative scale. The ratio of hazard ratios (RHR) and their corresponding 95% confidence intervals for each risk factor were reported.

### 2.7 Mediation Analyses

In the mediation analysis, fracture outcomes only included those that occurred after DXA measurements to ensure temporality. The g‐formula approach was used to evaluate the mediating role of BMD in the relationship between serum lipid traits and fracture risk, considering all baseline confounders in the fully adjusted model [27]. Given that cardiovascular disease [28] and cancer [29] can be influenced by serum lipid traits, these factors, which occur after baseline but before the imaging assessment, were accounted for as post‐exposure confounders (Figure S2). Assuming causality post‐adjustment, the total effects (TEs) of serum lipid traits on fracture risk were segregated into natural direct and indirect effects (NDEs and NIEs), with the latter depicting the portion mediated through BMD. The 95% confidence interval and *p* values were estimated using non‐parametric bootstrapping (500 times). The proportion of mediation was calculated using the formula: NDE × (NIE − 1) / (TE − 1), to quantify the extent to which the association between variations in serum lipid traits and fracture risk could be attributed to BMD.

A two‐tailed significance level of *p* < 0.05 was considered statistically significant. Statistical analyses were performed using the Stata Version 16.0 (StataCorp LP, College Station, TX) and R version 4.3.1 with the CMAverse package [30].

## 3 Results

A total of 414 302 participants from the UK Biobank database were included after excluding those with missing data on serum lipid traits (*n* = 88 049) or baseline age (*n* = 21). After further excluding participants with fractures (*n* = 30 772) at baseline, the analysis of fracture risk included 383 530 participants. For the osteoporosis analysis, participants without linkage to primary care data (*n* = 223 827) and those with osteoporosis (*n* = 5556) at baseline were excluded, leaving a total of 184 919 participants (Figure S1). Over a median follow‐up period of 13.8 years (interquartile range [IQR] = 13.1 to 14.5 years), 25 918 participants (6.8%) experienced any fractures, and 7591 individuals (4.1%) were diagnosed with osteoporosis.

Table 1 shows the baseline demographic characteristics by sex for the 414 302 participants included in the fracture models. Among them, 54% were women, with an average age of 56.6 years. Women had a lower prevalence of smoking and units of alcohol intake and performed less physical activity. Additionally, they consumed less processed meat and were less likely to have lipid‐lowering drugs. On the other hand, they had higher prevalent frailty or pre‐frailty, cancer, osteoporosis and a history of fractures. Except for TG, women typically presented with higher concentrations of lipid traits than men.

**TABLE 1:** Baseline characteristics after excluding those with all lipid traits missing at baseline in the UK Biobank, by sex.

Baseline characteristics	Total (*N* = 414 302)	Women (*N* = 223 060)	Men (*N* = 191 242)
Sociodemographics			
Age, years	56.6 (8.1)	56.4 (8.0)	56.8 (8.2)
Ethnicity			
White	390 490 (94.3%)	210 472 (94.4%)	180 018 (94.1%)
Non‐white	21 853 (5.3%)	11 682 (5.2%)	10 171 (5.3%)
Deprivation index	−1.3 (3.1)	−1.3 (3.0)	−1.3 (3.1)
Lifestyle behaviours			
Current smokers	43 626 (10.5%)	19 875 (8.9%)	23 751 (12.4%)
Alcohol intake, units/week	16.3 (18.9)	10.0 (11.3)	23.5 (22.8)
Sleep duration, h/day	7.2 (1.1)	7.2 (1.1)	7.1 (1.1)
Total physical activity, MET‐h/week	2409.7 (2443.6)	2248.7 (2284.0)	2595.9 (2603.6)
Total sedentary behaviour, h/day	5.1 (2.3)	4.7 (2.0)	5.5 (2.4)
Fruit and vegetables intake, portions/week	4.1 (2.4)	4.4 (2.4)	3.8 (2.5)
Red meat intake, portions/week	2.1 (1.5)	2.0 (1.4)	2.3 (1.5)
Processed meat intake, frequency/week	1.5 (1.4)	1.1 (1.2)	1.9 (1.5)
Oily fish intake, frequency/week	1.1 (1.0)	1.1 (1.0)	1.1 (1.1)
Ever eats eggs	404 422 (97.6%)	218 045 (97.8%)	186 377 (97.5%)
Ever eats dairy	406 400 (98.1%)	218 636 (98.0%)	187 764 (98.2%)
Obesity‐related markers			
Body‐mass index	27.4 (4.8)	27.1 (5.2)	27.8 (4.2)
Under weight (<18.5 kg/m^2^)	659 (0.2%)	500 (0.2%)	159 (0.1%)
Normal weight (18.5–24.9 kg/m^2^)	135 235 (32.8%)	87 569 (39.4%)	47 666 (25.0%)
Overweight (25.0–29.9 kg/m^2^)	175 780 (42.6%)	81 654 (36.7%)	94 126 (49.5%)
Obese (≥30.0 kg/m^2^)	100 950 (24.5%)	52 549 (23.6%)	48 401 (25.4%)
Waist–hip ratio	0.9 (0.1)	0.8 (0.1)	0.9 (0.1)
Body fat percentage	31.4 (8.6)	36.6 (6.9)	25.3 (5.8)
Health status			
Number of morbidities			
0	142 573 (34.4%)	75 841 (34.0%)	66 732 (34.9%)
1–3	250 295 (60.4%)	134 705 (60.4%)	115 590 (60.4%)
≥4	21 434 (5.2%)	12 514 (5.6%)	8920 (4.7%)
Prefrail/frail^a^	173 859 (42.0%)	98 956 (44.4%)	74 903 (39.2%)
Cardiovascular disease	27 619 (6.7%)	8948 (4.0%)	18 671 (9.8%)
Hypertension	110 076 (26.6%)	52 152 (23.4%)	57 924 (30.3%)
Diabetes	20 628 (5.0%)	7623 (3.4%)	13 005 (6.8%)
Chronic kidney disease	1070 (0.3%)	495 (0.2%)	575 (0.3%)
Cancer	31 328 (7.6%)	19 734 (8.9%)	11 594 (6.1%)
Anaemia	16 987 (4.1%)	11 685 (5.2%)	5302 (2.8%)
Positive rheumatoid factor	3584 (0.9%)	2040 (0.9%)	1544 (0.8%)
Vitamin D deficiency	55 840 (13.5%)	29 440 (13.2%)	26 400 (13.8%)
Osteoporosis	7434 (1.8%)	5204 (2.3%)	2230 (1.2%)
History of fractures	30 772 (7.4%)	18 929 (8.5%)	11 843 (6.2%)
Falls in the last year	108 979 (26.4%)	58 650 (26.4%)	50 329 (26.5%)
Menopause	135 270 (32.7%)	135 270 (60.6%)	0 (0.0%)
Medication history			
Lipid‐lowering medications	72 357 (17.5%)	28 451 (12.8%)	43 906 (23.0%)
Hormone therapy	84 959 (20.5%)	84 959 (38.1%)	0 (0.0%)
Aspirin	58 020 (14.0%)	22 083 (9.9%)	35 937 (18.8%)
Glucocorticoids	3465 (0.8%)	1941 (0.9%)	1524 (0.8%)
Vitamin D supplements	16 582 (4.0%)	11 666 (5.2%)	4916 (2.6%)
Calcium supplements	28 754 (6.9%)	23 709 (10.6%)	5045 (2.6%)
Serum lipid measurements at baseline			
Apolipoprotein A, g/L	1.5 (0.3)	1.6 (0.3)	1.4 (0.2)
Apolipoprotein B, g/L	1.0 (0.2)	1.0 (0.2)	1.0 (0.2)
Total cholesterol, mmol/L	5.7 (1.1)	5.9 (1.1)	5.5 (1.1)
HDL cholesterol, mmol/L	1.4 (0.4)	1.6 (0.4)	1.3 (0.3)
LDL cholesterol, mmol/L	3.6 (0.9)	3.6 (0.9)	3.5 (0.9)
Triglycerides, mmol/L	1.7 (1.0)	1.5 (0.8)	2.0 (1.1)
Lipoprotein A, nmol/L	50.9 (60.6)	53.4 (61.9)	48.0 (58.9)

*Note:* MET = Metabolic equivalent; HDL = High density lipoprotein; LDL = Low density lipoprotein. Data are mean (SD) or n (%) for continuous and categorical variables, as appropriate. ^a^ According to the modified Fried frailty phenotype, a person is considered prefrail or frail if they meet two or more of these criteria: weight loss, exhaustion, decreased physical activity, slow walking speed, and decreased grip strength.

### 3.1 Risks of Fractures

**FIGURE 1:**
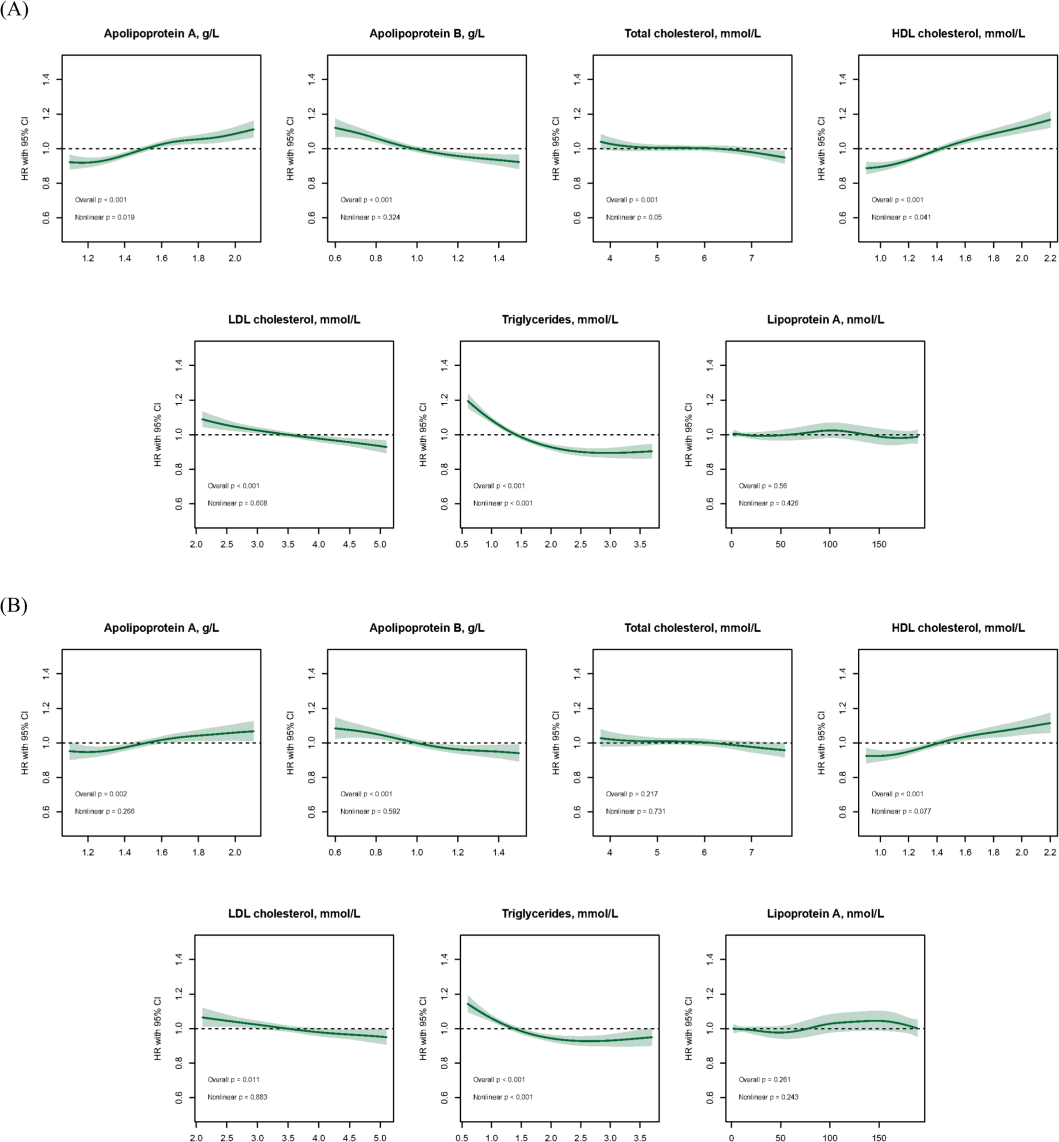
Associations between lipid traits and the risk of (A) fractures and (B) osteoporosis. Notes: Model was adjusted for age, sex, ethnicity, deprivation index, current smokers, alcohol intake, sleep duration, total physical activity, total sedentary behaviour, fruit and vegetables intake, red meat intake, processed meat intake, oily fish intake, ever eats eggs, ever eats dairy, body‐mass index, waist‐hip ratio, body fat percentage, number of morbidities, prefrail/frail status, cardiovascular disease, hypertension, diabetes, chronic kidney disease, cancer, anaemia, positive rheumatoid factor, vitamin D deficiency, osteoporosis (excluded in the osteoporosis model), history of fractures (excluded in the fracture model), falls in the last year, lipid‐lowering medications, aspirin, glucocorticoids, vitamin D supplements and calcium supplements.

**FIGURE 2:**
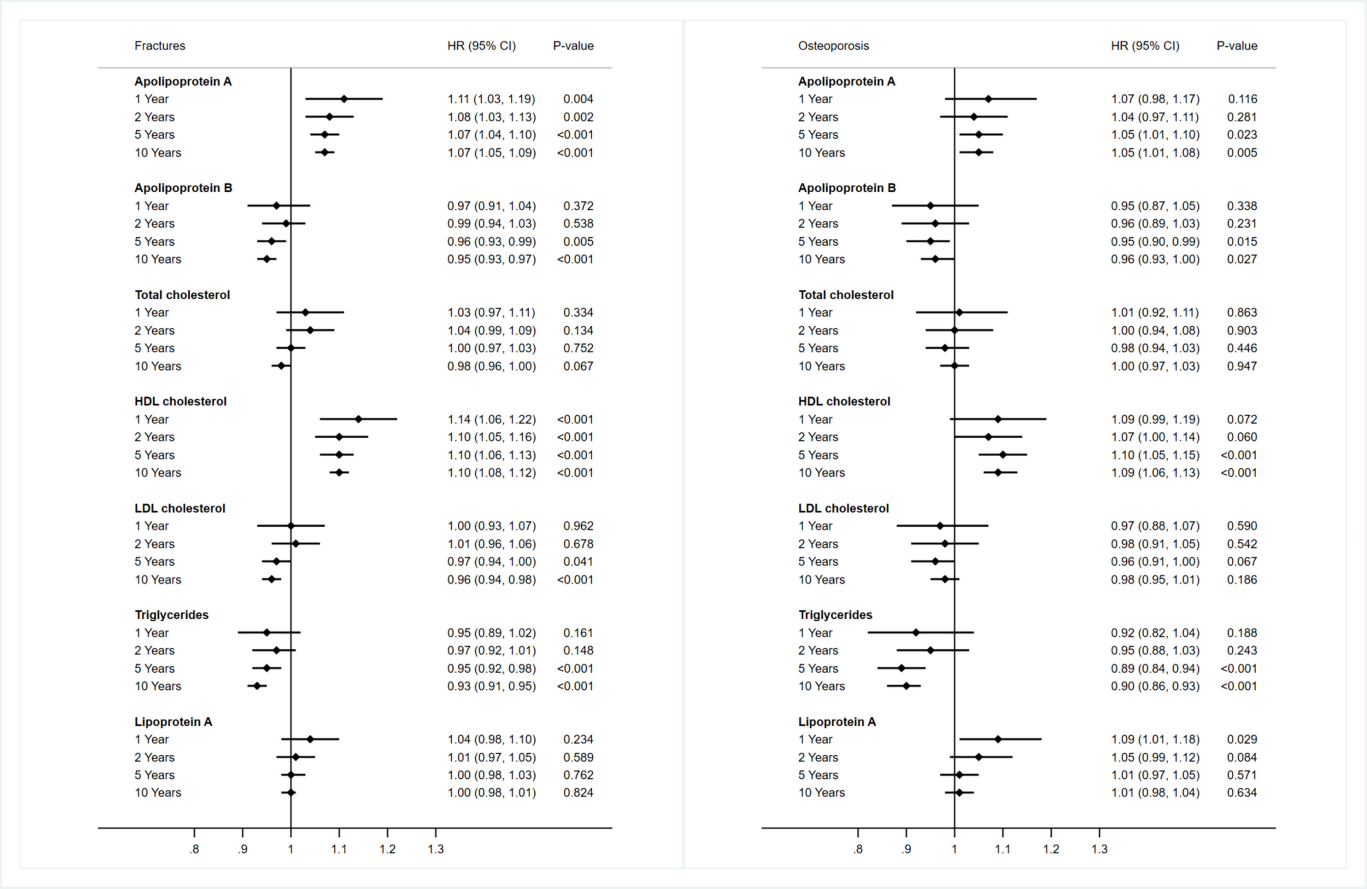
Associations between lipid traits and the risk of fractures and osteoporosis over different follow‐up periods from baseline. Notes: Model was adjusted for age, sex, ethnicity, deprivation index, current smokers, alcohol intake, sleep duration, total physical activity, total sedentary behaviour, fruit and vegetables intake, red meat intake, processed meat intake, oily fish intake, ever eats eggs, ever eats dairy, body‐mass index, waist‐hip ratio, body fat percentage, number of morbidities, prefrail/frail status, cardiovascular disease, hypertension, diabetes, chronic kidney disease, cancer, anaemia, positive rheumatoid factor, vitamin D deficiency, osteoporosis (excluded in the osteoporosis model), history of fractures (excluded in the fracture model), falls in the last year, lipid‐lowering medications, aspirin, glucocorticoids, vitamin D supplements and calcium supplements.

Figure 1A shows the fully adjusted associations between serum lipid traits and fracture risk. Supplementary Figure S3A displays the results for minimally adjusted models. Lp(a) was not significantly associated with fracture risk. All other serum lipid traits, except for Apo B and LDL‐C, demonstrated non‐linear associations with the risk of fractures. The risk of fractures was observed to be higher in participants within the highest quintile of Apo A (6.25 vs. 4.19 per 1000 person‐years, HR 1.15 [95% CI 1.10, 1.21], *p* < 0.001) and HDL‐C (6.25 vs. 4.17, HR 1.27 [1.20, 1.34], *p* < 0.001), compared to those in the lowest quintile, respectively. Apo B (non‐linear *p* = 0.32) and LDL‐C (non‐linear *p* = 0.61) showed a negative linear association with fracture risk. When modelled as continuous measures, the HR (95% CI) per SD was 0.95 (0.93, 0.96) (*p* < 0.001) for Apo B and 0.96 (0.94, 0.97) (*p* < 0.001) for LDL‐C. Regarding TC (HR 0.94 [0.89, 0.99], *p* = 0.015) and TG (HR 0.78 [0.74, 0.82], *p* < 0.001), a lower risk of fractures was observed in the highest quintile compared with the lowest quintile (Table 2). When restricting the follow‐up time, Apo A and HDL‐C were associated with increased fracture risks across all follow‐up periods. The associations of Apo B, TC, LDL‐C and TG with fracture risks were attenuated in the analyses restricted to the first two years of follow‐up (Figure 2). The proportion of fractures attributable to elevated lipid traits is shown in Supplementary Table S2. Compared to individuals in the 1st quintile, the combined proportion of fractures attributable to HDL‐C concentrations for individuals in quintiles 2–5 was 11.65% (8.45%, 14.73%).

**TABLE 2:** Associations between serum lipid concentrations and the risk of fractures and osteoporosis in the UK Biobank.

Lipid traits	Fracture				Osteoporosis			
	Event	Crude incidence rate (95% CI)	HR (95% CI)	*p*	Event	Crude incidence rate (95% CI)	HR (95% CI)	*p*
Apolipoprotein A								
Quintile 1	4425	4.19 (4.07, 4.32)	Reference		905	1.73 (1.62, 1.85)	Reference	
Quintile 2	4518	4.36 (4.23, 4.49)	0.99 (0.95, 1.04)	0.808	1082	2.13 (2.00, 2.26)	0.97 (0.87, 1.08)	0.556
Quintile 3	5122	4.96 (4.83, 5.10)	1.09 (1.04, 1.14)	<0.001	1411	2.79 (2.65, 2.94)	1.03 (0.93, 1.13)	0.618
Quintile 4	5583	5.48 (5.33, 5.62)	1.12 (1.07, 1.18)	<0.001	1821	3.70 (3.53, 3.87)	1.06 (0.96, 1.17)	0.239
Quintile 5	6270	6.25 (6.09, 6.40)	1.15 (1.10, 1.21)	<0.001	2372	4.99 (4.79, 5.20)	1.13 (1.02, 1.25)	0.018
Per 1‐SD increase	NA	NA	1.06 (1.04, 1.08)	<0.001	NA	NA	1.04 (1.02, 1.07)	0.002
Apolipoprotein B								
Quintile 1	5651	5.48 (5.34, 5.63)	Reference		1547	3.12 (2.96, 3.28)	Reference	
Quintile 2	5227	5.05 (4.92, 5.19)	0.92 (0.88, 0.96)	<0.001	1535	3.07 (2.92, 3.23)	0.94 (0.86, 1.02)	0.130
Quintile 3	5065	4.92 (4.79, 5.06)	0.91 (0.87, 0.95)	<0.001	1518	3.06 (2.91, 3.22)	0.92 (0.85, 1.00)	0.056
Quintile 4	5018	4.89 (4.75, 5.02)	0.88 (0.84, 0.92)	<0.001	1492	2.99 (2.84, 3.15)	0.88 (0.81, 0.96)	0.004
Quintile 5	4957	4.83 (4.70, 4.97)	0.85 (0.81, 0.89)	<0.001	1499	2.93 (2.78, 3.08)	0.86 (0.79, 0.94)	0.001
Per 1‐SD increase	NA	NA	0.95 (0.93, 0.96)	<0.001	NA	NA	0.95 (0.92, 0.98)	<0.001
Total cholesterol								
Quintile 1	5322	5.13 (4.99, 5.27)	Reference		1282	2.54 (2.40, 2.68)	Reference	
Quintile 2	5000	4.83 (4.70, 4.96)	0.98 (0.94, 1.03)	0.509	1365	2.74 (2.60, 2.89)	1.00 (0.91, 1.09)	0.951
Quintile 3	4972	4.82 (4.69, 4.96)	0.97 (0.92, 1.01)	0.155	1517	3.05 (2.90, 3.21)	1.02 (0.93, 1.12)	0.625
Quintile 4	5208	5.07 (4.94, 5.21)	0.98 (0.93, 1.03)	0.431	1617	3.24 (3.08, 3.40)	0.98 (0.89, 1.07)	0.608
Quintile 5	5416	5.33 (5.18, 5.47)	0.94 (0.89, 0.99)	0.015	1810	3.60 (3.43, 3.77)	0.95 (0.86, 1.04)	0.255
Per 1‐SD increase	NA	NA	0.97 (0.96, 0.99)	0.002	NA	NA	0.98 (0.95, 1.01)	0.185
HDL cholesterol								
Quintile 1	4394	4.17 (4.05, 4.30)	Reference		830	1.59 (1.49, 1.71)	Reference	
Quintile 2	4633	4.45 (4.32, 4.58)	1.05 (1.00, 1.10)	0.057	1098	2.15 (2.03, 2.28)	1.04 (0.93, 1.15)	0.502
Quintile 3	5056	4.92 (4.79, 5.06)	1.12 (1.07, 1.18)	<0.001	1355	2.70 (2.56, 2.85)	1.08 (0.97, 1.20)	0.146
Quintile 4	5556	5.44 (5.30, 5.58)	1.19 (1.13, 1.25)	<0.001	1842	3.73 (3.57, 3.91)	1.21 (1.09, 1.34)	<0.001
Quintile 5	6279	6.25 (6.10, 6.41)	1.27 (1.20, 1.34)	<0.001	2466	5.17 (4.96, 5.37)	1.31 (1.18, 1.46)	<0.001
Per 1‐SD increase	NA	NA	1.09 (1.07, 1.11)	<0.001	NA	NA	1.09 (1.06, 1.12)	<0.001
LDL cholesterol								
Quintile 1	5602	5.43 (5.29, 5.58)	Reference		1465	2.93 (2.78, 3.08)	Reference	
Quintile 2	5156	4.99 (4.86, 5.13)	0.95 (0.91, 0.99)	0.024	1491	3.01 (2.86, 3.17)	0.99 (0.90, 1.07)	0.732
Quintile 3	5013	4.86 (4.73, 5.00)	0.93 (0.89, 0.98)	0.004	1525	3.07 (2.92, 3.23)	0.99 (0.90, 1.08)	0.772
Quintile 4	5022	4.88 (4.74, 5.01)	0.91 (0.87, 0.96)	<0.001	1491	2.97 (2.82, 3.13)	0.91 (0.83, 0.99)	0.035
Quintile 5	5125	5.01 (4.87, 5.15)	0.89 (0.85, 0.93)	<0.001	1619	3.18 (3.02, 3.34)	0.91 (0.83, 1.00)	0.056
Per 1‐SD increase	NA	NA	0.96 (0.94, 0.97)	<0.001	NA	NA	0.96 (0.93, 0.99)	0.004
Triglycerides								
Quintile 1	5425	5.25 (5.11, 5.40)	Reference		1731	3.54 (3.37, 3.71)	Reference	
Quintile 2	5392	5.26 (5.12, 5.41)	0.91 (0.87, 0.94)	<0.001	1732	3.50 (3.33, 3.66)	0.92 (0.85, 0.99)	0.022
Quintile 3	5282	5.15 (5.01, 5.29)	0.87 (0.83, 0.91)	<0.001	1571	3.16 (3.01, 3.32)	0.87 (0.80, 0.94)	<0.001
Quintile 4	5050	4.90 (4.77, 5.04)	0.81 (0.78, 0.85)	<0.001	1468	2.89 (2.75, 3.04)	0.85 (0.78, 0.93)	<0.001
Quintile 5	4769	4.61 (4.48, 4.74)	0.78 (0.74, 0.82)	<0.001	1089	2.12 (2.00, 2.25)	0.75 (0.68, 0.82)	<0.001
Per 1‐SD increase	NA	NA	0.93 (0.91, 0.94)	<0.001	NA	NA	0.91 (0.88, 0.94)	<0.001
Lipoprotein A								
Quintile 1	5139	4.97 (4.83, 5.10)	Reference		1432	2.84 (2.69, 2.99)	Reference	
Quintile 2	5146	4.99 (4.86, 5.13)	1.02 (0.97, 1.06)	0.449	1402	2.78 (2.63, 2.93)	0.96 (0.89, 1.05)	0.359
Quintile 3	5145	5.01 (4.87, 5.14)	0.98 (0.94, 1.03)	0.435	1537	3.07 (2.92, 3.23)	0.93 (0.86, 1.01)	0.105
Quintile 4	5232	5.08 (4.95, 5.22)	1.01 (0.97, 1.06)	0.555	1596	3.24 (3.08, 3.40)	1.02 (0.94, 1.10)	0.646
Quintile 5	5256	5.12 (4.99, 5.26)	0.99 (0.95, 1.03)	0.673	1624	3.24 (3.09, 3.41)	0.97 (0.90, 1.05)	0.493
Per 1‐SD increase	NA	NA	1.00 (0.98, 1.01)	0.664	NA	NA	1.00 (0.98, 1.03)	0.990

*Note:* HR = Hazard ratio; CI = Confidence interval; HDL = High density lipoprotein; LDL = Low density lipoprotein; NA = Not available. The unit of crude incidence rate: events per 1000 person‐years. Model was adjusted for age, sex, ethnicity, deprivation index, current smokers, alcohol intake, sleep duration, total physical activity, total sedentary behaviour, fruit and vegetables intake, red meat intake, processed meat intake, oily fish intake, ever eats eggs, ever eats dairy, body‐mass index, waist–hip ratio, body fat percentage, number of morbidities, prefrail/frail status, cardiovascular disease, hypertension, diabetes, chronic kidney disease, cancer, anaemia, positive rheumatoid factor, vitamin D deficiency, osteoporosis (excluded in the osteoporosis model), history of fractures (excluded in the fracture model), falls in the last year, lipid‐lowering medications, aspirin, glucocorticoids, vitamin D supplements and calcium supplements.

When stratifying the associations between serum lipid traits and fracture risk by subgroups, a significant interaction was observed for the association of fracture risk with Apo A by sex, with Apo B by age, CVD and use of lipid‐lowering medications, with TC by CVD and use of lipid‐lowering medications, with HDL‐C by age, sex, and CVD, with LDL‐C by CVD and use of lipid‐lowering medications, and with TG by age (Supplementary Figure S4 and Supplementary Tables S3–S7). The highest quintiles of HDL‐C were associated with an increased risk of fractures in both sexes, compared with the lowest quintile, particularly among men (Table S4). HDL‐C was linearly associated with an increased risk of fractures in both younger and older, irrespective of cardiovascular disease status or lipid‐lowering medication use. Elevated concentrations of Apo B, TC and LDL‐C were significantly associated with a reduced risk of fractures in individuals without CVD but not in those with CVD (Figure S4 and Table S6).

Table 3 shows the results of the mediation analyses. BMD of the right/left femoral neck or lumbar spine potentially explained over 10% of the excess fracture risk attributed to the effects of Apo A (right FN: 13.50%; left FN: 12.60%; LS: 9.40%), Apo B (right FN: 5.30%; left FN: 6.90%; LS: 13.90%), TC (right FN: 27.50%; left FN: 23.50%; LS: 40.30%), HDL‐C (right FN: 14.80%; left FN: 15.30%; LS: 12.40%) and LDL‐C (right FN: 12.30%; left FN: 13.70%; LS: 19.70%).

**TABLE 3:** Mediation analysis of the relationship between lipid traits and fracture risk through right/left femoral neck and lumbar spine BMD.

Mediators	Total Effect		Direct Effect		Indirect Effect		% mediation
	HR (95% CI)	*p*	HR (95% CI)	*p*	HR (95% CI)	*p*	
Right femoral neck BMD							
Apolipoprotein A	1.38 (1.07, 1.81)	0.010	1.33 (1.04, 1.76)	0.030	1.04 (1.02, 1.06)	<0.001	13.50
Apolipoprotein B	0.72 (0.52, 1.00)	0.048	0.71 (0.51, 0.97)	0.020	1.02 (1.00, 1.04)	0.008	5.30
Total cholesterol	0.96 (0.89, 1.02)	0.240	0.95 (0.89, 1.01)	0.140	1.01 (1.00, 1.01)	<0.001	27.50
HDL cholesterol	1.32 (1.08, 1.60)	0.008	1.27 (1.05, 1.54)	0.030	1.04 (1.02, 1.05)	<0.001	14.80
LDL cholesterol	0.93 (0.84, 1.01)	0.090	0.92 (0.83, 1.00)	0.052	1.01 (1.00, 1.02)	0.004	12.30
Triglycerides	0.92 (0.84, 1.00)	0.040	0.93 (0.85, 1.00)	0.060	1.00 (0.99, 1.00)	0.060	4.10
Lipoprotein A	1.00 (1.00, 1.00)	0.520	1.00 (1.00, 1.00)	0.170	1.00 (1.00, 1.00)	0.970	NA
Left femoral neck BMD							
Apolipoprotein A	1.38 (1.05, 1.82)	0.020	1.33 (1.01, 1.76)	0.040	1.04 (1.02, 1.06)	<0.001	12.60
Apolipoprotein B	0.73 (0.52, 0.99)	0.040	0.71 (0.50, 0.97)	0.020	1.03 (1.01, 1.05)	0.008	6.90
Total cholesterol	0.96 (0.89, 1.03)	0.240	0.95 (0.89, 1.02)	0.160	1.01 (1.01, 1.02)	<0.001	23.50
HDL cholesterol	1.32 (1.09, 1.59)	0.008	1.27 (1.04, 1.53)	0.020	1.04 (1.02, 1.06)	<0.001	15.30
LDL cholesterol	0.93 (0.85, 1.01)	0.100	0.92 (0.84, 1.00)	0.040	1.01 (1.01, 1.02)	<0.001	13.70
Triglycerides	0.92 (0.85, 1.00)	0.040	0.92 (0.85, 1.00)	0.040	1.00 (0.99, 1.00)	0.410	3.60
Lipoprotein A	1.00 (1.00, 1.00)	0.570	1.00 (1.00, 1.00)	0.200	1.00 (1.00, 1.00)	0.940	NA
Lumbar spine BMD							
Apolipoprotein A	1.38 (1.07, 1.80)	0.004	1.35 (1.04, 1.75)	0.010	1.03 (1.01, 1.04)	<0.001	9.40
Apolipoprotein B	0.73 (0.54, 0.98)	0.040	0.69 (0.51, 0.93)	0.010	1.05 (1.04, 1.08)	<0.001	13.90
Total cholesterol	0.96 (0.90, 1.03)	0.260	0.95 (0.88, 1.01)	0.110	1.02 (1.01, 1.02)	<0.001	40.30
HDL cholesterol	1.32 (1.05, 1.61)	0.004	1.28 (1.02, 1.57)	0.010	1.03 (1.02, 1.05)	<0.001	12.40
LDL cholesterol	0.92 (0.84, 1.02)	0.120	0.91 (0.82, 1.00)	0.040	1.02 (1.01, 1.02)	<0.001	19.70
Triglycerides	0.92 (0.85, 1.00)	0.052	0.92 (0.85, 1.00)	0.052	1.00 (1.00, 1.00)	0.950	1.80
Lipoprotein A	1.00 (1.00, 1.00)	0.410	1.00 (1.00, 1.00)	0.160	1.00 (1.00, 1.00)	1.000	NA

*Note:* BMD = Bone mineral density; CI = confidence interval; HDL = High density lipoprotein; LDL = Low density lipoprotein; NA = Not available. Mediation analysis was adjusted for various factors, including age, sex, ethnicity, deprivation index, current smoking status, alcohol intake, sleep duration, total physical activity, total sedentary behaviour, fruit and vegetable intake, red meat intake, processed meat intake, oily fish intake, ever eats eggs, ever eats dairy, body mass index, waist–hip ratio, body fat percentage, number of morbidities, prefrail/frail status, cardiovascular disease, hypertension, diabetes, chronic kidney disease, cancer, anaemia, positive rheumatoid factor, vitamin D deficiency, osteoporosis, falls in the last year, and use of lipid‐lowering medications, aspirin, glucocorticoids, vitamin D supplements and calcium supplements as confounders before baseline. Cardiovascular disease and cancer after baseline were adjusted for as confounders affected by the exposure as well.

### 3.2 Risks of Osteoporosis

Figure 1B illustrates the associations between serum lipid traits and the risk of osteoporosis, adjusted for all relevant factors. Supplementary Figure S3B presents these associations in models with minimal adjustments. No association was found between Lp(a) and TC with the risk of osteoporosis. Apo A (non‐linear *p* = 0.27), Apo B (non‐linear *p* = 0.60), HDL‐C (non‐linear *p* = 0.08) and LDL‐C (non‐linear *p* = 0.88) showed linear associations with osteoporosis, while TG displayed a non‐linear association. After multivariable adjustment, each 1‐SD increase in Apo A was associated with a 4% increase in osteoporosis risk (HR 1.04 [1.02, 1.07], *p* = 0.002). Each 1‐SD increment in Apo B (HR, 0.95 [0.92, 0.98], *p* < 0.001) and LDL‐C (HR 0.96 [0.93, 0.99], *p* = 0.004) was associated with lower risks of osteoporosis, while each 1‐SD increment in HDL‐C (HR 1.09 [1.06, 1.12], *p* < 0.001) was associated with a higher risk of osteoporosis. Individuals in the highest quintile of TG had a lower risk (2.12 vs. 3.54, HR 0.75 [0.68, 0.82], *p* < 0.001) (Table 2). When restricting the follow‐up time, Apo A, Apo B, HDL‐C and TG were only associated with osteoporosis risk with five years or longer follow‐up time. Lp(a) was positively associated with osteoporosis risk only restricting to the first year of follow‐up, and TC and LDL‐C were not clearly associated with osteoporosis risk (Figure 2). The proportion of osteoporosis attributable to elevated lipid traits is shown in Supplementary Table S2. Compared to individuals in the 1st quintile, the combined proportion of osteoporosis attributable to HDL‐C concentrations for individuals in quintiles 2–5 was 14.05% (6.44%, 21.04%).

When stratifying the associations between serum lipid traits and osteoporosis risk by subgroups, significant interactions were observed. The associations of Apo B, HDL‐C and LDL‐C with osteoporosis risk were significantly moderated by age, sex and lipid‐lowering medication use, while that of TG was only significantly moderated by age (Figure S5 and Supplementary Tables S8–S12). Apo A was linearly associated with an increased risk of osteoporosis in both age groups, particularly among the younger (Supplementary Figure S5 and Supplementary Table S8). Compared with those in the lowest quintile of HDL‐C, men in the highest quintile had an around 101% increase in the risk of osteoporosis (2.18 vs. 0.92, HR 2.01 [1.60, 2.52], *p* < 0.001), while women in the highest quintile had an almost 19% increase (5.84 vs. 3.86, HR 1.19 [1.05, 1.36], *p* = 0.007). A higher concentration of LDL‐C was associated with a lower risk of osteoporosis in men but not in women (Table S9).

### 3.3 Risks of Major Osteoporotic Fractures, Hip Fractures and Clinical Vertebral Fractures

The analyses for major osteoporosis, hip and clinical vertebral fractures showed results broadly consistent with those for all‐site fractures (Table S13). However, no association was found between Apo A and the risk of hip fractures. HDL‐C was only associated with a higher risk of hip fractures when modelled as a continuous variable (HR 1.05 [1.00, 1.09], *p* = 0.037). TC was not significantly associated with the risk of clinical vertebral fractures. The results of subgroup analyses, stratified by age, sex, BMI, presence of CVD and use of lipid‐lowering medications, were detailed in Supplementary Tables S14–S22.

## 4 Discussion

In this study, with a median follow‐up of 13 years over 400 000 individuals, we observed non‐linear associations between Apo A, HDL‐C and TG with fractures and between TG with osteoporosis. In contrast, Apo B and LDL‐C were linearly associated with these risks. Specifically, a single measurement of elevated concentrations of Apo A and HDL‐C were associated with higher risks of both future fractures and osteoporosis, whereas higher concentration of Apo B, LDL‐C and TG was associated with lower risks. The associations of Apo A and HDL‐C with fracture risk were stronger in men than in women. Additionally, BMD was observed to be a significant mediator for most associations between serum lipid traits and fractures, except for TG and Lp(a), assuming causality. Due to a potential loss of statistical power, no association was found between Apo A and the risk of hip fractures, and the association of HDL‐C with hip fractures was only marginally significant when modelled as a continuous variable. Altogether, our data support the notion that serum lipids could be biomarkers for skeletal health and are associated with significant clinical events such as osteoporosis and fractures.

Our findings underscore the complex and diverse relationships between serum lipid traits and the risk of fractures or osteoporosis. The relationships have been inconclusive despite extensive investigations [9, 10, 11, 12, 13, 14, 15, 16, 17]. A recent post‐hoc analysis of the ASPREE trial demonstrated a positive association between high HDL‐C concentrations and the risk of fractures in a healthy older population with no evident cardiovascular disease, dementia, physical disability or chronic illness expected to limit survival, which was consistent with our data [9]. However, a subsequent meta‐analysis found the association was only evident in older individuals, likely driven by the ASPREE trial [14]. Our data extend these findings to populations of younger ages and to those living with CVDs or physical disability in the UK. Moreover, the linearity of the association has been even more inconsistently reported, and the interpretation was mostly limited by the wide confidence intervals in prior research [9, 10]. The large sample size in our study provides us with a unique strength in exploring the non‐linearity of these associations.

Regarding other common lipids, namely LDL‐C, TG, and TC, no conclusion can be drawn due to highly heterogenous data. Taking LDL‐C as an example, studies have reported possible positive, negative or null associations [10, 13, 17]. Much of this evidence comes from cross‐sectional analyses where reverse causality is possible, making it not directly comparable with our findings. Research on the relationship between TG and TC and bone health has yielded mixed results [11, 13, 16]. A Mendelian randomization indicated that TG was positively associated with BMD, while no causal associations were identified between TC and BMD [16]. Other studies have found that higher TC and TG concentrations were associated with a higher risk of osteoporosis or lower BMD [11, 13]. Nonetheless, the evidence for the association of TC and TG is limited by the relatively small sample sizes and the cross‐sectional nature of these studies [11, 13]. Our large‐scale prospective cohort study suggested that TG were associated with a lower risk of osteoporosis and TC were not associated with osteoporosis. Lastly, very limited evidence is available for apolipoproteins and skeletal health outcomes. Our findings concur with recently published data that showed a positive association between skeletal health and Apo A [19]. Two studies using data from the National Health and Nutrition Examination Survey (NHANES) have investigated the association of Apo B with BMD or osteoporosis, and these findings vary by specific sites and sex [20, 21]. For instance, a positive association was observed between higher Apo B concentrations and femoral neck BMD, but no association was found for total femur BMD [21]. Another study reported a positive association between Apo B and the risk of osteopenia or osteoporosis, but only in males [20]. Due to the cross‐sectional nature of these studies, Apo B, BMD and osteoporosis were measured at a single point in time, making it difficult to establish a temporal relationship. Our prospective cohort study showed that higher Apo B concentrations were associated with lower risks of fracture and osteoporosis. These findings contribute to a broader understanding of the intricate interactions between Apo B and bone health, encouraging future research to re‐evaluate existing paradigms and explore new therapeutic targets for osteoporosis and fracture prevention.

We have identified signals suggesting sex differences in the associations between HDL‐C and skeletal health outcomes. These differences appear biologically plausible and may be linked to the influence of endogenous and exogenous sex hormones on lipid and lipoprotein metabolism [31]. Our findings are consistent with the ASPREE trial [9] and similar studies have shown that serum lipids impact the risk of CVD [32] and dementia [33] in men and women differently. Notably, sex hormones themselves play a crucial role in skeletal health [34]. Given these complex interrelationships, further research is imperative to unravel the associations among sex, sex hormones, serum lipid traits and skeletal health outcomes.

Our mediation analyses identified BMD as a significant mediator of the associations between several serum lipids and skeletal health outcomes. The data aligned with recent investigations linking serum lipids to BMD. Specifically, preclinical and genome‐wide association studies consistently reported a negative correlation between HDL‐C and BMD [16, 35]. In addition to HDL‐C, we also found that Apo A, Apo B, TC and LDL‐C may affect fracture risk via the BMD pathway, which has been less consistently suggested or largely uninvestigated before [16, 18, 36]. Nevertheless, it should be noted that the percentages of mediation via BMD at different sites were around 10–20% for most lipids, which could be due to the imprecision of the BMD measurements or other potential pathways, if the associations found were indeed causal. Other pathways through which lipids affect the risk of fractures or osteoporosis should be considered. Thus, further mechanistic investigations are warranted to fully understand these relationships.

Previous studies have expressed concerns about the confounding effects of physical activities and BMI that may bias the risk estimations [9, 18, 37, 38, 39]. Individuals with higher HDL‐C and Apo A concentrations might be more physically active, potentially engaging in activities that increase the risk of fractures, such as running or high‐impact sports [38]. Although we controlled for physical activity level, questionnaire‐based physical activity assessment is neither an accurate nor a precise measure to capture variations in activity type and intensity, particularly among those who are less active or engage in non‐traditional activities that are not recorded. Furthermore, the interaction between body weight and serum lipids traits adds complexity to assessing skeletal health. Heavier individuals typically exhibit lower concentrations of HDL‐C (or higher concentrations of TG and LDL‐C) and bear more weight, which can enhance bone strength and potentially improve BMD [37, 39]. In contrast, individuals with higher HDL‐C concentrations tend to be lighter, potentially experiencing reduced mechanical bone reinforcement due to their lower body weight [37, 39]. Our findings indicate that the potential effect of body weight on the association between lipid concentration and bone health might not be fully captured by BMI. Indeed, BMI does not differentiate between muscle and fat mass, nor does it account for the distribution of body weight, all of which can significantly influence bone health and fracture risk [40].

Interestingly, Lp(a) shows no significant correlation with fracture risks in our study. Lp(a) is genetically determined and shows no correlation with lifestyle factors [41], serving as a valuable comparison to understand the specific effects of other lipids that are more lifestyle‐dependent. Our findings, along with those of others, suggest the possible coexistence of two pathways explaining why higher HDL‐C (or Apo A) concentrations are associated with an increased risk of fractures: 1) individuals with higher HDL‐C (or Apo A) concentrations, indicative of more active lifestyles, may face increased fracture risks; 2) individuals with lighter body weight often exhibit higher HDL‐C (or Apo A) concentrations, accompanied by lower BMD due to decreased mechanical loading. These insights suggest that HDL‐C and Apo A may reveal subtle variations in physical activity and load elements more effectively compared to traditional metrics such as BMI, thereby establishing it as a potentially valuable marker of overall metabolic health and physical activity patterns.

In the current study, we investigated the associations between single measurements of serum lipids and risks of fractures and osteoporosis. With the long follow‐up time, we also identified patterns of potential time‐varying associations between several serum lipids at baseline and fractures and/or osteoporosis. The observed variations may be the results of latent effects of the serum lipids, effects of lipid regulating treatment, time‐varying confounding, or simply randomness in the analysis due to reduced statistical power. The time‐varying associations warrant further short‐term and long‐term longitudinal investigations on the underlying causal mechanisms.

Our findings on the associations between serum lipids and fractures or osteoporosis raise considerations regarding the potential impact of medications that modify serum lipid concentrations on bone health. For example, statins are widely used to effectively regulate serum LDL‐C concentrations. Our current findings of an inverse association between LDL‐C and fractures or osteoporosis do not support a protective role for statin treatment on these outcomes. Results from randomized controlled trials (RCTs) have demonstrated no effect of statins on fracture risk [42], and investigations into their effects on osteoporosis are still uncertain.

### 4.1 Strengths and Limitations of This Study

The strengths of this study are evident in its prospective design, large sample size, relatively prolonged follow‐up period and thorough evaluation of various covariates such as lifestyle factors, anthropometric measures, health status and medication history. This is so far the largest population‐based study that explored non‐linear associations between a wide range of serum lipid traits and skeletal health outcomes. Using mediation analyses, this study documents that BMD mediates some of these associations if they are causal. Several additional analyses were conducted to ensure the reliability of our findings. This study has several limitations. Firstly, the UK Biobank, our primary data source, exhibits a noticeable “healthy volunteer” selection bias, rendering it non‐representative of the general UK population [43]. Nevertheless, previous studies have shown exposure–outcome association to be broadly similar to that from population representative studies, adding confidence to our findings [43]. Secondly, our reliance on electronic health records for outcome ascertainment introduces the potential for misclassification, albeit likely to be non‐differential with respect to exposure status. This could result in underestimations of associations between serum lipids and skeletal health outcomes. Thirdly, there were no comprehensive measurements of fat and calcium intake. Instead, we used dietary information from participants who reported consuming eggs and dairy as a proxy and adjusted for this in models. Fourthly, our study did not account for changes in lipid concentrations over time, although this does not undermine the predictive value of a single measurement for fracture or osteoporosis risk. Fifthly, we did not include serum lipid concentrations measured at the time of the DXA scan due to insufficient participants with paired lipid measurements. However, the temporal sequence established by the chosen time points still provides valuable insights into the mediation effects. Sixthly, our study did not adjust for genetic confounding factors, such as polygenic risk scores, due to limited availability of comprehensive genetic data and partial capture of genetic influences by current GWAS. Future studies could incorporate genetic data to better understand the extent of genetic confounding and provide more robust estimates of the associations of interest. Lastly, due to the observational nature of the study, despite extensive control for potential confounders and risk factors, the possibility of residual confounding remains and causation cannot be established.

## 5 Conclusions

Our study reveals a complex interplay between different lipid profiles and skeletal health. Routine lipid profile assessments, including HDL‐C and Apo A among other lipid traits, may be integrated into the strategies for fracture risk stratification. Further research is needed to understand the underlying pathophysiological mechanisms behind these findings.

## Author Contributions

X.X, D.T.W.L, C.K.H.W, and F.K.H conceived the idea. X. X conducted the analysis and wrote the first draft. All authors contributed to the interpretation of the findings. All authors critically revised the abstract for intellectual content and approved the final version of the manuscript. The corresponding author attests that all listed authors meet authorship criteria and that no others meeting the criteria have been omitted.

## Ethics Statement

This study was performed under generic ethical approval obtained by UK Biobank from the National Health Service National Research Ethics Service (approval letter ref 11/NW/0382, 17 June 2011). All participants gave written informed consent before enrolment in the study, which was conducted in accordance with the principles of the Declaration of Helsinki.

## Conflicts of Interest

N.S has received grants and personal fees from Boehringer Ingelheim, and personal fees from Amgen, AstraZeneca, Eli Lilly, Merck Sharp & Dohme, Novartis, Novo Nordisk, Pfizer and Sanofi outside the submitted work. C.K.H.W reports receipt of research funding from the EuroQoL Group Research Foundation, the Hong Kong Research Grants Council, the Hong Kong Health and Medical Research Fund; AstraZeneca and Boehringer Ingelheim, unrelated to this work. All other authors declare no competing interests.

## Supporting information


**Table S1** Disease definition.
Table S2. Population attributable fraction of fracture and osteoporosis for quintiles of serum lipid concentrations.
Table S3. Associations between serum lipid concentrations and fracture risk, stratified by age.
Table S4. Associations between serum lipid concentrations and fracture risk, stratified by sex.
Table S5. Associations between serum lipid concentrations and fracture risk, stratified by BMI.
Table S6. Associations between serum lipid concentrations and fracture risk, stratified by CVD.
Table S7. Associations between serum lipid concentrations and fracture risk, stratified by use of lipid‐lowering medications.
Table S8. Associations between serum lipid traits and osteoporosis risk, stratified by age.
Table S9. Associations between serum lipid traits and osteoporosis risk, stratified by sex.
Table S10. Associations between serum lipid traits and osteoporosis risk, stratified by BMI.
Table S11. Associations between serum lipid traits and osteoporosis risk, stratified by CVD.
Table S12. Associations between serum lipid concentrations and osteoporosis risk, stratified by use of lipid‐lowering medications.
Table S13. Associations between serum lipid concentrations and the risk of type‐specific fractures in the UK Biobank.
Table 14. Associations between serum lipid concentrations and the risk of major osteoporotic fractures in the UK Biobank, stratified by age or sex.
Table S15. Associations between serum lipid concentrations and the risk of major osteoporotic fractures in the UK Biobank, stratified by BMI.
Table S16. Associations between serum lipid concentrations and the risk of major osteoporotic fractures in the UK Biobank, stratified by CVD or use of lipid‐lowering medications.
Table S17. Associations between serum lipid concentrations and the risk of hip fractures in the UK Biobank, stratified by age or sex.
Table S18. Associations between serum lipid concentrations and the risk of hip fractures in the UK Biobank, stratified by BMI.
Table S19. Associations between serum lipid concentrations and the risk of hip fractures in the UK Biobank, stratified by CVD or use of lipid‐lowering medications.
Table S20. Associations between serum lipid concentrations and the risk of clinical vertebral fractures in the UK Biobank, stratified by age or sex.
Table 21. Associations between serum lipid concentrations and the risk of clinical vertebral fractures in the UK Biobank, stratified by BMI.
Table 22. Associations between serum lipid concentrations and the risk of clinical vertebral fractures in the UK Biobank, stratified by CVD or use of lipid‐lowering medications.
**Figure S1.** The flow diagram for exclusion and inclusion.
**Figure S2.** Directed acyclic graph (DAG) of the mediation analysis
**Figure S3.** Associations between lipid traits and the risk of (A) fracture and (B) osteoporosis in minimally adjusted models.
**Figure S4.** Associations between lipid traits and fracture risk by age, sex, BMI, and cardiovascular disease, and use of lipid‐lowering medications
**Figure S5.** Associations between lipid traits and osteoporosis risk by age, sex, BMI, cardiovascular disease, and use of lipid‐lowering medications.

## Data Availability

UK Biobank data can be requested by bona fide researchers for approved projects, including replication, through https://www.ukbiobank.ac.uk/.
