# Supplementary material for: Associations of Serum Lipid Traits With Fracture and Osteoporosis: A Prospective Cohort Study From the UK Biobank
Source: J Cachexia Sarcopenia Muscle. 2024 Oct 29;15(6):2669–83. doi: 10.1002/jcsm.13611 (PMC11634517; doi:10.1002/jcsm.13611)
Supplement: Supplementary file 1 — Table S1 Disease definition. Table S2. Population attributable fraction of fracture and osteoporosis for quintiles of serum lipid concentrations. Table S3. Associations between serum lipid concentrations and fracture risk, stratified by age. Table S4. Associations between serum lipid concentrations and fracture risk, stratified by sex. Table S5. Associations between serum lipid concentrations and fracture risk, stratified by BMI. Table S6. Associations between serum lipid concentrations and fracture risk, stratified by CVD. Table S7. Associations between serum lipid concentrations and fracture risk, stratified by use of lipid‐lowering medications. Table S8. Associations between serum lipid traits and osteoporosis risk, stratified by age. Table S9. Associations between serum lipid traits and osteoporosis risk, stratified by sex. Table S10. Associations between serum lipid traits and osteoporosis risk, stratified by BMI. Table S11. Associations between serum lipid traits and osteoporosis risk, stratified by CVD. Table S12. Associations between serum lipid concentrations and osteoporosis risk, stratified by use of lipid‐lowering medications. Table S13. Associations between serum lipid concentrations and the risk of type‐specific fractures in the UK Biobank. Table 14. Associations between serum lipid concentrations and the risk of major osteoporotic fractures in the UK Biobank, stratified by age or sex. Table S15. Associations between serum lipid concentrations and the risk of major osteoporotic fractures in the UK Biobank, stratified by BMI. Table S16. Associations between serum lipid concentrations and the risk of major osteoporotic fractures in the UK Biobank, stratified by CVD or use of lipid‐lowering medications. Table S17. Associations between serum lipid concentrations and the risk of hip fractures in the UK Biobank, stratified by age or sex. Table S18. Associations between serum lipid concentrations and the risk of hip fractures in the UK Biobank, stratified by [file JCSM-15-2669-s001.docx]

# Supplemental Methods. The definitions of covariates, types of variables included in the models, and UK Biobank Data-Field IDs used in the study.

Age was calculated as the difference between the dates of birth and baseline assessment. Sex was self-reported as male or female, and ethnicity was self-classified and collapsed to White and non-white. Socioeconomic status was assessed using the Townsend area deprivation index, derived from the postcode of participants using data such as unemployment, car and home ownership, and household overcrowding.[1] Information on smoking status and alcohol intake were based on their self-reported data. Estimations of alcohol intake were derived from the self-reported frequency and volume of consumption. The International Physical Activity Questionnaire short form was used to collect physical activity data from participants' self-reports.[2] We calculated total physical activity by adding walking, moderate and vigorous activity, measured as metabolic equivalents (MET-min/week). A proxy for sedentary behavior (represented as hours per day) was created by summing the hours spent on sedentary activities, which was determined by asking, “how many hours do you spend during your leisure time watching TV, doing PC screening, or driving in a typical day?” Dietary habits, encompassing the intake of fruits and vegetables, red meat, processed meat, and oily fish, were evaluated using a baseline food frequency questionnaire. Dietary information from participants who reported ever eating eggs or dairy was used as a proxy for fat and calcium intake. Body mass index (BMI) was computed in accordance with the World Health Organization's criteria, categorizing BMI as underweight (<18.5 kg/m^2^), normal weight (18.5 to 24.9 kg/m^2^), overweight (25 to 29.9 kg/m^2^), and obese (≥30 kg/m^2^). Body fat percentage was measured by bio-impedance using standard equipment (Tanita BC418MA body composition analyser). Waist and hip circumferences were measured with a Wessex non-stretchable sprung tape. The waist-to-hip ratio was then calculated. All measurements were conducted following standardized protocols by trained nurses.

A morbidity count was derived from 43 long-term conditions, adapted for the UK Biobank from an extensive epidemiological study in Scotland.[3] Frailty status was determined using the modified Fried frailty phenotype, which assesses criteria such as weight loss, exhaustion, physical activity, walking speed, and grip strength. Participants were categorized as frail if they met three or more criteria and as prefrail if they met one or two criteria.[4] Anemia was defined by WHO criteria as hemoglobin concentrations below 130 g/L for men and below 120 g/L for women.[5] Positive rheumatoid factor was defined as levels above 50 IU/mL.[6] Vitamin D deficiency were defined as serum 25-hydroxyvitamin D levels less than 25 nmol/L.[7] Medical history and recent medication usage were collected via self-reported touchscreen questionnaires at baseline. All used UK Biobank Data-Field IDs and a clear description of how covariates were included in the model are provided in the eTable 1.

eTable 1. UK Biobank Data-Field IDs used in the study and types of variables included in the models.

| **Covariates** | **Data-Field** | **Types of Variables** |
| --- | --- | --- |
| Age | 21022 | Continuous |
| Ethnicity | 21000 | Categorical |
| Deprivation index | 189 | Continuous |
| Current smokers | 1239 | Categorical |
| Alcohol intake | 1558 | Continuous |
| Sleep duration | 1160 | Continuous |
| Total physical activity | 22037, 22038, 22039 | Continuous |
| Total sedentary behavior | 1070, 1080, 1090 | Continuous |
| Fruit and vegetables intake | 1309, 1319, 1289, 1299 | Continuous |
| Red meat intake | 1369, 1379, 1389 | Continuous |
| Processed meat intake | 1349 | Continuous |
| Oily fish intake | 1329 | Continuous |
| Ever eats eggs | 6144 | Categorical |
| Ever eats dairy | 6144 | Categorical |
| Body-mass index | 21001 | Categorical |
| Waist-hip ratio | 48, 49 | Continuous |
| Body fat percentage | 23099 | Continuous |
| Number of morbidities | 20001, 20002 | Categorical |
| Cardiovascular disease |  | Categorical |
| Hypertension |  | Categorical |
| Diabetes |  | Categorical |
| Chronic kidney disease |  | Categorical |
| Cancer |  | Categorical |
| Osteoporosis |  | Categorical |
| History of fractures |  | Categorical |
| Falls in the last year |  | Categorical |
| Positive rheumatoid factor | 30820 | Categorical |
| Vitamin D deficiency | 30890 | Categorical |
| Anemia | 30020 | Categorical |
| Prefrail/frail* | 2306, 2080, 22037, 22038, 22039, 924, 46, 47 | Categorical |
| Menopause | 2724 | Categorical |
| Lipid-lowering medications | 20003 | Categorical |
| Hormone therapy |  | Categorical |
| Aspirin |  | Categorical |
| Glucocorticoids |  |  |
| Vitamin D supplements |  | Categorical |
| Calcium supplements |  | Categorical |
| **Exposures** | **Data-Field** | **Types of Variables** |
| Apolipoprotein A | 30630 | Continuous or categorical |
| Apolipoprotein B | 30640 |  |
| Total cholesterol | 30690 |  |
| HDL cholesterol | 30760 |  |
| LDL cholesterol | 30780 |  |
| Triglycerides | 30870 |  |
| Lipoprotein A | 30790 |  |
| **Mediators** | **Data-Field** | **Types of Variables** |
| Right femoral neck BMD | 23208 | Continuous |
| Left femoral neck BMD | 23299 | Continuous |
| Lumbar spine BMD | 23204 | Continuous |

Notes: MET = Metabolic equivalent; HDL = High density lipoprotein; LDL = Low density lipoprotein; BMD = Bone mineral density.

**References:**

1. Townsend, P., P. Phillimore, and A. Beattie, *Health and deprivation: inequality and the North*. Vol. 8. 2023: Taylor & Francis.

2. Craig, C.L., et al., *International physical activity questionnaire: 12-country reliability and validity.* Med Sci Sports Exerc, 2003. **35**(8): p. 1381-95.

3. Barnett, K., et al., *Epidemiology of multimorbidity and implications for health care, research, and medical education: a cross-sectional study.* Lancet, 2012. **380**(9836): p. 37-43.

4. Petermann-Rocha, F., et al., *Associations between physical frailty and dementia incidence: a prospective study from UK Biobank.* Lancet Healthy Longev, 2020. **1**(2): p. e58-e68.

5. WHO, *Haemoglobin concentrations for the diagnosis of anaemia and assessment of severity*. 2011.

6. *Rheumatoid factor*. 2023 [cited 2024 28 July]; Available from: <https://www.ouh.nhs.uk/immunology/diagnostic-tests/tests-catalogue/rheumatoid-factor.aspx>.

7. *Vitamin D deficiency in adults*. 2022 [cited 2024 6 Aug]; Available from: <https://cks.nice.org.uk/topics/vitamin-d-deficiency-in-adults/#:~:text=Recommended%20vitamin%20D%20thresholds%20in,25%E2%80%9350%20nmol%2FL>.

# Supplementary Table 1. Disease definition.

| **ICD-10 Code for Fracture** | **Description** |
| --- | --- |
| Skull, and facial bones | |
| S02.0 | Fracture of vault of skull |
| S02.1 | Fracture of base of skull |
| S02.2 | Fracture of nasal bones |
| S02.3 | Fracture of orbital floor |
| S02.4 | Fracture of malar and maxillary bones |
| S02.6 | Fracture of mandible |
| S02.7 | Multiple fractures involving skull and facial bones |
| S02.8 | Fractures of other skull and facial bones |
| S02.9 | Fracture of skull and facial bones, part unspecified |
| Neck | |
| S12.0 | Fracture of first cervical vertebra |
| S12.1 | Fracture of second cervical vertebra |
| S12.2 | Fracture of other specified cervical vertebra |
| S12.7 | Multiple fractures of cervical spine |
| S12.8 | Fracture of other parts of neck |
| S12.9 | Fracture of neck, part unspecified |
| Rib(s), and sternum | |
| S22.0 | Fracture of thoracic vertebra |
| S22.1 | Multiple fractures of thoracic spine |
| S22.2 | Fracture of sternum |
| S22.3 | Fracture of rib |
| S22.4 | Multiple fractures of ribs |
| S22.5 | Flail chest |
| S22.8 | Fracture of other parts of bony thorax |
| S22.9 | Fracture of bony thorax, part unspecified |
| Pelvis | |
| S32.0 | Fracture of lumbar vertebra |
| S32.1 | Fracture of sacrum |
| S32.2 | Fracture of coccyx |
| S32.3 | Fracture of ilium |
| S32.4 | Fracture of acetabulum |
| S32.5 | Fracture of pubis |
| S32.7 | Multiple fractures of lumbar spine and pelvis |
| S32.8 | Fracture of other and unspecified parts of lumbar spine and pelvis |
| Shoulder and upper arm | |
| S42.0 | Fracture of clavicle |
| S42.1 | Fracture of scapula |
| S42.2 | Fracture of upper end of humerus |
| S42.3 | Fracture of shaft of humerus |
| S42.4 | Fracture of lower end of humerus |
| S42.7 | Multiple fractures of clavicle, scapula and humerus |
| S42.8 | Fracture of other parts of shoulder and upper arm |
| S42.9 | Fracture of shoulder girdle, part unspecified |
| Forearm | |
| S52.0 | Fracture of upper end of ulna |
| S52.1 | Fracture of upper end of radius |
| S52.2 | Fracture of shaft of ulna |
| S52.3 | Fracture of shaft of radius |
| S52.4 | Fracture of shafts of both ulna and radius |
| S52.5 | Fracture of lower end of radius |
| S52.6 | Fracture of lower end of both ulna and radius |
| S52.7 | Multiple fractures of forearm |
| S52.8 | Fracture of other parts of forearm |
| S52.9 | Fracture of forearm, part unspecified |
| Hand, and fingers | |
| S62.0 | Fracture of navicular [scaphoid] bone of hand |
| S62.1 | Fracture of other carpal bone(s) |
| S62.2 | Fracture of first metacarpal bone |
| S62.3 | Fracture of other metacarpal bone |
| S62.4 | Multiple fractures of metacarpal bones |
| S62.5 | Fracture of thumb |
| S62.6 | Fracture of other finger |
| S62.7 | Multiple fractures of fingers |
| S62.8 | Fracture of other and unspecified parts of wrist and hand |
| Femur | |
| S72.0 | Fracture of neck of femur |
| S72.1 | Pertrochanteric fracture |
| S72.2 | Subtrochanteric fracture |
| S72.3 | Fracture of shaft of femur |
| S72.4 | Fracture of lower end of femur |
| S72.7 | Multiple fractures of femur |
| S72.8 | Fractures of other parts of femur |
| S72.9 | Fracture of femur, part unspecified |
| Lower leg | |
| S82.0 | Fracture of patella |
| S82.1 | Fracture of upper end of tibia |
| S82.2 | Fracture of shaft of tibia |
| S82.3 | Fracture of lower end of tibia |
| S82.4 | Fracture of fibula alone |
| S82.5 | Fracture of medial malleolus |
| S82.6 | Fracture of lateral malleolus |
| S82.7 | Multiple fractures of lower leg |
| S82.8 | Fractures of other parts of lower leg |
| S82.9 | Fracture of lower leg, part unspecified |
| Foot, and toes | |
| S92.0 | Fracture of calcaneus |
| S92.1 | Fracture of talus |
| S92.2 | Fracture of other tarsal bone(s) |
| S92.3 | Fracture of metatarsal bone |
| S92.4 | Fracture of great toe |
| S92.5 | Fracture of other toe |
| S92.7 | Multiple fractures of foot |
| S92.9 | Fracture of foot, unspecified |
| Fractures involving multiple body regions | |
| T02.0 | Fractures involving head with neck |
| T02.1 | Fractures involving thorax with lower back and pelvis |
| T02.2 | Fractures involving multiple regions of one upper limb |
| T02.3 | Fractures involving multiple regions of one lower limb |
| T02.4 | Fractures involving multiple regions of both upper limbs |
| T02.5 | Fractures involving multiple regions of both lower limbs |
| T02.6 | Fractures involving multiple regions of upper limb(s) with lower limb(s) |
| T02.7 | Fractures involving thorax with lower back and pelvis with limb(s) |
| T02.8 | Fractures involving other combinations of body regions |
| T02.9 | Multiple fractures, unspecified |
| Others | |
| M48.4 | Fatigue fracture of vertebra |
| M48.5 | Collapsed vertebra, not elsewhere classified |
| M49.5 | Collapsed vertebra in diseases classified elsewhere |
| M80 | Osteoporosis with pathological fracture |
| M80.0 | Postmenopausal osteoporosis with pathological fracture |
| M80.1 | Postoophorectomy osteoporosis with pathological fracture |
| M80.2 | Osteoporosis of disuse with pathological fracture |
| M80.3 | Postsurgical malabsorption osteoporosis with pathological fracture |
| M80.4 | Drug-induced osteoporosis with pathological fracture |
| M80.5 | Idiopathic osteoporosis with pathological fracture |
| M80.8 | Other osteoporosis with pathological fracture |
| M80.9 | Unspecified osteoporosis with pathological fracture |
| M96.6 | Fracture of bone following insertion of orthopaedic implant, joint prosthesis, or bone plate |
| T08 | Fracture of spine, level unspecified |
| T10 | Fracture of upper limb, level unspecified |
| T12 | Fracture of lower limb, level unspecified |
| T14.2 | Fracture of unspecified body region |
| X59.0 | Exposure to unspecified factor causing fracture |
| **ICD-10 Code for Osteoporosis** | **Description** |
| Osteoporosis with pathological fracture | |
| M80 | Osteoporosis with pathological fracture |
| M80.0 | Postmenopausal osteoporosis with pathological fracture |
| M80.1 | Postoophorectomy osteoporosis with pathological fracture |
| M80.2 | Osteoporosis of disuse with pathological fracture |
| M80.3 | Postsurgical malabsorption osteoporosis with pathological fracture |
| M80.4 | Drug-induced osteoporosis with pathological fracture |
| M80.5 | Idiopathic osteoporosis with pathological fracture |
| M80.8 | Other osteoporosis with pathological fracture |
| M80.9 | Unspecified osteoporosis with pathological fracture |
| Osteoporosis without pathological fracture | |
| M81.0 | Postmenopausal osteoporosis |
| M81.1 | Postoophorectomy osteoporosis |
| M81.2 | Osteoporosis of disuse |
| M81.3 | Postsurgical malabsorption osteoporosis |
| M81.4 | Drug-induced osteoporosis |
| M81.5 | Idiopathic osteoporosis |
| M81.6 | Localized osteoporosis [Lequesne] |
| M81.8 | Other osteoporosis |
| M81.9 | Osteoporosis, unspecified |
| Osteoporosis in diseases classified elsewhere | |
| M82.0 | Osteoporosis in multiple myelomatosis |
| M82.1 | Osteoporosis in endocrine disorders |
| M82.8 | Osteoporosis in other diseases classified elsewhere |
| **ICD-10 Code for Major Osteoporotic Fracture** | **Description** |
| S12.0 | Fracture of first cervical vertebra |
| S12.1 | Fracture of second cervical vertebra |
| S12.2 | Fracture of other specified cervical vertebra |
| S12.7 | Multiple fractures of cervical spine |
| S12.8 | Fracture of other parts of neck |
| S12.9 | Fracture of neck, part unspecified |
| S22.0 | Fracture of thoracic vertebra |
| S22.1 | Multiple fractures of thoracic spine |
| S32.0 | Fracture of lumbar vertebra |
| S32.7 | Multiple fractures of lumbar spine and pelvis |
| S32.8 | Fracture of other and unspecified parts of lumbar spine and pelvis |
| S42.2 | Fracture of upper end of humerus |
| S52.5 | Fracture of lower end of radius |
| S52.6 | Fracture of lower end of both ulna and radius |
| S62.0 | Fracture of navicular [scaphoid] bone of hand |
| S62.1 | Fracture of other carpal bone(s) |
| S62.8 | Fracture of other and unspecified parts of wrist and hand |
| S72.0 | Fracture of neck of femur |
| S72.1 | Pertrochanteric fracture |
| S72.2 | Subtrochanteric fracture |
| M48.4 | Fatigue fracture of vertebra |
| M48.5 | Collapsed vertebra, not elsewhere classified |
| M49.5 | Collapsed vertebra in diseases classified elsewhere |
| T02.0 | Fractures involving head with neck |
| T02.1 | Fractures involving thorax with lower back and pelvis |
| T02.7 | Fractures involving thorax with lower back and pelvis with limb(s) |
| T08 | Fracture of spine, level unspecified |
| **ICD-10 Code for Hip Fractures** | **Description** |
| S72.0 | Fracture of neck of femur |
| S72.1 | Pertrochanteric fracture |
| S72.2 | Subtrochanteric fracture |
| **ICD-10 Code for Vertebral Fracture** | **Description** |
| M48.4 | Fatigue fracture of vertebra |
| M48.5 | Collapsed vertebra, not elsewhere classified |
| M49.5 | Collapsed vertebra in diseases classified elsewhere |
| S12.0 | Fracture of first cervical vertebra |
| S12.1 | Fracture of second cervical vertebra |
| S12.2 | Fracture of other specified cervical vertebra |
| S12.7 | Multiple fractures of cervical spine |
| S12.8 | Fracture of other parts of neck |
| S12.9 | Fracture of neck, part unspecified |
| S22.0 | Fracture of thoracic vertebra |
| S22.1 | Multiple fractures of thoracic spine |
| S32.0 | Fracture of lumbar vertebra |
| T08 | Fracture of spine, level unspecified |

# Supplementary Table 2. Population attributable fraction of fracture and osteoporosis for quintiles of serum lipid concentrations.

| **Lipid traits** | **Quintile 2-5 vs Quintile 1** | | | |
| --- | --- | --- | --- | --- |
|  | **Fracture** | | **Osteoporosis** | |
|  | **PAF** | **(95% CI)** | **PAF** | **(95% CI)** |
| **Apolipoprotein A** | 7.31% | (4.05%, 10.45%) | 5.11% | (-2.73%, 12.34%) |
| **Apolipoprotein B** | -9.99% | (-13.20%, -6.86%) | -8.98% | (-15.32%, -2.99%) |
| **Total cholesterol** | -2.72% | (-6.10%, 0.54%) | -1.55% | (-8.81%, 5.24%) |
| **HDL cholesterol** | 11.65% | (8.45%, 14.73%) | 14.05% | (6.44%, 21.04%) |
| **LDL cholesterol** | -6.76% | (-10.11%, -3.51%) | -4.69% | (-11.35%, 1.57%) |
| **Triglycerides** | -14.76% | (-18.09%, -11.53%) | -13.28% | (-19.24%, -7.63%) |
| **Lipoprotein A** | 0.06% | (-2.72%, 2.76%) | -2.35% | (-7.91%, 2.93%) |

Notes: PAF = Population attributable fraction; CI = Confidence interval. Model was adjusted for age, sex, ethnicity, deprivation index, current smokers, alcohol intake, sleep duration, total physical activity, total sedentary behavior, fruit and vegetables intake, red meat intake, processed meat intake, oily fish intake, ever eats eggs, ever eats dairy, body-mass index, waist-hip ratio, body fat percentage, number of morbidities, prefrail/frail status, cardiovascular disease, hypertension, diabetes , chronic kidney disease, cancer, anemia, positive rheumatoid factor, vitamin D deficiency, osteoporosis (excluded in the osteoporosis model), history of fractures (excluded in the fracture model), falls in the last year, lipid-lowering medications, aspirin, glucocorticoids, vitamin D supplements, and calcium supplements.

# Supplementary Table 3. Associations between serum lipid concentrations and fracture risk, stratified by age.

| **Lipid traits** | **Age ≥ 60 years (ref)** | | | | **Age < 60 years** | | | | **Ratio of HR  (95% CI)** | **P-value** |
| --- | --- | --- | --- | --- | --- | --- | --- | --- | --- | --- |
|  | **Event** | **Crude incidence rate** | **HR (95% CI)** | **P-value** | **Event** | **Crude incidence rate** | **HR (95% CI)** | **P-value** |  |  |
| **Apolipoprotein A** |  |  |  |  |  |  |  |  |  |  |
| Quintile 1 | 2340 | 5.74 | Reference | | 2085 | 3.21 | Reference | | Reference | |
| Quintile 2 | 2429 | 5.74 | 0.96 (0.89, 1.02) | 0.174 | 2089 | 3.40 | 1.06 (0.99, 1.14) | 0.108 | 1.04 (0.95, 1.15) | 0.391 |
| Quintile 3 | 2820 | 6.44 | 1.02 (0.95, 1.09) | 0.596 | 2302 | 3.88 | 1.22 (1.14, 1.31) | <0.001 | 1.06 (0.97, 1.16) | 0.194 |
| Quintile 4 | 3214 | 7.19 | 1.07 (1.00, 1.14) | 0.037 | 2369 | 4.14 | 1.26 (1.18, 1.36) | <0.001 | 0.99 (0.91, 1.09) | 0.858 |
| Quintile 5 | 3785 | 8.12 | 1.09 (1.02, 1.17) | 0.009 | 2485 | 4.63 | 1.36 (1.26, 1.46) | <0.001 | 0.99 (0.91, 1.08) | 0.853 |
| Per 1-SD increase | NA | NA | 1.05 (1.03, 1.07) | <0.001 | NA | NA | 1.12 (1.09, 1.14) | <0.001 | 0.99 (0.97, 1.02) | 0.538 |
| **Apolipoprotein B** |  |  |  |  |  |  |  |  |  |  |
| Quintile 1 | 3272 | 7.42 | Reference | | 2379 | 4.03 | Reference | | Reference | |
| Quintile 2 | 2838 | 6.82 | 0.91 (0.86, 0.96) | <0.001 | 2389 | 3.86 | 0.96 (0.90, 1.02) | 0.210 | 1.03 (0.94, 1.12) | 0.560 |
| Quintile 3 | 2751 | 6.45 | 0.87 (0.82, 0.93) | <0.001 | 2314 | 3.84 | 1.00 (0.94, 1.07) | 0.892 | 1.10 (1.01, 1.20) | 0.024 |
| Quintile 4 | 2857 | 6.49 | 0.86 (0.81, 0.92) | <0.001 | 2161 | 3.68 | 0.97 (0.91, 1.04) | 0.387 | 1.08 (0.99, 1.18) | 0.091 |
| Quintile 5 | 2870 | 6.25 | 0.83 (0.78, 0.89) | <0.001 | 2087 | 3.68 | 0.95 (0.89, 1.02) | 0.174 | 1.10 (1.01, 1.20) | 0.036 |
| Per 1-SD increase | NA | NA | 0.94 (0.92, 0.96) | <0.001 | NA | NA | 0.99 (0.97, 1.01) | 0.355 | 1.04 (1.01, 1.06) | 0.016 |
| **Total cholesterol** |  |  |  |  |  |  |  |  |  |  |
| Quintile 1 | 3228 | 6.73 | Reference | | 2094 | 3.76 | Reference | | Reference | |
| Quintile 2 | 2636 | 6.70 | 0.98 (0.92, 1.04) | 0.515 | 2364 | 3.68 | 1.02 (0.96, 1.10) | 0.477 | 0.96 (0.88, 1.05) | 0.411 |
| Quintile 3 | 2627 | 6.53 | 0.94 (0.88, 1.00) | 0.061 | 2345 | 3.73 | 1.07 (0.99, 1.14) | 0.072 | 1.02 (0.93, 1.11) | 0.715 |
| Quintile 4 | 2882 | 6.70 | 0.94 (0.88, 1.01) | 0.082 | 2326 | 3.90 | 1.13 (1.05, 1.21) | <0.001 | 1.05 (0.96, 1.15) | 0.302 |
| Quintile 5 | 3215 | 6.75 | 0.90 (0.84, 0.96) | 0.002 | 2201 | 4.07 | 1.12 (1.04, 1.20) | 0.002 | 1.07 (0.98, 1.17) | 0.117 |
| Per 1-SD increase | NA | NA | 0.96 (0.94, 0.98) | <0.001 | NA | NA | 1.04 (1.01, 1.06) | 0.002 | 1.03 (1.00, 1.06) | 0.072 |
| **HDL cholesterol** |  |  |  |  |  |  |  |  |  |  |
| Quintile 1 | 2442 | 5.49 | Reference | | 1952 | 3.21 | Reference | | Reference | |
| Quintile 2 | 2558 | 5.83 | 1.03 (0.97, 1.10) | 0.374 | 2075 | 3.44 | 1.10 (1.02, 1.18) | 0.012 | 0.99 (0.90, 1.08) | 0.778 |
| Quintile 3 | 2800 | 6.46 | 1.07 (1.00, 1.14) | 0.038 | 2256 | 3.80 | 1.23 (1.15, 1.33) | <0.001 | 1.00 (0.91, 1.10) | 0.950 |
| Quintile 4 | 3130 | 7.30 | 1.15 (1.08, 1.23) | <0.001 | 2426 | 4.09 | 1.31 (1.21, 1.41) | <0.001 | 0.93 (0.85, 1.02) | 0.140 |
| Quintile 5 | 3658 | 8.38 | 1.24 (1.16, 1.33) | <0.001 | 2621 | 4.61 | 1.43 (1.32, 1.55) | <0.001 | 0.90 (0.82, 0.98) | 0.015 |
| Per 1-SD increase | NA | NA | 1.08 (1.06, 1.11) | <0.001 | NA | NA | 1.14 (1.11, 1.17) | <0.001 | 0.97 (0.95, 1.00) | 0.025 |
| **LDL cholesterol** |  |  |  |  |  |  |  |  |  |  |
| Quintile 1 | 3394 | 7.11 | Reference | | 2208 | 3.99 | Reference | | Reference | |
| Quintile 2 | 2746 | 6.86 | 0.94 (0.88, 1.00) | 0.042 | 2410 | 3.81 | 1.00 (0.93, 1.07) | 0.955 | 1.00 (0.91, 1.09) | 0.952 |
| Quintile 3 | 2672 | 6.51 | 0.91 (0.85, 0.97) | 0.004 | 2341 | 3.77 | 1.02 (0.96, 1.09) | 0.512 | 1.04 (0.95, 1.13) | 0.426 |
| Quintile 4 | 2781 | 6.46 | 0.88 (0.83, 0.94) | <0.001 | 2241 | 3.74 | 1.04 (0.97, 1.11) | 0.303 | 1.08 (0.98, 1.17) | 0.106 |
| Quintile 5 | 2995 | 6.46 | 0.87 (0.81, 0.92) | <0.001 | 2130 | 3.81 | 1.02 (0.95, 1.10) | 0.501 | 1.07 (0.98, 1.17) | 0.130 |
| Per 1-SD increase | NA | NA | 0.95 (0.93, 0.97) | <0.001 | NA | NA | 1.01 (0.98, 1.03) | 0.597 | 1.03 (1.00, 1.06) | 0.060 |
| **Triglycerides** |  |  |  |  |  |  |  |  |  |  |
| Quintile 1 | 2602 | 8.07 | Reference | | 2823 | 3.97 | Reference | | Reference | |
| Quintile 2 | 3090 | 7.24 | 0.89 (0.84, 0.94) | <0.001 | 2302 | 3.85 | 0.96 (0.90, 1.02) | 0.145 | 1.09 (1.00, 1.18) | 0.052 |
| Quintile 3 | 3120 | 6.61 | 0.82 (0.78, 0.87) | <0.001 | 2162 | 3.90 | 0.99 (0.93, 1.06) | 0.802 | 1.24 (1.14, 1.35) | <0.001 |
| Quintile 4 | 3064 | 6.19 | 0.78 (0.73, 0.83) | <0.001 | 1986 | 3.71 | 0.91 (0.85, 0.98) | 0.011 | 1.24 (1.14, 1.35) | <0.001 |
| Quintile 5 | 2712 | 5.82 | 0.73 (0.69, 0.78) | <0.001 | 2057 | 3.62 | 0.89 (0.83, 0.96) | 0.002 | 1.34 (1.23, 1.46) | <0.001 |
| Per 1-SD increase | NA | NA | 0.90 (0.88, 0.92) | <0.001 | NA | NA | 0.96 (0.94, 0.99) | 0.002 | 1.11 (1.08, 1.14) | <0.001 |
| **Lipoprotein A** |  |  |  |  |  |  |  |  |  |  |
| Quintile 1 | 2827 | 6.64 | Reference | | 2312 | 3.80 | Reference | | Reference | |
| Quintile 2 | 2747 | 6.57 | 0.98 (0.93, 1.04) | 0.531 | 2399 | 3.92 | 1.07 (1.00, 1.14) | 0.049 | 1.07 (0.98, 1.17) | 0.120 |
| Quintile 3 | 2906 | 6.49 | 0.95 (0.90, 1.01) | 0.090 | 2239 | 3.86 | 1.04 (0.98, 1.11) | 0.193 | 1.07 (0.98, 1.16) | 0.144 |
| Quintile 4 | 3030 | 6.90 | 1.01 (0.95, 1.07) | 0.775 | 2202 | 3.73 | 1.03 (0.97, 1.10) | 0.318 | 0.99 (0.91, 1.08) | 0.808 |
| Quintile 5 | 3078 | 6.82 | 0.97 (0.92, 1.03) | 0.350 | 2178 | 3.79 | 1.03 (0.96, 1.10) | 0.382 | 1.03 (0.95, 1.12) | 0.482 |
| Per 1-SD increase | NA | NA | 1.00 (0.98, 1.02) | 0.803 | NA | NA | 1.00 (0.98, 1.02) | 0.973 | 1.00 (0.97, 1.02) | 0.829 |

Notes: HR = Hazard ratio; CI = Confidence interval; HDL = High density lipoprotein; LDL = Low density lipoprotein; NA= Not available. The unit of crude incidence rate: events per 1,000 person-years. Model was adjusted for sex, ethnicity, deprivation index, current smokers, alcohol intake, sleep duration, total physical activity, total sedentary behavior, fruit and vegetables intake, red meat intake, processed meat intake, oily fish intake, ever eats eggs, ever eats dairy, body-mass index, waist-hip ratio, body fat percentage, number of morbidities, prefrail/frail status, cardiovascular disease, hypertension, diabetes , chronic kidney disease, cancer, anemia, positive rheumatoid factor, vitamin D deficiency, osteoporosis, falls in the last year, lipid-lowering medications, aspirin, glucocorticoids, vitamin D supplements, and calcium supplements.

# Supplementary Table 4. Associations between serum lipid concentrations and fracture risk, stratified by sex.

| **Lipid traits** | **Women (ref)** | | | | **Men** | | | | **Ratio of HR  (95% CI)** | **P-value** |
| --- | --- | --- | --- | --- | --- | --- | --- | --- | --- | --- |
|  | **Event** | **Crude incidence rate** | **HR (95% CI)** | **P-value** | **Event** | **Crude incidence rate** | **HR (95% CI)** | **P-value** |  |  |
| **Apolipoprotein A** |  |  |  |  |  |  |  |  |  |  |
| Quintile 1 | 1441 | 5.35 | Reference | | 2984 | 3.79 | Reference | | Reference | |
| Quintile 2 | 2163 | 5.20 | 0.94 (0.87, 1.01) | 0.099 | 2355 | 3.79 | 1.03 (0.97, 1.09) | 0.400 | 1.04 (0.95, 1.15) | 0.384 |
| Quintile 3 | 3120 | 5.64 | 1.00 (0.93, 1.07) | 0.967 | 2002 | 4.19 | 1.15 (1.08, 1.23) | <0.001 | 1.06 (0.97, 1.17) | 0.203 |
| Quintile 4 | 4046 | 5.91 | 1.02 (0.95, 1.09) | 0.654 | 1537 | 4.59 | 1.21 (1.13, 1.30) | <0.001 | 1.08 (0.98, 1.19) | 0.141 |
| Quintile 5 | 5137 | 6.39 | 1.02 (0.94, 1.09) | 0.678 | 1133 | 5.69 | 1.37 (1.26, 1.48) | <0.001 | 1.17 (1.06, 1.29) | 0.003 |
| Per 1-SD increase | NA | NA | 1.02 (1.00, 1.04) | 0.026 | NA | NA | 1.13 (1.10, 1.16) | <0.001 | 1.05 (1.02, 1.09) | <0.001 |
| **Apolipoprotein B** |  |  |  |  |  |  |  |  |  |  |
| Quintile 1 | 3149 | 5.96 | Reference | | 2502 | 4.98 | Reference | | Reference | |
| Quintile 2 | 3185 | 5.69 | 0.92 (0.87, 0.97) | 0.003 | 2042 | 4.30 | 0.92 (0.86, 0.98) | 0.014 | 0.99 (0.91, 1.08) | 0.894 |
| Quintile 3 | 3190 | 5.79 | 0.92 (0.87, 0.97) | 0.003 | 1875 | 3.93 | 0.90 (0.84, 0.96) | 0.003 | 0.95 (0.87, 1.04) | 0.282 |
| Quintile 4 | 3189 | 5.89 | 0.88 (0.83, 0.93) | <0.001 | 1829 | 3.77 | 0.88 (0.82, 0.95) | <0.001 | 0.96 (0.88, 1.05) | 0.376 |
| Quintile 5 | 3194 | 5.85 | 0.83 (0.78, 0.89) | <0.001 | 1763 | 3.67 | 0.88 (0.82, 0.95) | <0.001 | 1.00 (0.92, 1.09) | 0.992 |
| Per 1-SD increase | NA | NA | 0.94 (0.92, 0.96) | <0.001 | NA | NA | 0.96 (0.94, 0.99) | 0.002 | 1.00 (0.97, 1.03) | 0.894 |
| **Total cholesterol** |  |  |  |  |  |  |  |  |  |  |
| Quintile 1 | 2396 | 5.73 | Reference | | 2926 | 4.72 | Reference | | Reference | |
| Quintile 2 | 2906 | 5.54 | 1.00 (0.94, 1.06) | 0.957 | 2094 | 4.10 | 0.97 (0.91, 1.04) | 0.443 | 0.99 (0.90, 1.08) | 0.791 |
| Quintile 3 | 3096 | 5.62 | 0.98 (0.91, 1.04) | 0.453 | 1876 | 3.91 | 0.96 (0.89, 1.03) | 0.237 | 0.97 (0.89, 1.06) | 0.512 |
| Quintile 4 | 3525 | 6.03 | 0.98 (0.92, 1.05) | 0.648 | 1683 | 3.81 | 0.98 (0.91, 1.06) | 0.598 | 0.96 (0.88, 1.05) | 0.366 |
| Quintile 5 | 3984 | 6.14 | 0.92 (0.87, 0.99) | 0.019 | 1432 | 3.89 | 0.98 (0.91, 1.06) | 0.671 | 1.00 (0.91, 1.10) | 0.999 |
| Per 1-SD increase | NA | NA | 0.96 (0.94, 0.98) | <0.001 | NA | NA | 1.00 (0.97, 1.02) | 0.881 | 1.01 (0.98, 1.04) | 0.519 |
| **HDL cholesterol** |  |  |  |  |  |  |  |  |  |  |
| Quintile 1 | 1305 | 5.55 | Reference | | 3089 | 3.78 | Reference | | Reference | |
| Quintile 2 | 2222 | 5.42 | 0.99 (0.91, 1.07) | 0.732 | 2411 | 3.82 | 1.07 (1.01, 1.14) | 0.029 | 1.05 (0.95, 1.16) | 0.362 |
| Quintile 3 | 3060 | 5.50 | 1.00 (0.93, 1.08) | 0.955 | 1996 | 4.25 | 1.20 (1.12, 1.28) | <0.001 | 1.13 (1.02, 1.24) | 0.016 |
| Quintile 4 | 4080 | 5.83 | 1.05 (0.98, 1.14) | 0.173 | 1476 | 4.58 | 1.27 (1.18, 1.37) | <0.001 | 1.12 (1.02, 1.24) | 0.021 |
| Quintile 5 | 5240 | 6.35 | 1.10 (1.02, 1.19) | 0.016 | 1039 | 5.82 | 1.49 (1.37, 1.63) | <0.001 | 1.22 (1.10, 1.36) | <0.001 |
| Per 1-SD increase | NA | NA | 1.05 (1.03, 1.08) | <0.001 | NA | NA | 1.16 (1.13, 1.19) | <0.001 | 1.06 (1.03, 1.09) | <0.001 |
| **LDL cholesterol** |  |  |  |  |  |  |  |  |  |  |
| Quintile 1 | 2905 | 6.01 | Reference | | 2697 | 4.93 | Reference | | Reference | |
| Quintile 2 | 3097 | 5.61 | 0.95 (0.89, 1.01) | 0.079 | 2059 | 4.28 | 0.96 (0.90, 1.02) | 0.209 | 1.03 (0.94, 1.12) | 0.544 |
| Quintile 3 | 3149 | 5.72 | 0.95 (0.90, 1.01) | 0.110 | 1864 | 3.87 | 0.91 (0.85, 0.98) | 0.010 | 0.95 (0.88, 1.04) | 0.299 |
| Quintile 4 | 3229 | 5.87 | 0.92 (0.86, 0.97) | 0.006 | 1793 | 3.73 | 0.92 (0.85, 0.99) | 0.028 | 0.98 (0.90, 1.08) | 0.735 |
| Quintile 5 | 3527 | 5.96 | 0.88 (0.82, 0.93) | <0.001 | 1598 | 3.71 | 0.92 (0.85, 1.00) | 0.041 | 1.01 (0.92, 1.10) | 0.843 |
| Per 1-SD increase | NA | NA | 0.95 (0.93, 0.97) | <0.001 | NA | NA | 0.97 (0.94, 0.99) | 0.017 | 1.00 (0.97, 1.03) | 0.890 |
| **Triglycerides** |  |  |  |  |  |  |  |  |  |  |
| Quintile 1 | 3812 | 5.49 | Reference | | 1613 | 4.77 | Reference | | Reference | |
| Quintile 2 | 3605 | 5.85 | 0.90 (0.86, 0.95) | <0.001 | 1787 | 4.38 | 0.92 (0.85, 0.99) | 0.022 | 0.94 (0.86, 1.03) | 0.194 |
| Quintile 3 | 3342 | 6.02 | 0.87 (0.82, 0.92) | <0.001 | 1940 | 4.11 | 0.88 (0.82, 0.95) | <0.001 | 0.91 (0.84, 1.00) | 0.048 |
| Quintile 4 | 2893 | 5.95 | 0.80 (0.75, 0.85) | <0.001 | 2157 | 3.97 | 0.84 (0.78, 0.90) | <0.001 | 0.93 (0.85, 1.02) | 0.107 |
| Quintile 5 | 2255 | 6.01 | 0.77 (0.72, 0.82) | <0.001 | 2514 | 3.81 | 0.81 (0.75, 0.87) | <0.001 | 0.94 (0.86, 1.03) | 0.166 |
| Per 1-SD increase | NA | NA | 0.91 (0.88, 0.93) | <0.001 | NA | NA | 0.95 (0.93, 0.97) | <0.001 | 1.01 (0.98, 1.05) | 0.365 |
| **Lipoprotein A** |  |  |  |  |  |  |  |  |  |  |
| Quintile 1 | 2931 | 5.78 | Reference | | 2208 | 4.18 | Reference | | Reference | |
| Quintile 2 | 3041 | 5.82 | 1.03 (0.97, 1.09) | 0.350 | 2105 | 4.14 | 1.00 (0.94, 1.07) | 0.993 | 0.98 (0.89, 1.06) | 0.584 |
| Quintile 3 | 3235 | 5.81 | 0.98 (0.93, 1.04) | 0.549 | 1910 | 4.06 | 0.98 (0.92, 1.05) | 0.571 | 0.98 (0.90, 1.07) | 0.684 |
| Quintile 4 | 3357 | 5.89 | 1.01 (0.95, 1.07) | 0.780 | 1875 | 4.08 | 1.02 (0.95, 1.09) | 0.625 | 1.00 (0.92, 1.10) | 0.928 |
| Quintile 5 | 3343 | 5.85 | 0.98 (0.92, 1.03) | 0.392 | 1913 | 4.21 | 1.01 (0.95, 1.08) | 0.734 | 1.02 (0.93, 1.11) | 0.695 |
| Per 1-SD increase | NA | NA | 0.99 (0.97, 1.01) | 0.238 | NA | NA | 1.01 (0.99, 1.03) | 0.478 | 1.01 (0.98, 1.04) | 0.409 |

Notes: HR = Hazard ratio; CI = Confidence interval; HDL = High density lipoprotein; LDL = Low density lipoprotein; NA= Not available. The unit of crude incidence rate: events per 1,000 person-years. Model was adjusted for age, ethnicity, deprivation index, current smokers, alcohol intake, sleep duration, total physical activity, total sedentary behavior, fruit and vegetables intake, red meat intake, processed meat intake, oily fish intake, ever eats eggs, ever eats dairy, body-mass index, waist-hip ratio, body fat percentage, number of morbidities, prefrail/frail status, cardiovascular disease, hypertension, diabetes , chronic kidney disease, cancer, anemia, positive rheumatoid factor, vitamin D deficiency, osteoporosis, falls in the last year, menopause status (included in the model for women), lipid-lowering medications, aspirin, glucocorticoids, vitamin D supplements, and calcium supplements.

# Supplementary Table 5. Associations between serum lipid concentrations and fracture risk, stratified by BMI.

| **Lipid traits** | **Normal weight (ref)** | | | | **Overweight** | | | | **Ratio of HR  (95% CI)** | **P-value** |
| --- | --- | --- | --- | --- | --- | --- | --- | --- | --- | --- |
|  | **Event** | **Crude incidence rate** | **HR (95% CI)** | **P-value** | **Event** | **Crude incidence rate** | **HR (95% CI)** | **P-value** |  |  |
| **Apolipoprotein A** |  |  |  |  |  |  |  |  |  |  |
| Quintile 1 | 811 | 4.38 | Reference | | 1848 | 3.86 | Reference | | Reference | |
| Quintile 2 | 1111 | 4.45 | 0.97 (0.87, 1.07) | 0.547 | 1967 | 4.11 | 1.01 (0.94, 1.08) | 0.821 | 1.03 (0.91, 1.17) | 0.627 |
| Quintile 3 | 1635 | 5.06 | 1.09 (0.99, 1.20) | 0.092 | 2180 | 4.79 | 1.11 (1.04, 1.20) | 0.004 | 1.01 (0.90, 1.14) | 0.804 |
| Quintile 4 | 2178 | 5.44 | 1.08 (0.98, 1.18) | 0.131 | 2225 | 5.29 | 1.17 (1.09, 1.26) | <0.001 | 1.08 (0.96, 1.21) | 0.188 |
| Quintile 5 | 3165 | 6.27 | 1.10 (1.00, 1.21) | 0.056 | 2192 | 6.09 | 1.18 (1.09, 1.28) | <0.001 | 1.06 (0.95, 1.18) | 0.322 |
| Per 1-SD increase | NA | NA | 1.04 (1.01, 1.06) | 0.003 | NA | NA | 1.06 (1.03, 1.09) | <0.001 | 1.02 (0.99, 1.05) | 0.202 |
| **Apolipoprotein B** |  |  |  |  |  |  |  |  |  |  |
| Quintile 1 | 2139 | 5.40 | Reference | | 2005 | 5.22 | Reference | | Reference | |
| Quintile 2 | 1991 | 5.25 | 0.92 (0.86, 0.98) | 0.014 | 1924 | 4.65 | 0.90 (0.84, 0.97) | 0.006 | 0.97 (0.88, 1.07) | 0.597 |
| Quintile 3 | 1789 | 5.22 | 0.88 (0.82, 0.95) | <0.001 | 2089 | 4.75 | 0.95 (0.89, 1.02) | 0.172 | 1.06 (0.96, 1.17) | 0.229 |
| Quintile 4 | 1670 | 5.55 | 0.89 (0.82, 0.96) | 0.002 | 2129 | 4.56 | 0.89 (0.83, 0.96) | 0.002 | 0.98 (0.89, 1.08) | 0.723 |
| Quintile 5 | 1311 | 5.37 | 0.82 (0.76, 0.89) | <0.001 | 2265 | 4.62 | 0.86 (0.80, 0.93) | <0.001 | 1.02 (0.92, 1.13) | 0.674 |
| Per 1-SD increase | NA | NA | 0.94 (0.91, 0.96) | <0.001 | NA | NA | 0.95 (0.93, 0.98) | <0.001 | 1.01 (0.98, 1.05) | 0.478 |
| **Total cholesterol** |  |  |  |  |  |  |  |  |  |  |
| Quintile 1 | 1514 | 4.96 | Reference | | 1982 | 4.80 | Reference | | Reference | |
| Quintile 2 | 1815 | 5.04 | 1.01 (0.93, 1.09) | 0.802 | 1908 | 4.54 | 1.00 (0.93, 1.08) | 0.944 | 0.98 (0.88, 1.09) | 0.702 |
| Quintile 3 | 1873 | 5.28 | 1.00 (0.92, 1.08) | 0.930 | 2009 | 4.58 | 0.99 (0.92, 1.07) | 0.809 | 0.98 (0.88, 1.08) | 0.659 |
| Quintile 4 | 1855 | 5.50 | 0.96 (0.89, 1.04) | 0.343 | 2178 | 4.83 | 1.03 (0.96, 1.12) | 0.408 | 1.06 (0.95, 1.17) | 0.293 |
| Quintile 5 | 1843 | 6.03 | 0.96 (0.88, 1.04) | 0.335 | 2335 | 4.96 | 0.94 (0.87, 1.01) | 0.111 | 0.96 (0.87, 1.07) | 0.491 |
| Per 1-SD increase | NA | NA | 0.97 (0.94, 0.99) | 0.017 | NA | NA | 0.98 (0.95, 1.00) | 0.046 | 1.00 (0.97, 1.04) | 0.776 |
| **HDL cholesterol** |  |  |  |  |  |  |  |  |  |  |
| Quintile 1 | 634 | 4.34 | Reference | | 1824 | 3.85 | Reference | | Reference | |
| Quintile 2 | 1045 | 4.56 | 1.01 (0.90, 1.12) | 0.916 | 2026 | 4.13 | 1.07 (1.00, 1.15) | 0.064 | 1.05 (0.92, 1.20) | 0.426 |
| Quintile 3 | 1534 | 4.92 | 1.07 (0.96, 1.19) | 0.238 | 2248 | 4.80 | 1.18 (1.09, 1.27) | <0.001 | 1.09 (0.97, 1.24) | 0.161 |
| Quintile 4 | 2253 | 5.36 | 1.12 (1.01, 1.24) | 0.038 | 2270 | 5.39 | 1.25 (1.16, 1.35) | <0.001 | 1.11 (0.98, 1.25) | 0.089 |
| Quintile 5 | 3434 | 6.18 | 1.17 (1.05, 1.30) | 0.003 | 2044 | 6.02 | 1.28 (1.18, 1.39) | <0.001 | 1.08 (0.96, 1.22) | 0.197 |
| Per 1-SD increase | NA | NA | 1.06 (1.04, 1.09) | <0.001 | NA | NA | 1.09 (1.06, 1.12) | <0.001 | 1.02 (0.99, 1.06) | 0.131 |
| **LDL cholesterol** |  |  |  |  |  |  |  |  |  |  |
| Quintile 1 | 1859 | 5.31 | Reference | | 2016 | 5.13 | Reference | | Reference | |
| Quintile 2 | 1966 | 5.21 | 0.97 (0.90, 1.04) | 0.382 | 1917 | 4.67 | 0.96 (0.89, 1.04) | 0.303 | 0.98 (0.89, 1.08) | 0.696 |
| Quintile 3 | 1862 | 5.30 | 0.94 (0.87, 1.02) | 0.125 | 2034 | 4.62 | 0.96 (0.89, 1.03) | 0.273 | 1.00 (0.90, 1.10) | 0.979 |
| Quintile 4 | 1728 | 5.50 | 0.93 (0.86, 1.00) | 0.053 | 2106 | 4.54 | 0.93 (0.86, 1.01) | 0.079 | 0.99 (0.89, 1.09) | 0.808 |
| Quintile 5 | 1485 | 5.50 | 0.88 (0.81, 0.96) | 0.003 | 2339 | 4.80 | 0.91 (0.84, 0.98) | 0.014 | 1.02 (0.92, 1.12) | 0.760 |
| Per 1-SD increase | NA | NA | 0.95 (0.92, 0.97) | <0.001 | NA | NA | 0.96 (0.94, 0.98) | 0.001 | 1.01 (0.98, 1.04) | 0.604 |
| **Triglycerides** |  |  |  |  |  |  |  |  |  |  |
| Quintile 1 | 2985 | 5.27 | Reference | | 1752 | 5.09 | Reference | | Reference | |
| Quintile 2 | 2285 | 5.48 | 0.92 (0.87, 0.98) | 0.008 | 2091 | 4.97 | 0.89 (0.83, 0.96) | 0.001 | 0.96 (0.88, 1.06) | 0.430 |
| Quintile 3 | 1706 | 5.44 | 0.89 (0.83, 0.95) | <0.001 | 2248 | 4.86 | 0.85 (0.80, 0.92) | <0.001 | 0.96 (0.88, 1.06) | 0.428 |
| Quintile 4 | 1178 | 5.27 | 0.84 (0.78, 0.91) | <0.001 | 2203 | 4.59 | 0.78 (0.73, 0.84) | <0.001 | 0.92 (0.83, 1.02) | 0.110 |
| Quintile 5 | 746 | 5.22 | 0.84 (0.76, 0.92) | <0.001 | 2118 | 4.35 | 0.76 (0.71, 0.82) | <0.001 | 0.90 (0.80, 1.01) | 0.065 |
| Per 1-SD increase | NA | NA | 0.92 (0.89, 0.95) | <0.001 | NA | NA | 0.93 (0.91, 0.95) | <0.001 | 1.00 (0.96, 1.05) | 0.859 |
| **Lipoprotein A** |  |  |  |  |  |  |  |  |  |  |
| Quintile 1 | 1747 | 5.37 | Reference | | 2015 | 4.68 | Reference | | Reference | |
| Quintile 2 | 1770 | 5.16 | 0.99 (0.92, 1.07) | 0.854 | 2051 | 4.74 | 1.01 (0.95, 1.08) | 0.701 | 1.02 (0.93, 1.13) | 0.673 |
| Quintile 3 | 1839 | 5.42 | 0.97 (0.91, 1.05) | 0.454 | 2075 | 4.66 | 0.97 (0.91, 1.04) | 0.417 | 1.00 (0.91, 1.11) | 0.924 |
| Quintile 4 | 1763 | 5.29 | 0.96 (0.89, 1.04) | 0.309 | 2150 | 4.90 | 1.04 (0.97, 1.12) | 0.228 | 1.09 (0.99, 1.21) | 0.081 |
| Quintile 5 | 1781 | 5.52 | 0.98 (0.91, 1.06) | 0.627 | 2121 | 4.75 | 0.96 (0.90, 1.03) | 0.218 | 0.98 (0.89, 1.09) | 0.761 |
| Per 1-SD increase | NA | NA | 0.99 (0.97, 1.02) | 0.611 | NA | NA | 0.99 (0.97, 1.01) | 0.318 | 1.00 (0.97, 1.03) | 0.926 |

| **Lipid traits** | **Normal weight (ref)** | | | | **Obese** | | | | **Ratio of HR  (95% CI)** | **P-value** |
| --- | --- | --- | --- | --- | --- | --- | --- | --- | --- | --- |
|  | **Event** | **Crude incidence rate** | **HR (95% CI)** | **P-value** | **Event** | **Crude incidence rate** | **HR (95% CI)** | **P-value** |  |  |
| **Apolipoprotein A** |  |  |  |  |  |  |  |  |  |  |
| Quintile 1 | 811 | 4.38 | Reference | | 1755 | 4.50 | Reference | | Reference | |
| Quintile 2 | 1111 | 4.45 | 0.97 (0.87, 1.07) | 0.547 | 1423 | 4.65 | 1.00 (0.92, 1.08) | 0.919 | 1.01 (0.89, 1.15) | 0.869 |
| Quintile 3 | 1635 | 5.06 | 1.09 (0.99, 1.20) | 0.092 | 1276 | 5.11 | 1.05 (0.96, 1.15) | 0.245 | 0.94 (0.83, 1.07) | 0.341 |
| Quintile 4 | 2178 | 5.44 | 1.08 (0.98, 1.18) | 0.131 | 1121 | 5.81 | 1.10 (1.00, 1.20) | 0.060 | 0.97 (0.86, 1.10) | 0.662 |
| Quintile 5 | 3165 | 6.27 | 1.10 (1.00, 1.21) | 0.056 | 812 | 6.40 | 1.18 (1.06, 1.31) | 0.002 | 1.01 (0.89, 1.15) | 0.882 |
| Per 1-SD increase | NA | NA | 1.04 (1.01, 1.06) | 0.003 | NA | NA | 1.08 (1.04, 1.12) | <0.001 | 1.01 (0.97, 1.05) | 0.567 |
| **Apolipoprotein B** |  |  |  |  |  |  |  |  |  |  |
| Quintile 1 | 2139 | 5.40 | Reference | | 1440 | 5.94 | Reference | | Reference | |
| Quintile 2 | 1991 | 5.25 | 0.92 (0.86, 0.98) | 0.014 | 1246 | 5.29 | 0.92 (0.84, 1.01) | 0.080 | 1.00 (0.90, 1.12) | 0.982 |
| Quintile 3 | 1789 | 5.22 | 0.88 (0.82, 0.95) | <0.001 | 1155 | 4.78 | 0.89 (0.81, 0.98) | 0.016 | 1.00 (0.90, 1.13) | 0.933 |
| Quintile 4 | 1670 | 5.55 | 0.89 (0.82, 0.96) | 0.002 | 1188 | 4.64 | 0.87 (0.78, 0.95) | 0.004 | 0.97 (0.87, 1.09) | 0.612 |
| Quintile 5 | 1311 | 5.37 | 0.82 (0.76, 0.89) | <0.001 | 1358 | 4.69 | 0.88 (0.79, 0.97) | 0.008 | 1.05 (0.94, 1.18) | 0.366 |
| Per 1-SD increase | NA | NA | 0.94 (0.91, 0.96) | <0.001 | NA | NA | 0.97 (0.94, 1.00) | 0.028 | 1.02 (0.99, 1.06) | 0.201 |
| **Total cholesterol** |  |  |  |  |  |  |  |  |  |  |
| Quintile 1 | 1514 | 4.96 | Reference | | 1787 | 5.70 | Reference | | Reference | |
| Quintile 2 | 1815 | 5.04 | 1.01 (0.93, 1.09) | 0.802 | 1223 | 4.92 | 0.92 (0.84, 1.01) | 0.084 | 0.91 (0.81, 1.02) | 0.114 |
| Quintile 3 | 1873 | 5.28 | 1.00 (0.92, 1.08) | 0.930 | 1051 | 4.51 | 0.90 (0.81, 0.99) | 0.028 | 0.90 (0.80, 1.01) | 0.067 |
| Quintile 4 | 1855 | 5.50 | 0.96 (0.89, 1.04) | 0.343 | 1134 | 4.85 | 0.94 (0.85, 1.04) | 0.261 | 0.97 (0.87, 1.09) | 0.649 |
| Quintile 5 | 1843 | 6.03 | 0.96 (0.88, 1.04) | 0.335 | 1192 | 5.03 | 0.94 (0.85, 1.04) | 0.251 | 0.97 (0.87, 1.09) | 0.624 |
| Per 1-SD increase | NA | NA | 0.97 (0.94, 0.99) | 0.017 | NA | NA | 0.99 (0.96, 1.02) | 0.512 | 1.01 (0.97, 1.05) | 0.616 |
| **HDL cholesterol** |  |  |  |  |  |  |  |  |  |  |
| Quintile 1 | 634 | 4.34 | Reference | | 1933 | 4.47 | Reference | | Reference | |
| Quintile 2 | 1045 | 4.56 | 1.01 (0.90, 1.12) | 0.916 | 1547 | 4.84 | 1.04 (0.96, 1.13) | 0.295 | 1.02 (0.89, 1.16) | 0.814 |
| Quintile 3 | 1534 | 4.92 | 1.07 (0.96, 1.19) | 0.238 | 1248 | 5.13 | 1.08 (0.98, 1.18) | 0.106 | 0.97 (0.85, 1.11) | 0.682 |
| Quintile 4 | 2253 | 5.36 | 1.12 (1.01, 1.24) | 0.038 | 986 | 5.66 | 1.14 (1.04, 1.26) | 0.008 | 0.97 (0.85, 1.11) | 0.653 |
| Quintile 5 | 3434 | 6.18 | 1.17 (1.05, 1.30) | 0.003 | 673 | 7.03 | 1.38 (1.24, 1.55) | <0.001 | 1.09 (0.95, 1.26) | 0.206 |
| Per 1-SD increase | NA | NA | 1.06 (1.04, 1.09) | <0.001 | NA | NA | 1.13 (1.09, 1.18) | <0.001 | 1.03 (0.99, 1.07) | 0.156 |
| **LDL cholesterol** |  |  |  |  |  |  |  |  |  |  |
| Quintile 1 | 1859 | 5.31 | Reference | | 1665 | 5.94 | Reference | | Reference | |
| Quintile 2 | 1966 | 5.21 | 0.97 (0.90, 1.04) | 0.382 | 1217 | 5.10 | 0.92 (0.84, 1.01) | 0.072 | 0.95 (0.85, 1.06) | 0.341 |
| Quintile 3 | 1862 | 5.30 | 0.94 (0.87, 1.02) | 0.125 | 1080 | 4.59 | 0.90 (0.81, 0.99) | 0.030 | 0.95 (0.85, 1.06) | 0.376 |
| Quintile 4 | 1728 | 5.50 | 0.93 (0.86, 1.00) | 0.053 | 1154 | 4.65 | 0.89 (0.81, 0.99) | 0.031 | 0.96 (0.86, 1.08) | 0.528 |
| Quintile 5 | 1485 | 5.50 | 0.88 (0.81, 0.96) | 0.003 | 1271 | 4.83 | 0.90 (0.82, 1.00) | 0.052 | 1.02 (0.91, 1.15) | 0.707 |
| Per 1-SD increase | NA | NA | 0.95 (0.92, 0.97) | <0.001 | NA | NA | 0.97 (0.94, 1.00) | 0.068 | 1.02 (0.98, 1.05) | 0.425 |
| **Triglycerides** |  |  |  |  |  |  |  |  |  |  |
| Quintile 1 | 2985 | 5.27 | Reference | | 578 | 5.32 | Reference | | Reference | |
| Quintile 2 | 2285 | 5.48 | 0.92 (0.87, 0.98) | 0.008 | 958 | 5.31 | 0.89 (0.78, 1.00) | 0.049 | 0.97 (0.85, 1.11) | 0.632 |
| Quintile 3 | 1706 | 5.44 | 0.89 (0.83, 0.95) | <0.001 | 1302 | 5.26 | 0.86 (0.77, 0.97) | 0.013 | 0.99 (0.87, 1.13) | 0.907 |
| Quintile 4 | 1178 | 5.27 | 0.84 (0.78, 0.91) | <0.001 | 1652 | 5.09 | 0.81 (0.73, 0.91) | <0.001 | 0.98 (0.86, 1.12) | 0.812 |
| Quintile 5 | 746 | 5.22 | 0.84 (0.76, 0.92) | <0.001 | 1897 | 4.69 | 0.76 (0.68, 0.85) | <0.001 | 0.93 (0.80, 1.07) | 0.284 |
| Per 1-SD increase | NA | NA | 0.92 (0.89, 0.95) | <0.001 | NA | NA | 0.93 (0.91, 0.96) | <0.001 | 1.02 (0.98, 1.06) | 0.382 |
| **Lipoprotein A** |  |  |  |  |  |  |  |  |  |  |
| Quintile 1 | 1747 | 5.37 | Reference | | 1333 | 4.88 | Reference | | Reference | |
| Quintile 2 | 1770 | 5.16 | 0.99 (0.92, 1.07) | 0.854 | 1274 | 5.11 | 1.04 (0.95, 1.14) | 0.398 | 1.05 (0.93, 1.17) | 0.435 |
| Quintile 3 | 1839 | 5.42 | 0.97 (0.91, 1.05) | 0.454 | 1187 | 4.99 | 1.02 (0.93, 1.11) | 0.721 | 1.04 (0.93, 1.17) | 0.500 |
| Quintile 4 | 1763 | 5.29 | 0.96 (0.89, 1.04) | 0.309 | 1273 | 5.05 | 1.04 (0.95, 1.13) | 0.439 | 1.07 (0.95, 1.20) | 0.248 |
| Quintile 5 | 1781 | 5.52 | 0.98 (0.91, 1.06) | 0.627 | 1320 | 5.22 | 1.06 (0.97, 1.16) | 0.205 | 1.07 (0.96, 1.20) | 0.215 |
| Per 1-SD increase | NA | NA | 0.99 (0.97, 1.02) | 0.611 | NA | NA | 1.02 (0.99, 1.04) | 0.286 | 1.02 (0.98, 1.06) | 0.270 |

Notes: BMI = Body-mass index; HR = Hazard ratio; CI = Confidence interval; HDL = High density lipoprotein; LDL = Low density lipoprotein; NA= Not available. The unit of crude incidence rate: events per 1,000 person-years. Model was adjusted for age, sex, ethnicity, deprivation index, current smokers, alcohol intake, sleep duration, total physical activity, total sedentary behavior, fruit and vegetables intake, red meat intake, processed meat intake, oily fish intake, ever eats eggs, ever eats dairy, waist-hip ratio, body fat percentage, number of morbidities, prefrail/frail status, cardiovascular disease, hypertension, diabetes , chronic kidney disease, cancer, anemia, positive rheumatoid factor, vitamin D deficiency, osteoporosis, falls in the last year, lipid-lowering medications, aspirin, glucocorticoids, vitamin D supplements, and calcium supplements.

# Supplementary Table 6. Associations between serum lipid concentrations and fracture risk, stratified by CVD.

| **Lipid traits** | **Non-CVD (ref)** | | | | **CVD** | | | | **Ratio of HR  (95% CI)** | **P-value** |
| --- | --- | --- | --- | --- | --- | --- | --- | --- | --- | --- |
|  | **Event** | **Crude incidence rate** | **HR (95% CI)** | **P-value** | **Event** | **Crude incidence rate** | **HR (95% CI)** | **P-value** |  |  |
| **Apolipoprotein A** |  |  |  |  |  |  |  |  |  |  |
| Quintile 1 | 3696 | 3.94 | Reference | | 729 | 6.14 | Reference | | Reference | |
| Quintile 2 | 4022 | 4.19 | 0.99 (0.94, 1.04) | 0.624 | 496 | 6.41 | 1.04 (0.90, 1.19) | 0.614 | 1.03 (0.90, 1.19) | 0.649 |
| Quintile 3 | 4663 | 4.79 | 1.07 (1.02, 1.13) | 0.007 | 459 | 7.79 | 1.23 (1.07, 1.42) | 0.004 | 1.13 (0.98, 1.30) | 0.095 |
| Quintile 4 | 5209 | 5.35 | 1.12 (1.06, 1.18) | <0.001 | 374 | 8.31 | 1.14 (0.98, 1.34) | 0.099 | 1.00 (0.86, 1.16) | 0.973 |
| Quintile 5 | 5922 | 6.11 | 1.14 (1.08, 1.20) | <0.001 | 348 | 10.20 | 1.24 (1.05, 1.47) | 0.012 | 1.07 (0.92, 1.25) | 0.379 |
| Per 1-SD increase | NA | NA | 1.05 (1.04, 1.07) | <0.001 | NA | NA | 1.10 (1.04, 1.16) | <0.001 | 1.03 (0.99, 1.08) | 0.179 |
| **Apolipoprotein B** |  |  |  |  |  |  |  |  |  |  |
| Quintile 1 | 4562 | 5.19 | Reference | | 1089 | 7.16 | Reference | | Reference | |
| Quintile 2 | 4640 | 4.85 | 0.90 (0.86, 0.95) | <0.001 | 587 | 7.53 | 0.99 (0.88, 1.12) | 0.891 | 1.13 (0.99, 1.28) | 0.060 |
| Quintile 3 | 4747 | 4.84 | 0.90 (0.86, 0.95) | <0.001 | 318 | 6.67 | 0.89 (0.77, 1.03) | 0.123 | 1.04 (0.90, 1.21) | 0.590 |
| Quintile 4 | 4795 | 4.82 | 0.87 (0.83, 0.91) | <0.001 | 223 | 7.12 | 0.90 (0.76, 1.07) | 0.229 | 1.15 (0.97, 1.36) | 0.118 |
| Quintile 5 | 4768 | 4.76 | 0.84 (0.80, 0.88) | <0.001 | 189 | 7.52 | 0.92 (0.76, 1.12) | 0.425 | 1.24 (1.03, 1.48) | 0.023 |
| Per 1-SD increase | NA | NA | 0.95 (0.93, 0.96) | <0.001 | NA | NA | 0.97 (0.92, 1.03) | 0.343 | 1.07 (1.01, 1.12) | 0.014 |
| **Total cholesterol** |  |  |  |  |  |  |  |  |  |  |
| Quintile 1 | 4036 | 4.76 | Reference | | 1286 | 6.81 | Reference | | Reference | |
| Quintile 2 | 4492 | 4.64 | 0.98 (0.93, 1.03) | 0.458 | 508 | 7.59 | 0.99 (0.88, 1.13) | 0.935 | 1.04 (0.92, 1.19) | 0.525 |
| Quintile 3 | 4716 | 4.73 | 0.97 (0.92, 1.02) | 0.176 | 256 | 7.30 | 0.92 (0.78, 1.08) | 0.302 | 1.03 (0.87, 1.21) | 0.765 |
| Quintile 4 | 5023 | 5.01 | 0.98 (0.93, 1.03) | 0.391 | 185 | 7.69 | 0.99 (0.82, 1.20) | 0.949 | 1.13 (0.95, 1.36) | 0.166 |
| Quintile 5 | 5245 | 5.26 | 0.94 (0.89, 0.99) | 0.012 | 171 | 8.92 | 1.07 (0.87, 1.31) | 0.507 | 1.28 (1.06, 1.54) | 0.010 |
| Per 1-SD increase | NA | NA | 0.97 (0.96, 0.99) | <0.001 | NA | NA | 1.02 (0.96, 1.08) | 0.586 | 1.08 (1.03, 1.13) | 0.003 |
| **HDL cholesterol** |  |  |  |  |  |  |  |  |  |  |
| Quintile 1 | 3590 | 3.91 | Reference | | 804 | 5.99 | Reference | | Reference | |
| Quintile 2 | 4114 | 4.27 | 1.04 (0.99, 1.10) | 0.110 | 519 | 6.70 | 1.07 (0.93, 1.22) | 0.348 | 1.01 (0.87, 1.16) | 0.936 |
| Quintile 3 | 4656 | 4.79 | 1.11 (1.05, 1.17) | <0.001 | 400 | 7.32 | 1.18 (1.02, 1.37) | 0.025 | 1.04 (0.90, 1.21) | 0.554 |
| Quintile 4 | 5188 | 5.29 | 1.17 (1.11, 1.23) | <0.001 | 368 | 9.17 | 1.30 (1.11, 1.52) | 0.001 | 1.08 (0.93, 1.26) | 0.318 |
| Quintile 5 | 5964 | 6.11 | 1.24 (1.18, 1.31) | <0.001 | 315 | 11.36 | 1.52 (1.28, 1.82) | <0.001 | 1.19 (1.02, 1.39) | 0.030 |
| Per 1-SD increase | NA | NA | 1.08 (1.06, 1.10) | <0.001 | NA | NA | 1.16 (1.09, 1.23) | <0.001 | 1.06 (1.01, 1.11) | 0.024 |
| **LDL cholesterol** |  |  |  |  |  |  |  |  |  |  |
| Quintile 1 | 4324 | 5.09 | Reference | | 1278 | 7.02 | Reference | | Reference | |
| Quintile 2 | 4632 | 4.81 | 0.95 (0.90, 0.99) | 0.022 | 524 | 7.48 | 0.97 (0.86, 1.10) | 0.678 | 1.07 (0.94, 1.21) | 0.324 |
| Quintile 3 | 4759 | 4.78 | 0.93 (0.89, 0.98) | 0.006 | 254 | 7.05 | 0.88 (0.75, 1.04) | 0.142 | 1.03 (0.88, 1.22) | 0.695 |
| Quintile 4 | 4839 | 4.82 | 0.91 (0.87, 0.96) | <0.001 | 183 | 7.33 | 0.90 (0.74, 1.09) | 0.296 | 1.12 (0.93, 1.34) | 0.225 |
| Quintile 5 | 4958 | 4.95 | 0.89 (0.84, 0.93) | <0.001 | 167 | 7.98 | 0.97 (0.79, 1.19) | 0.800 | 1.26 (1.04, 1.52) | 0.017 |
| Per 1-SD increase | NA | NA | 0.95 (0.94, 0.97) | <0.001 | NA | NA | 0.98 (0.92, 1.04) | 0.488 | 1.07 (1.02, 1.13) | 0.010 |
| **Triglycerides** |  |  |  |  |  |  |  |  |  |  |
| Quintile 1 | 5034 | 5.12 | Reference | | 391 | 7.93 | Reference | | Reference | |
| Quintile 2 | 4935 | 5.12 | 0.91 (0.87, 0.95) | <0.001 | 457 | 7.59 | 0.91 (0.78, 1.06) | 0.240 | 1.00 (0.85, 1.18) | 0.984 |
| Quintile 3 | 4793 | 5.00 | 0.87 (0.83, 0.91) | <0.001 | 489 | 7.22 | 0.87 (0.74, 1.02) | 0.086 | 0.99 (0.85, 1.16) | 0.906 |
| Quintile 4 | 4521 | 4.74 | 0.81 (0.77, 0.85) | <0.001 | 529 | 7.04 | 0.82 (0.70, 0.96) | 0.012 | 1.01 (0.86, 1.18) | 0.913 |
| Quintile 5 | 4229 | 4.44 | 0.78 (0.74, 0.82) | <0.001 | 540 | 6.60 | 0.78 (0.66, 0.91) | 0.002 | 1.00 (0.86, 1.17) | 0.987 |
| Per 1-SD increase | NA | NA | 0.93 (0.91, 0.94) | <0.001 | NA | NA | 0.93 (0.89, 0.98) | 0.006 | 1.01 (0.96, 1.06) | 0.710 |
| **Lipoprotein A** |  |  |  |  |  |  |  |  |  |  |
| Quintile 1 | 4692 | 4.83 | Reference | | 447 | 7.16 | Reference | | Reference | |
| Quintile 2 | 4702 | 4.84 | 1.01 (0.97, 1.06) | 0.533 | 444 | 7.47 | 1.05 (0.90, 1.22) | 0.559 | 1.04 (0.89, 1.21) | 0.658 |
| Quintile 3 | 4698 | 4.86 | 0.98 (0.94, 1.03) | 0.468 | 447 | 7.21 | 0.98 (0.84, 1.15) | 0.828 | 1.00 (0.85, 1.17) | 0.996 |
| Quintile 4 | 4753 | 4.93 | 1.01 (0.97, 1.06) | 0.652 | 479 | 7.41 | 1.04 (0.90, 1.21) | 0.585 | 1.04 (0.89, 1.21) | 0.635 |
| Quintile 5 | 4667 | 4.96 | 0.99 (0.95, 1.04) | 0.812 | 589 | 6.88 | 0.97 (0.84, 1.12) | 0.663 | 0.96 (0.83, 1.12) | 0.627 |
| Per 1-SD increase | NA | NA | 1.00 (0.98, 1.01) | 0.785 | NA | NA | 0.99 (0.95, 1.03) | 0.685 | 0.99 (0.94, 1.03) | 0.582 |

Notes: CVD = Cardiovascular disease; HR = Hazard ratio; CI = Confidence interval; HDL = High density lipoprotein; LDL = Low density lipoprotein; NA= Not available. The unit of crude incidence rate: events per 1,000 person-years. Model was adjusted for age, sex, ethnicity, deprivation index, current smokers, alcohol intake, sleep duration, total physical activity, total sedentary behavior, fruit and vegetables intake, red meat intake, processed meat intake, oily fish intake, ever eats eggs, ever eats dairy, body-mass index, waist-hip ratio, body fat percentage, number of morbidities, prefrail/frail status, hypertension, diabetes , chronic kidney disease, cancer, anemia, positive rheumatoid factor, vitamin D deficiency, osteoporosis, falls in the last year, lipid-lowering medications, aspirin, glucocorticoids, vitamin D supplements, and calcium supplements.

# Supplementary Table 7. Associations between serum lipid concentrations and fracture risk, stratified by use of lipid-lowering medications.

| **Lipid traits** | **Not taking LLM (ref)** | | | | **Taking LLM** | | | | **Ratio of HR  (95% CI)** | **P-value** |
| --- | --- | --- | --- | --- | --- | --- | --- | --- | --- | --- |
|  | **Event** | **Crude incidence rate** | **HR (95% CI)** | **P-value** | **Event** | **Crude incidence rate** | **HR (95% CI)** | **P-value** |  |  |
| **Apolipoprotein A** |  |  |  |  |  |  |  |  |  |  |
| Quintile 1 | 3134 | 3.85 | Reference | | 1291 | 5.32 | Reference | | Reference | |
| Quintile 2 | 3417 | 4.07 | 0.98 (0.93, 1.03) | 0.442 | 1101 | 5.56 | 1.04 (0.95, 1.15) | 0.394 | 1.05 (0.95, 1.17) | 0.345 |
| Quintile 3 | 4082 | 4.74 | 1.09 (1.03, 1.15) | 0.002 | 1040 | 6.09 | 1.08 (0.98, 1.19) | 0.124 | 0.98 (0.88, 1.09) | 0.654 |
| Quintile 4 | 4575 | 5.24 | 1.12 (1.06, 1.18) | <0.001 | 1008 | 6.85 | 1.13 (1.02, 1.25) | 0.023 | 0.98 (0.88, 1.10) | 0.773 |
| Quintile 5 | 5232 | 5.98 | 1.14 (1.07, 1.20) | <0.001 | 1038 | 8.11 | 1.21 (1.08, 1.35) | <0.001 | 1.03 (0.93, 1.15) | 0.533 |
| Per 1-SD increase | NA | NA | 1.05 (1.03, 1.07) | <0.001 | NA | NA | 1.08 (1.04, 1.12) | <0.001 | 1.01 (0.98, 1.05) | 0.391 |
| **Apolipoprotein B** |  |  |  |  |  |  |  |  |  |  |
| Quintile 1 | 3102 | 4.87 | Reference | | 2549 | 6.46 | Reference | | Reference | |
| Quintile 2 | 3873 | 4.80 | 0.91 (0.87, 0.96) | <0.001 | 1354 | 5.93 | 0.89 (0.83, 0.96) | 0.003 | 0.98 (0.89, 1.07) | 0.614 |
| Quintile 3 | 4280 | 4.78 | 0.89 (0.84, 0.93) | <0.001 | 785 | 5.83 | 0.94 (0.86, 1.03) | 0.182 | 1.06 (0.96, 1.18) | 0.254 |
| Quintile 4 | 4571 | 4.82 | 0.85 (0.81, 0.90) | <0.001 | 447 | 5.72 | 0.95 (0.85, 1.07) | 0.402 | 1.13 (1.00, 1.28) | 0.055 |
| Quintile 5 | 4614 | 4.73 | 0.82 (0.77, 0.86) | <0.001 | 343 | 6.74 | 1.08 (0.94, 1.23) | 0.272 | 1.34 (1.17, 1.54) | <0.001 |
| Per 1-SD increase | NA | NA | 0.94 (0.92, 0.95) | <0.001 | NA | NA | 1.00 (0.97, 1.04) | 0.847 | 1.08 (1.03, 1.12) | <0.001 |
| **Total cholesterol** |  |  |  |  |  |  |  |  |  |  |
| Quintile 1 | 2455 | 4.39 | Reference | | 2867 | 6.00 | Reference | | Reference | |
| Quintile 2 | 3708 | 4.49 | 0.97 (0.92, 1.03) | 0.336 | 1292 | 6.18 | 0.97 (0.89, 1.05) | 0.399 | 0.99 (0.90, 1.09) | 0.839 |
| Quintile 3 | 4305 | 4.66 | 0.94 (0.88, 0.99) | 0.022 | 667 | 6.25 | 1.01 (0.92, 1.12) | 0.823 | 1.08 (0.96, 1.20) | 0.194 |
| Quintile 4 | 4848 | 4.99 | 0.95 (0.89, 1.00) | 0.056 | 360 | 6.53 | 1.08 (0.95, 1.23) | 0.223 | 1.15 (1.00, 1.31) | 0.049 |
| Quintile 5 | 5124 | 5.23 | 0.90 (0.85, 0.95) | <0.001 | 292 | 7.74 | 1.23 (1.07, 1.42) | 0.004 | 1.38 (1.19, 1.60) | <0.001 |
| Per 1-SD increase | NA | NA | 0.96 (0.94, 0.98) | <0.001 | NA | NA | 1.05 (1.01, 1.09) | 0.021 | 1.09 (1.05, 1.13) | <0.001 |
| **HDL cholesterol** |  |  |  |  |  |  |  |  |  |  |
| Quintile 1 | 2825 | 3.75 | Reference | | 1569 | 5.24 | Reference | | Reference | |
| Quintile 2 | 3440 | 4.14 | 1.05 (0.99, 1.11) | 0.127 | 1193 | 5.65 | 1.05 (0.96, 1.15) | 0.299 | 0.99 (0.89, 1.10) | 0.846 |
| Quintile 3 | 4045 | 4.67 | 1.12 (1.05, 1.18) | <0.001 | 1011 | 6.30 | 1.13 (1.02, 1.24) | 0.016 | 0.99 (0.89, 1.10) | 0.783 |
| Quintile 4 | 4640 | 5.16 | 1.17 (1.11, 1.24) | <0.001 | 916 | 7.43 | 1.22 (1.10, 1.35) | <0.001 | 1.01 (0.91, 1.13) | 0.858 |
| Quintile 5 | 5490 | 6.02 | 1.24 (1.17, 1.32) | <0.001 | 789 | 8.56 | 1.38 (1.23, 1.55) | <0.001 | 1.08 (0.96, 1.20) | 0.199 |
| Per 1-SD increase | NA | NA | 1.08 (1.06, 1.10) | <0.001 | NA | NA | 1.12 (1.08, 1.17) | <0.001 | 1.03 (0.99, 1.06) | 0.137 |
| **LDL cholesterol** |  |  |  |  |  |  |  |  |  |  |
| Quintile 1 | 2594 | 4.71 | Reference | | 3008 | 6.26 | Reference | | Reference | |
| Quintile 2 | 3869 | 4.74 | 0.94 (0.89, 0.99) | 0.026 | 1287 | 5.96 | 0.93 (0.86, 1.00) | 0.050 | 0.99 (0.90, 1.08) | 0.756 |
| Quintile 3 | 4410 | 4.75 | 0.90 (0.86, 0.96) | <0.001 | 603 | 5.90 | 0.98 (0.88, 1.08) | 0.639 | 1.08 (0.97, 1.21) | 0.161 |
| Quintile 4 | 4711 | 4.82 | 0.88 (0.83, 0.93) | <0.001 | 311 | 6.03 | 1.03 (0.90, 1.17) | 0.667 | 1.18 (1.03, 1.36) | 0.020 |
| Quintile 5 | 4856 | 4.92 | 0.85 (0.80, 0.90) | <0.001 | 269 | 7.52 | 1.23 (1.06, 1.42) | 0.006 | 1.47 (1.26, 1.71) | <0.001 |
| Per 1-SD increase | NA | NA | 0.94 (0.92, 0.96) | <0.001 | NA | NA | 1.02 (0.99, 1.07) | 0.213 | 1.09 (1.05, 1.14) | <0.001 |
| **Triglycerides** |  |  |  |  |  |  |  |  |  |  |
| Quintile 1 | 4590 | 5.00 | Reference | | 835 | 7.26 | Reference | | Reference | |
| Quintile 2 | 4410 | 5.04 | 0.91 (0.87, 0.95) | <0.001 | 982 | 6.58 | 0.88 (0.80, 0.98) | 0.021 | 0.98 (0.87, 1.09) | 0.680 |
| Quintile 3 | 4236 | 5.00 | 0.89 (0.85, 0.94) | <0.001 | 1046 | 5.86 | 0.78 (0.70, 0.86) | <0.001 | 0.88 (0.79, 0.98) | 0.024 |
| Quintile 4 | 3798 | 4.62 | 0.81 (0.77, 0.86) | <0.001 | 1252 | 6.05 | 0.79 (0.71, 0.87) | <0.001 | 0.98 (0.87, 1.09) | 0.668 |
| Quintile 5 | 3406 | 4.27 | 0.78 (0.74, 0.82) | <0.001 | 1363 | 5.76 | 0.76 (0.68, 0.84) | <0.001 | 0.99 (0.89, 1.10) | 0.832 |
| Per 1-SD increase | NA | NA | 0.92 (0.90, 0.94) | <0.001 | NA | NA | 0.94 (0.91, 0.97) | <0.001 | 1.02 (0.99, 1.06) | 0.208 |
| **Lipoprotein A** |  |  |  |  |  |  |  |  |  |  |
| Quintile 1 | 4139 | 4.80 | Reference | | 1000 | 5.81 | Reference | | Reference | |
| Quintile 2 | 4147 | 4.74 | 1.00 (0.95, 1.04) | 0.849 | 999 | 6.43 | 1.11 (1.01, 1.23) | 0.037 | 1.12 (1.00, 1.25) | 0.048 |
| Quintile 3 | 4131 | 4.78 | 0.97 (0.92, 1.02) | 0.216 | 1014 | 6.18 | 1.04 (0.94, 1.14) | 0.496 | 1.07 (0.96, 1.19) | 0.233 |
| Quintile 4 | 4150 | 4.83 | 0.99 (0.95, 1.04) | 0.768 | 1082 | 6.34 | 1.10 (1.00, 1.22) | 0.048 | 1.12 (1.00, 1.24) | 0.047 |
| Quintile 5 | 3873 | 4.83 | 0.98 (0.93, 1.03) | 0.450 | 1383 | 6.17 | 1.04 (0.95, 1.14) | 0.435 | 1.06 (0.95, 1.17) | 0.299 |
| Per 1-SD increase | NA | NA | 1.00 (0.98, 1.01) | 0.786 | NA | NA | 1.00 (0.97, 1.02) | 0.775 | 1.00 (0.97, 1.03) | 0.894 |

Notes: LLM = Lipid-lowering medications; HR = Hazard ratio; CI = Confidence interval; HDL = High density lipoprotein; LDL = Low density lipoprotein; NA= Not available. The unit of crude incidence rate: events per 1,000 person-years. Model was adjusted for age, sex, ethnicity, deprivation index, current smokers, alcohol intake, sleep duration, total physical activity, total sedentary behavior, fruit and vegetables intake, red meat intake, processed meat intake, oily fish intake, ever eats eggs, ever eats dairy, body-mass index, waist-hip ratio, body fat percentage, number of morbidities, prefrail/frail status, cardiovascular disease, hypertension, diabetes , chronic kidney disease, cancer, anemia, positive rheumatoid factor, vitamin D deficiency, osteoporosis, falls in the last year, aspirin, glucocorticoids, vitamin D supplements, and calcium supplements.

# Supplementary Table 8. Associations between serum lipid traits and osteoporosis risk, stratified by age.

| **Lipid traits** | **Age ≥ 60 years (ref)** | | | | **Age < 60 years** | | | | **Ratio of HR  (95% CI)** | **P-value** |
| --- | --- | --- | --- | --- | --- | --- | --- | --- | --- | --- |
|  | **Event** | **Crude incidence rate** | **HR (95% CI)** | **P-value** | **Event** | **Crude incidence rate** | **HR (95% CI)** | **P-value** |  |  |
| **Apolipoprotein A** |  |  |  |  |  |  |  |  |  |  |
| Quintile 1 | 573 | 2.89 | Reference | | 332 | 1.03 | Reference | | Reference | |
| Quintile 2 | 693 | 3.36 | 0.94 (0.83, 1.08) | 0.393 | 389 | 1.29 | 1.11 (0.94, 1.32) | 0.223 | 1.10 (0.89, 1.36) | 0.375 |
| Quintile 3 | 930 | 4.39 | 1.05 (0.93, 1.19) | 0.437 | 481 | 1.64 | 1.16 (0.98, 1.37) | 0.080 | 1.00 (0.82, 1.22) | 0.991 |
| Quintile 4 | 1202 | 5.59 | 1.07 (0.95, 1.22) | 0.255 | 619 | 2.23 | 1.32 (1.12, 1.56) | <0.001 | 1.08 (0.89, 1.32) | 0.415 |
| Quintile 5 | 1576 | 7.26 | 1.13 (1.00, 1.28) | 0.051 | 796 | 3.09 | 1.56 (1.33, 1.85) | <0.001 | 1.20 (0.99, 1.44) | 0.058 |
| Per 1-SD increase | NA | NA | 1.05 (1.01, 1.08) | 0.012 | NA | NA | 1.16 (1.11, 1.21) | <0.001 | 1.07 (1.01, 1.12) | 0.012 |
| **Apolipoprotein B** |  |  |  |  |  |  |  |  |  |  |
| Quintile 1 | 995 | 4.66 | Reference | | 552 | 1.95 | Reference | | Reference | |
| Quintile 2 | 975 | 4.90 | 0.95 (0.86, 1.05) | 0.341 | 560 | 1.86 | 1.02 (0.89, 1.17) | 0.777 | 1.04 (0.88, 1.23) | 0.644 |
| Quintile 3 | 945 | 4.68 | 0.89 (0.79, 0.99) | 0.028 | 573 | 1.95 | 1.16 (1.01, 1.33) | 0.032 | 1.25 (1.06, 1.48) | 0.009 |
| Quintile 4 | 1022 | 4.88 | 0.90 (0.80, 1.00) | 0.050 | 470 | 1.63 | 1.08 (0.94, 1.25) | 0.294 | 1.14 (0.96, 1.36) | 0.128 |
| Quintile 5 | 1037 | 4.63 | 0.85 (0.76, 0.96) | 0.006 | 462 | 1.60 | 1.15 (0.99, 1.33) | 0.065 | 1.29 (1.09, 1.54) | 0.004 |
| Per 1-SD increase | NA | NA | 0.95 (0.92, 0.98) | 0.004 | NA | NA | 1.05 (1.00, 1.10) | 0.039 | 1.09 (1.03, 1.15) | 0.002 |
| **Total cholesterol** |  |  |  |  |  |  |  |  |  |  |
| Quintile 1 | 863 | 3.67 | Reference | | 419 | 1.56 | Reference | | Reference | |
| Quintile 2 | 862 | 4.60 | 1.03 (0.92, 1.16) | 0.578 | 503 | 1.62 | 1.06 (0.91, 1.23) | 0.440 | 0.95 (0.79, 1.14) | 0.565 |
| Quintile 3 | 963 | 5.07 | 1.04 (0.93, 1.18) | 0.487 | 554 | 1.80 | 1.25 (1.08, 1.45) | 0.003 | 1.07 (0.89, 1.28) | 0.488 |
| Quintile 4 | 1023 | 5.00 | 0.95 (0.85, 1.08) | 0.456 | 594 | 2.02 | 1.42 (1.22, 1.65) | <0.001 | 1.32 (1.10, 1.58) | 0.003 |
| Quintile 5 | 1263 | 5.46 | 0.96 (0.85, 1.08) | 0.498 | 547 | 2.01 | 1.37 (1.18, 1.60) | <0.001 | 1.27 (1.06, 1.53) | 0.009 |
| Per 1-SD increase | NA | NA | 0.97 (0.94, 1.01) | 0.153 | NA | NA | 1.14 (1.09, 1.20) | <0.001 | 1.13 (1.07, 1.20) | <0.001 |
| **HDL cholesterol** |  |  |  |  |  |  |  |  |  |  |
| Quintile 1 | 544 | 2.50 | Reference | | 286 | 0.94 | Reference | | Reference | |
| Quintile 2 | 725 | 3.42 | 1.05 (0.92, 1.20) | 0.482 | 373 | 1.25 | 1.08 (0.90, 1.30) | 0.393 | 0.96 (0.77, 1.19) | 0.701 |
| Quintile 3 | 911 | 4.34 | 1.14 (1.00, 1.29) | 0.050 | 444 | 1.52 | 1.13 (0.94, 1.35) | 0.194 | 0.86 (0.70, 1.07) | 0.172 |
| Quintile 4 | 1225 | 6.00 | 1.29 (1.14, 1.47) | <0.001 | 617 | 2.13 | 1.31 (1.10, 1.57) | 0.003 | 0.85 (0.69, 1.04) | 0.110 |
| Quintile 5 | 1569 | 7.67 | 1.31 (1.15, 1.49) | <0.001 | 897 | 3.29 | 1.79 (1.50, 2.14) | <0.001 | 1.10 (0.91, 1.34) | 0.315 |
| Per 1-SD increase | NA | NA | 1.08 (1.04, 1.12) | <0.001 | NA | NA | 1.23 (1.17, 1.29) | <0.001 | 1.07 (1.02, 1.12) | 0.008 |
| **LDL cholesterol** |  |  |  |  |  |  |  |  |  |  |
| Quintile 1 | 963 | 4.13 | Reference | | 502 | 1.88 | Reference | | Reference | |
| Quintile 2 | 948 | 4.99 | 1.05 (0.94, 1.17) | 0.387 | 543 | 1.78 | 0.98 (0.85, 1.13) | 0.800 | 0.87 (0.73, 1.04) | 0.117 |
| Quintile 3 | 973 | 5.03 | 1.02 (0.91, 1.15) | 0.676 | 552 | 1.82 | 1.14 (0.99, 1.31) | 0.067 | 1.01 (0.85, 1.20) | 0.902 |
| Quintile 4 | 968 | 4.73 | 0.91 (0.81, 1.02) | 0.118 | 523 | 1.76 | 1.20 (1.03, 1.38) | 0.016 | 1.18 (0.99, 1.41) | 0.061 |
| Quintile 5 | 1122 | 4.94 | 0.94 (0.83, 1.05) | 0.261 | 497 | 1.76 | 1.22 (1.05, 1.42) | 0.008 | 1.19 (1.00, 1.42) | 0.049 |
| Per 1-SD increase | NA | NA | 0.96 (0.92, 0.99) | 0.013 | NA | NA | 1.08 (1.03, 1.14) | <0.001 | 1.10 (1.04, 1.16) | <0.001 |
| **Triglycerides** |  |  |  |  |  |  |  |  |  |  |
| Quintile 1 | 959 | 6.36 | Reference | | 772 | 2.28 | Reference | | Reference | |
| Quintile 2 | 1147 | 5.65 | 0.93 (0.85, 1.03) | 0.158 | 585 | 2.00 | 1.00 (0.88, 1.12) | 0.950 | 1.10 (0.94, 1.28) | 0.219 |
| Quintile 3 | 1073 | 4.77 | 0.87 (0.79, 0.97) | 0.008 | 498 | 1.83 | 1.01 (0.89, 1.16) | 0.848 | 1.23 (1.05, 1.44) | 0.012 |
| Quintile 4 | 1049 | 4.35 | 0.87 (0.78, 0.97) | 0.009 | 419 | 1.57 | 0.96 (0.83, 1.11) | 0.625 | 1.22 (1.03, 1.44) | 0.020 |
| Quintile 5 | 746 | 3.27 | 0.77 (0.69, 0.86) | <0.001 | 343 | 1.20 | 0.81 (0.69, 0.96) | 0.014 | 1.21 (1.01, 1.46) | 0.039 |
| Per 1-SD increase | NA | NA | 0.90 (0.87, 0.94) | <0.001 | NA | NA | 0.95 (0.90, 1.01) | 0.104 | 1.11 (1.04, 1.18) | 0.001 |
| **Lipoprotein A** |  |  |  |  |  |  |  |  |  |  |
| Quintile 1 | 895 | 4.36 | Reference | | 537 | 1.80 | Reference | | Reference | |
| Quintile 2 | 880 | 4.38 | 0.98 (0.88, 1.09) | 0.701 | 522 | 1.72 | 0.94 (0.82, 1.08) | 0.391 | 0.97 (0.82, 1.15) | 0.718 |
| Quintile 3 | 1029 | 4.79 | 0.97 (0.88, 1.08) | 0.623 | 508 | 1.78 | 0.93 (0.81, 1.06) | 0.272 | 0.95 (0.80, 1.13) | 0.546 |
| Quintile 4 | 1052 | 5.05 | 1.05 (0.95, 1.16) | 0.390 | 544 | 1.91 | 1.02 (0.89, 1.17) | 0.730 | 0.98 (0.83, 1.16) | 0.826 |
| Quintile 5 | 1118 | 5.11 | 1.00 (0.91, 1.11) | 0.981 | 506 | 1.80 | 0.99 (0.87, 1.14) | 0.917 | 1.01 (0.86, 1.20) | 0.889 |
| Per 1-SD increase | NA | NA | 1.00 (0.97, 1.03) | 0.896 | NA | NA | 1.02 (0.97, 1.06) | 0.445 | 1.02 (0.97, 1.08) | 0.398 |

Notes: HR = Hazard ratio; CI = Confidence interval; HDL = High density lipoprotein; LDL = Low density lipoprotein; NA= Not available. The unit of crude incidence rate: events per 1,000 person-years. Model was adjusted for sex, ethnicity, deprivation index, current smokers, alcohol intake, sleep duration, total physical activity, total sedentary behavior, fruit and vegetables intake, red meat intake, processed meat intake, oily fish intake, ever eats eggs, ever eats dairy, body-mass index, waist-hip ratio, body fat percentage, number of morbidities, prefrail/frail status, cardiovascular disease, hypertension, diabetes , chronic kidney disease, cancer, anemia, positive rheumatoid factor, vitamin D deficiency, history of fractures, falls in the last year, lipid-lowering medications, aspirin, glucocorticoids, vitamin D supplements, and calcium supplements.

# Supplementary Table 9. Associations between serum lipid traits and osteoporosis risk, stratified by sex.

| **Lipid traits** | **Women (ref)** | | | | **Men** | | | | **Ratio of HR  (95% CI)** | **P-value** |
| --- | --- | --- | --- | --- | --- | --- | --- | --- | --- | --- |
|  | **Event** | **Crude incidence rate** | **HR (95% CI)** | **P-value** | **Event** | **Crude incidence rate** | **HR (95% CI)** | **P-value** |  |  |
| **Apolipoprotein A** |  |  |  |  |  |  |  |  |  |  |
| Quintile 1 | 524 | 3.85 | Reference | | 381 | 0.99 | Reference | | Reference | |
| Quintile 2 | 797 | 3.87 | 0.95 (0.83, 1.08) | 0.436 | 285 | 0.94 | 1.06 (0.89, 1.27) | 0.526 | 0.98 (0.79, 1.22) | 0.869 |
| Quintile 3 | 1190 | 4.39 | 1.01 (0.89, 1.14) | 0.869 | 221 | 0.94 | 1.06 (0.88, 1.29) | 0.527 | 0.89 (0.71, 1.11) | 0.286 |
| Quintile 4 | 1620 | 4.91 | 1.00 (0.89, 1.13) | 0.987 | 201 | 1.24 | 1.33 (1.09, 1.63) | 0.006 | 1.09 (0.87, 1.36) | 0.452 |
| Quintile 5 | 2192 | 5.80 | 1.07 (0.95, 1.20) | 0.296 | 180 | 1.85 | 1.49 (1.18, 1.88) | <0.001 | 1.18 (0.93, 1.50) | 0.165 |
| Per 1-SD increase | NA | NA | 1.03 (1.00, 1.06) | 0.075 | NA | NA | 1.16 (1.08, 1.25) | <0.001 | 1.07 (1.00, 1.15) | 0.067 |
| **Apolipoprotein B** |  |  |  |  |  |  |  |  |  |  |
| Quintile 1 | 1168 | 4.65 | Reference | | 379 | 1.54 | Reference | | Reference | |
| Quintile 2 | 1273 | 4.76 | 0.95 (0.86, 1.04) | 0.245 | 262 | 1.13 | 0.92 (0.77, 1.11) | 0.408 | 0.95 (0.78, 1.16) | 0.625 |
| Quintile 3 | 1271 | 4.79 | 0.93 (0.85, 1.02) | 0.137 | 247 | 1.07 | 0.89 (0.73, 1.09) | 0.269 | 0.94 (0.77, 1.16) | 0.558 |
| Quintile 4 | 1278 | 4.85 | 0.90 (0.81, 0.99) | 0.024 | 214 | 0.91 | 0.84 (0.69, 1.04) | 0.105 | 0.94 (0.76, 1.16) | 0.555 |
| Quintile 5 | 1333 | 4.88 | 0.90 (0.82, 0.99) | 0.038 | 166 | 0.69 | 0.68 (0.54, 0.86) | <0.001 | 0.77 (0.61, 0.96) | 0.022 |
| Per 1-SD increase | NA | NA | 0.97 (0.94, 1.00) | 0.030 | NA | NA | 0.88 (0.82, 0.95) | <0.001 | 0.92 (0.86, 0.99) | 0.018 |
| **Total cholesterol** |  |  |  |  |  |  |  |  |  |  |
| Quintile 1 | 856 | 4.24 | Reference | | 426 | 1.41 | Reference | | Reference | |
| Quintile 2 | 1100 | 4.39 | 1.04 (0.93, 1.15) | 0.499 | 265 | 1.07 | 0.91 (0.75, 1.10) | 0.323 | 0.86 (0.70, 1.06) | 0.157 |
| Quintile 3 | 1275 | 4.81 | 1.06 (0.95, 1.18) | 0.300 | 242 | 1.04 | 0.95 (0.78, 1.16) | 0.604 | 0.88 (0.72, 1.09) | 0.242 |
| Quintile 4 | 1424 | 5.03 | 1.02 (0.92, 1.14) | 0.677 | 193 | 0.89 | 0.83 (0.67, 1.03) | 0.093 | 0.80 (0.65, 1.00) | 0.047 |
| Quintile 5 | 1668 | 5.22 | 0.99 (0.89, 1.11) | 0.901 | 142 | 0.77 | 0.78 (0.62, 0.99) | 0.044 | 0.78 (0.62, 0.99) | 0.041 |
| Per 1-SD increase | NA | NA | 0.99 (0.96, 1.02) | 0.482 | NA | NA | 0.94 (0.87, 1.01) | 0.103 | 0.94 (0.88, 1.01) | 0.076 |
| **HDL cholesterol** |  |  |  |  |  |  |  |  |  |  |
| Quintile 1 | 463 | 3.86 | Reference | | 367 | 0.92 | Reference | | Reference | |
| Quintile 2 | 803 | 3.97 | 1.01 (0.88, 1.16) | 0.869 | 295 | 0.96 | 1.14 (0.95, 1.37) | 0.150 | 0.98 (0.78, 1.22) | 0.848 |
| Quintile 3 | 1132 | 4.16 | 1.05 (0.92, 1.20) | 0.462 | 223 | 0.97 | 1.12 (0.92, 1.36) | 0.274 | 0.88 (0.70, 1.10) | 0.262 |
| Quintile 4 | 1651 | 4.90 | 1.13 (0.99, 1.28) | 0.068 | 191 | 1.22 | 1.46 (1.19, 1.81) | <0.001 | 1.03 (0.82, 1.30) | 0.794 |
| Quintile 5 | 2274 | 5.84 | 1.19 (1.05, 1.36) | 0.007 | 192 | 2.18 | 2.01 (1.60, 2.52) | <0.001 | 1.34 (1.06, 1.69) | 0.013 |
| Per 1-SD increase | NA | NA | 1.07 (1.03, 1.10) | <0.001 | NA | NA | 1.21 (1.13, 1.31) | <0.001 | 1.08 (1.00, 1.16) | 0.046 |
| **LDL cholesterol** |  |  |  |  |  |  |  |  |  |  |
| Quintile 1 | 1062 | 4.57 | Reference | | 403 | 1.50 | Reference | | Reference | |
| Quintile 2 | 1219 | 4.65 | 1.01 (0.91, 1.11) | 0.865 | 272 | 1.17 | 0.92 (0.76, 1.11) | 0.364 | 0.90 (0.74, 1.10) | 0.326 |
| Quintile 3 | 1275 | 4.83 | 1.01 (0.91, 1.11) | 0.867 | 250 | 1.07 | 0.93 (0.76, 1.13) | 0.461 | 0.92 (0.75, 1.13) | 0.435 |
| Quintile 4 | 1309 | 4.90 | 0.95 (0.86, 1.05) | 0.342 | 182 | 0.78 | 0.74 (0.59, 0.93) | 0.009 | 0.78 (0.63, 0.97) | 0.028 |
| Quintile 5 | 1458 | 4.94 | 0.96 (0.87, 1.06) | 0.410 | 161 | 0.75 | 0.73 (0.57, 0.92) | 0.008 | 0.77 (0.61, 0.96) | 0.023 |
| Per 1-SD increase | NA | NA | 0.97 (0.94, 1.00) | 0.092 | NA | NA | 0.89 (0.82, 0.96) | 0.002 | 0.91 (0.85, 0.98) | 0.009 |
| **Triglycerides** |  |  |  |  |  |  |  |  |  |  |
| Quintile 1 | 1472 | 4.52 | Reference | | 259 | 1.58 | Reference | | Reference | |
| Quintile 2 | 1507 | 5.08 | 0.96 (0.88, 1.04) | 0.276 | 225 | 1.13 | 0.74 (0.60, 0.91) | 0.005 | 0.78 (0.63, 0.97) | 0.028 |
| Quintile 3 | 1337 | 4.98 | 0.92 (0.84, 1.00) | 0.064 | 234 | 1.03 | 0.67 (0.55, 0.83) | <0.001 | 0.76 (0.61, 0.95) | 0.015 |
| Quintile 4 | 1191 | 4.94 | 0.89 (0.81, 0.98) | 0.014 | 277 | 1.04 | 0.73 (0.60, 0.90) | 0.003 | 0.91 (0.73, 1.12) | 0.362 |
| Quintile 5 | 816 | 4.33 | 0.79 (0.71, 0.87) | <0.001 | 273 | 0.84 | 0.62 (0.50, 0.76) | <0.001 | 0.94 (0.75, 1.16) | 0.548 |
| Per 1-SD increase | NA | NA | 0.91 (0.88, 0.95) | <0.001 | NA | NA | 0.90 (0.84, 0.96) | 0.001 | 1.05 (0.97, 1.13) | 0.205 |
| **Lipoprotein A** |  |  |  |  |  |  |  |  |  |  |
| Quintile 1 | 1151 | 4.67 | Reference | | 281 | 1.09 | Reference | | Reference | |
| Quintile 2 | 1156 | 4.54 | 0.97 (0.89, 1.07) | 0.572 | 246 | 0.98 | 0.91 (0.74, 1.10) | 0.330 | 0.92 (0.74, 1.14) | 0.426 |
| Quintile 3 | 1276 | 4.73 | 0.92 (0.84, 1.00) | 0.056 | 261 | 1.13 | 1.05 (0.87, 1.28) | 0.594 | 1.09 (0.88, 1.34) | 0.439 |
| Quintile 4 | 1351 | 4.99 | 1.01 (0.92, 1.10) | 0.846 | 245 | 1.10 | 1.08 (0.88, 1.31) | 0.466 | 1.02 (0.82, 1.26) | 0.858 |
| Quintile 5 | 1389 | 4.98 | 0.97 (0.89, 1.06) | 0.463 | 235 | 1.06 | 1.01 (0.83, 1.23) | 0.922 | 0.99 (0.80, 1.23) | 0.933 |
| Per 1-SD increase | NA | NA | 1.00 (0.97, 1.03) | 0.982 | NA | NA | 1.01 (0.94, 1.07) | 0.861 | 0.99 (0.93, 1.07) | 0.876 |

Notes: HR = Hazard ratio; CI = Confidence interval; HDL = High density lipoprotein; LDL = Low density lipoprotein; NA= Not available. The unit of crude incidence rate: events per 1,000 person-years. Model was adjusted for age, ethnicity, deprivation index, current smokers, alcohol intake, sleep duration, total physical activity, total sedentary behavior, fruit and vegetables intake, red meat intake, processed meat intake, oily fish intake, ever eats eggs, ever eats dairy, body-mass index, waist-hip ratio, body fat percentage, number of morbidities, prefrail/frail status, cardiovascular disease, hypertension, diabetes , chronic kidney disease, cancer, anemia, positive rheumatoid factor, vitamin D deficiency, history of fractures, falls in the last year, menopause status (included in the model for women), lipid-lowering medications, aspirin, glucocorticoids, vitamin D supplements, and calcium supplements.

# Supplementary Table 10. Associations between serum lipid traits and osteoporosis risk, stratified by BMI.

| **Lipid traits** | **Normal weight (ref)** | | | | **Overweight** | | | | **Ratio of HR  (95% CI)** | **P-value** |
| --- | --- | --- | --- | --- | --- | --- | --- | --- | --- | --- |
|  | **Event** | **Crude incidence rate** | **HR (95% CI)** | **P-value** | **Event** | **Crude incidence rate** | **HR (95% CI)** | **P-value** |  |  |
| **Apolipoprotein A** |  |  |  |  |  |  |  |  |  |  |
| Quintile 1 | 228 | 2.63 | Reference | | 349 | 1.50 | Reference | | Reference | |
| Quintile 2 | 349 | 2.98 | 1.07 (0.88, 1.30) | 0.486 | 428 | 1.83 | 0.97 (0.82, 1.15) | 0.718 | 0.91 (0.71, 1.18) | 0.490 |
| Quintile 3 | 561 | 3.71 | 1.09 (0.91, 1.31) | 0.333 | 545 | 2.43 | 1.10 (0.93, 1.29) | 0.269 | 1.01 (0.80, 1.28) | 0.924 |
| Quintile 4 | 866 | 4.74 | 1.14 (0.95, 1.36) | 0.160 | 622 | 2.99 | 1.10 (0.93, 1.29) | 0.254 | 0.98 (0.78, 1.24) | 0.891 |
| Quintile 5 | 1357 | 5.86 | 1.16 (0.97, 1.38) | 0.105 | 731 | 4.17 | 1.19 (1.01, 1.41) | 0.036 | 1.04 (0.83, 1.29) | 0.749 |
| Per 1-SD increase | NA | NA | 1.03 (0.99, 1.07) | 0.174 | NA | NA | 1.06 (1.01, 1.11) | 0.021 | 1.03 (0.98, 1.09) | 0.258 |
| **Apolipoprotein B** |  |  |  |  |  |  |  |  |  |  |
| Quintile 1 | 681 | 3.77 | Reference | | 502 | 2.66 | Reference | | Reference | |
| Quintile 2 | 771 | 4.40 | 1.02 (0.91, 1.15) | 0.750 | 452 | 2.23 | 0.88 (0.75, 1.02) | 0.080 | 0.87 (0.72, 1.04) | 0.127 |
| Quintile 3 | 697 | 4.40 | 0.95 (0.84, 1.08) | 0.457 | 523 | 2.47 | 0.94 (0.81, 1.09) | 0.381 | 1.00 (0.83, 1.20) | 0.983 |
| Quintile 4 | 646 | 4.61 | 0.91 (0.80, 1.03) | 0.126 | 552 | 2.43 | 0.89 (0.77, 1.04) | 0.149 | 1.02 (0.85, 1.22) | 0.828 |
| Quintile 5 | 566 | 4.92 | 0.91 (0.80, 1.05) | 0.188 | 646 | 2.63 | 0.90 (0.77, 1.04) | 0.149 | 1.04 (0.87, 1.25) | 0.642 |
| Per 1-SD increase | NA | NA | 0.96 (0.92, 1.01) | 0.099 | NA | NA | 0.96 (0.92, 1.01) | 0.132 | 1.03 (0.97, 1.09) | 0.357 |
| **Total cholesterol** |  |  |  |  |  |  |  |  |  |  |
| Quintile 1 | 449 | 3.19 | Reference | | 437 | 2.16 | Reference | | Reference | |
| Quintile 2 | 627 | 3.78 | 1.03 (0.90, 1.19) | 0.638 | 426 | 2.09 | 0.97 (0.83, 1.15) | 0.748 | 0.94 (0.77, 1.15) | 0.554 |
| Quintile 3 | 722 | 4.37 | 1.04 (0.90, 1.19) | 0.615 | 521 | 2.45 | 1.11 (0.94, 1.31) | 0.202 | 1.06 (0.87, 1.29) | 0.542 |
| Quintile 4 | 738 | 4.74 | 0.97 (0.84, 1.12) | 0.643 | 597 | 2.71 | 1.11 (0.94, 1.31) | 0.232 | 1.14 (0.94, 1.39) | 0.172 |
| Quintile 5 | 825 | 5.80 | 0.98 (0.85, 1.13) | 0.797 | 694 | 2.94 | 1.01 (0.86, 1.20) | 0.872 | 1.07 (0.88, 1.29) | 0.499 |
| Per 1-SD increase | NA | NA | 0.99 (0.95, 1.04) | 0.761 | NA | NA | 0.99 (0.94, 1.04) | 0.748 | 1.02 (0.97, 1.08) | 0.443 |
| **HDL cholesterol** |  |  |  |  |  |  |  |  |  |  |
| Quintile 1 | 172 | 2.49 | Reference | | 305 | 1.33 | Reference | | Reference | |
| Quintile 2 | 302 | 2.84 | 0.96 (0.78, 1.20) | 0.736 | 457 | 1.92 | 1.19 (1.00, 1.41) | 0.050 | 1.22 (0.93, 1.61) | 0.150 |
| Quintile 3 | 508 | 3.52 | 1.08 (0.88, 1.32) | 0.463 | 553 | 2.39 | 1.28 (1.08, 1.51) | 0.005 | 1.18 (0.92, 1.52) | 0.199 |
| Quintile 4 | 855 | 4.40 | 1.09 (0.89, 1.33) | 0.394 | 669 | 3.23 | 1.44 (1.21, 1.70) | <0.001 | 1.29 (1.01, 1.65) | 0.040 |
| Quintile 5 | 1524 | 5.97 | 1.23 (1.01, 1.49) | 0.037 | 691 | 4.11 | 1.49 (1.25, 1.78) | <0.001 | 1.16 (0.91, 1.47) | 0.235 |
| Per 1-SD increase | NA | NA | 1.07 (1.02, 1.11) | 0.002 | NA | NA | 1.12 (1.06, 1.17) | <0.001 | 1.04 (0.98, 1.09) | 0.224 |
| **LDL cholesterol** |  |  |  |  |  |  |  |  |  |  |
| Quintile 1 | 603 | 3.71 | Reference | | 466 | 2.42 | Reference | | Reference | |
| Quintile 2 | 723 | 4.20 | 1.00 (0.89, 1.14) | 0.963 | 463 | 2.32 | 1.01 (0.86, 1.18) | 0.893 | 0.99 (0.82, 1.20) | 0.949 |
| Quintile 3 | 706 | 4.35 | 0.98 (0.86, 1.12) | 0.765 | 523 | 2.47 | 1.08 (0.92, 1.26) | 0.360 | 1.09 (0.90, 1.31) | 0.367 |
| Quintile 4 | 663 | 4.56 | 0.90 (0.79, 1.03) | 0.135 | 560 | 2.47 | 1.03 (0.87, 1.21) | 0.743 | 1.14 (0.95, 1.38) | 0.162 |
| Quintile 5 | 666 | 5.23 | 0.95 (0.83, 1.09) | 0.475 | 663 | 2.72 | 0.99 (0.84, 1.16) | 0.871 | 1.07 (0.89, 1.28) | 0.469 |
| Per 1-SD increase | NA | NA | 0.97 (0.93, 1.02) | 0.256 | NA | NA | 0.97 (0.93, 1.02) | 0.241 | 1.02 (0.96, 1.08) | 0.470 |
| **Triglycerides** |  |  |  |  |  |  |  |  |  |  |
| Quintile 1 | 1084 | 4.13 | Reference | | 461 | 2.74 | Reference | | Reference | |
| Quintile 2 | 913 | 4.71 | 0.98 (0.88, 1.08) | 0.623 | 559 | 2.69 | 0.83 (0.72, 0.96) | 0.012 | 0.90 (0.76, 1.07) | 0.223 |
| Quintile 3 | 668 | 4.66 | 0.93 (0.83, 1.04) | 0.209 | 598 | 2.67 | 0.79 (0.69, 0.91) | 0.001 | 0.93 (0.78, 1.11) | 0.406 |
| Quintile 4 | 455 | 4.39 | 0.89 (0.78, 1.01) | 0.070 | 614 | 2.59 | 0.80 (0.69, 0.92) | 0.002 | 1.01 (0.84, 1.21) | 0.950 |
| Quintile 5 | 241 | 3.64 | 0.78 (0.66, 0.92) | 0.003 | 443 | 1.86 | 0.64 (0.54, 0.74) | <0.001 | 0.94 (0.76, 1.16) | 0.554 |
| Per 1-SD increase | NA | NA | 0.92 (0.86, 0.97) | 0.004 | NA | NA | 0.87 (0.83, 0.92) | <0.001 | 1.00 (0.93, 1.08) | 0.959 |
| **Lipoprotein A** |  |  |  |  |  |  |  |  |  |  |
| Quintile 1 | 622 | 4.15 | Reference | | 491 | 2.33 | Reference | | Reference | |
| Quintile 2 | 665 | 4.15 | 1.01 (0.89, 1.14) | 0.912 | 447 | 2.09 | 0.90 (0.78, 1.04) | 0.142 | 0.90 (0.74, 1.08) | 0.264 |
| Quintile 3 | 690 | 4.37 | 0.89 (0.79, 1.01) | 0.072 | 551 | 2.52 | 0.99 (0.86, 1.14) | 0.907 | 1.13 (0.94, 1.35) | 0.197 |
| Quintile 4 | 699 | 4.62 | 1.00 (0.89, 1.12) | 0.976 | 587 | 2.77 | 1.07 (0.93, 1.22) | 0.350 | 1.10 (0.92, 1.32) | 0.306 |
| Quintile 5 | 685 | 4.56 | 0.93 (0.83, 1.05) | 0.265 | 599 | 2.72 | 0.96 (0.84, 1.10) | 0.581 | 1.05 (0.87, 1.25) | 0.619 |
| Per 1-SD increase | NA | NA | 0.99 (0.95, 1.02) | 0.465 | NA | NA | 1.00 (0.96, 1.04) | 0.824 | 1.01 (0.96, 1.07) | 0.618 |

| **Lipid traits** | **Normal weight (ref)** | | | | **Obese** | | | | **Ratio of HR  (95% CI)** | **P-value** |
| --- | --- | --- | --- | --- | --- | --- | --- | --- | --- | --- |
|  | **Event** | **Crude incidence rate** | **HR (95% CI)** | **P-value** | **Event** | **Crude incidence rate** | **HR (95% CI)** | **P-value** |  |  |
| **Apolipoprotein A** |  |  |  |  |  |  |  |  |  |  |
| Quintile 1 | 228 | 2.63 | Reference | | 320 | 1.59 | Reference | | Reference | |
| Quintile 2 | 349 | 2.98 | 1.07 (0.88, 1.30) | 0.486 | 293 | 1.87 | 0.92 (0.76, 1.12) | 0.400 | 0.81 (0.62, 1.06) | 0.128 |
| Quintile 3 | 561 | 3.71 | 1.09 (0.91, 1.31) | 0.333 | 289 | 2.26 | 0.90 (0.74, 1.10) | 0.317 | 0.75 (0.58, 0.97) | 0.027 |
| Quintile 4 | 866 | 4.74 | 1.14 (0.95, 1.36) | 0.160 | 311 | 3.13 | 0.99 (0.81, 1.21) | 0.924 | 0.78 (0.60, 1.00) | 0.053 |
| Quintile 5 | 1357 | 5.86 | 1.16 (0.97, 1.38) | 0.105 | 228 | 3.59 | 1.13 (0.91, 1.41) | 0.259 | 0.86 (0.66, 1.11) | 0.242 |
| Per 1-SD increase | NA | NA | 1.03 (0.99, 1.07) | 0.174 | NA | NA | 1.06 (0.99, 1.15) | 0.107 | 0.98 (0.91, 1.06) | 0.585 |
| **Apolipoprotein B** |  |  |  |  |  |  |  |  |  |  |
| Quintile 1 | 681 | 3.77 | Reference | | 331 | 2.68 | Reference | | Reference | |
| Quintile 2 | 771 | 4.40 | 1.02 (0.91, 1.15) | 0.750 | 283 | 2.38 | 0.84 (0.69, 1.03) | 0.087 | 0.85 (0.68, 1.07) | 0.170 |
| Quintile 3 | 697 | 4.40 | 0.95 (0.84, 1.08) | 0.457 | 271 | 2.19 | 0.83 (0.68, 1.02) | 0.078 | 0.93 (0.74, 1.17) | 0.528 |
| Quintile 4 | 646 | 4.61 | 0.91 (0.80, 1.03) | 0.126 | 278 | 2.13 | 0.86 (0.70, 1.06) | 0.167 | 1.04 (0.83, 1.30) | 0.712 |
| Quintile 5 | 566 | 4.92 | 0.91 (0.80, 1.05) | 0.188 | 278 | 1.84 | 0.72 (0.58, 0.89) | 0.002 | 0.87 (0.70, 1.10) | 0.246 |
| Per 1-SD increase | NA | NA | 0.96 (0.92, 1.01) | 0.099 | NA | NA | 0.91 (0.85, 0.97) | 0.006 | 0.98 (0.91, 1.05) | 0.624 |
| **Total cholesterol** |  |  |  |  |  |  |  |  |  |  |
| Quintile 1 | 449 | 3.19 | Reference | | 374 | 2.35 | Reference | | Reference | |
| Quintile 2 | 627 | 3.78 | 1.03 (0.90, 1.19) | 0.638 | 290 | 2.29 | 1.00 (0.82, 1.21) | 0.961 | 0.99 (0.79, 1.25) | 0.960 |
| Quintile 3 | 722 | 4.37 | 1.04 (0.90, 1.19) | 0.615 | 249 | 2.12 | 0.91 (0.73, 1.13) | 0.385 | 0.93 (0.74, 1.18) | 0.559 |
| Quintile 4 | 738 | 4.74 | 0.97 (0.84, 1.12) | 0.643 | 259 | 2.14 | 0.87 (0.70, 1.08) | 0.212 | 0.97 (0.77, 1.23) | 0.823 |
| Quintile 5 | 825 | 5.80 | 0.98 (0.85, 1.13) | 0.797 | 269 | 2.18 | 0.81 (0.65, 1.01) | 0.068 | 0.90 (0.72, 1.13) | 0.367 |
| Per 1-SD increase | NA | NA | 0.99 (0.95, 1.04) | 0.761 | NA | NA | 0.93 (0.87, 1.00) | 0.059 | 0.97 (0.90, 1.04) | 0.367 |
| **HDL cholesterol** |  |  |  |  |  |  |  |  |  |  |
| Quintile 1 | 172 | 2.49 | Reference | | 348 | 1.57 | Reference | | Reference | |
| Quintile 2 | 302 | 2.84 | 0.96 (0.78, 1.20) | 0.736 | 330 | 2.01 | 1.01 (0.84, 1.21) | 0.923 | 0.97 (0.73, 1.28) | 0.809 |
| Quintile 3 | 508 | 3.52 | 1.08 (0.88, 1.32) | 0.463 | 280 | 2.25 | 0.87 (0.71, 1.07) | 0.189 | 0.74 (0.56, 0.97) | 0.030 |
| Quintile 4 | 855 | 4.40 | 1.09 (0.89, 1.33) | 0.394 | 294 | 3.29 | 1.24 (1.01, 1.52) | 0.037 | 1.01 (0.78, 1.31) | 0.959 |
| Quintile 5 | 1524 | 5.97 | 1.23 (1.01, 1.49) | 0.037 | 189 | 3.90 | 1.23 (0.97, 1.56) | 0.086 | 0.87 (0.66, 1.15) | 0.327 |
| Per 1-SD increase | NA | NA | 1.07 (1.02, 1.11) | 0.002 | NA | NA | 1.10 (1.02, 1.19) | 0.020 | 0.98 (0.90, 1.06) | 0.548 |
| **LDL cholesterol** |  |  |  |  |  |  |  |  |  |  |
| Quintile 1 | 603 | 3.71 | Reference | | 364 | 2.56 | Reference | | Reference | |
| Quintile 2 | 723 | 4.20 | 1.00 (0.89, 1.14) | 0.963 | 280 | 2.31 | 0.95 (0.78, 1.16) | 0.623 | 0.99 (0.79, 1.24) | 0.958 |
| Quintile 3 | 706 | 4.35 | 0.98 (0.86, 1.12) | 0.765 | 271 | 2.26 | 0.95 (0.77, 1.17) | 0.637 | 1.05 (0.84, 1.31) | 0.698 |
| Quintile 4 | 663 | 4.56 | 0.90 (0.79, 1.03) | 0.135 | 246 | 1.94 | 0.81 (0.64, 1.01) | 0.057 | 0.99 (0.78, 1.24) | 0.901 |
| Quintile 5 | 666 | 5.23 | 0.95 (0.83, 1.09) | 0.475 | 280 | 2.03 | 0.80 (0.64, 1.00) | 0.046 | 0.93 (0.75, 1.17) | 0.552 |
| Per 1-SD increase | NA | NA | 0.97 (0.93, 1.02) | 0.256 | NA | NA | 0.91 (0.85, 0.98) | 0.009 | 0.97 (0.91, 1.04) | 0.442 |
| **Triglycerides** |  |  |  |  |  |  |  |  |  |  |
| Quintile 1 | 1084 | 4.13 | Reference | | 135 | 2.52 | Reference | | Reference | |
| Quintile 2 | 913 | 4.71 | 0.98 (0.88, 1.08) | 0.623 | 229 | 2.51 | 0.80 (0.62, 1.03) | 0.079 | 0.83 (0.63, 1.08) | 0.170 |
| Quintile 3 | 668 | 4.66 | 0.93 (0.83, 1.04) | 0.209 | 287 | 2.25 | 0.76 (0.60, 0.97) | 0.028 | 0.84 (0.65, 1.10) | 0.206 |
| Quintile 4 | 455 | 4.39 | 0.89 (0.78, 1.01) | 0.070 | 391 | 2.35 | 0.76 (0.60, 0.96) | 0.022 | 0.90 (0.69, 1.16) | 0.413 |
| Quintile 5 | 241 | 3.64 | 0.78 (0.66, 0.92) | 0.003 | 399 | 1.91 | 0.73 (0.58, 0.93) | 0.009 | 1.02 (0.77, 1.35) | 0.893 |
| Per 1-SD increase | NA | NA | 0.92 (0.86, 0.97) | 0.004 | NA | NA | 0.93 (0.87, 1.00) | 0.035 | 1.07 (0.98, 1.16) | 0.145 |
| **Lipoprotein A** |  |  |  |  |  |  |  |  |  |  |
| Quintile 1 | 622 | 4.15 | Reference | | 296 | 2.09 | Reference | | Reference | |
| Quintile 2 | 665 | 4.15 | 1.01 (0.89, 1.14) | 0.912 | 264 | 2.05 | 0.95 (0.78, 1.16) | 0.607 | 0.94 (0.75, 1.18) | 0.593 |
| Quintile 3 | 690 | 4.37 | 0.89 (0.79, 1.01) | 0.072 | 274 | 2.25 | 0.96 (0.79, 1.17) | 0.677 | 1.04 (0.83, 1.31) | 0.719 |
| Quintile 4 | 699 | 4.62 | 1.00 (0.89, 1.12) | 0.976 | 293 | 2.30 | 1.01 (0.84, 1.23) | 0.891 | 1.00 (0.79, 1.25) | 0.975 |
| Quintile 5 | 685 | 4.56 | 0.93 (0.83, 1.05) | 0.265 | 314 | 2.44 | 1.11 (0.92, 1.34) | 0.262 | 1.15 (0.93, 1.44) | 0.203 |
| Per 1-SD increase | NA | NA | 0.99 (0.95, 1.02) | 0.465 | NA | NA | 1.05 (0.99, 1.11) | 0.107 | 1.05 (0.98, 1.13) | 0.141 |

Notes: BMI = Body-mass index; HR = Hazard ratio; CI = Confidence interval; HDL = High density lipoprotein; LDL = Low density lipoprotein; NA= Not available. The unit of crude incidence rate: events per 1,000 person-years. Model was adjusted for age, sex, ethnicity, deprivation index, current smokers, alcohol intake, sleep duration, total physical activity, total sedentary behavior, fruit and vegetables intake, red meat intake, processed meat intake, oily fish intake, ever eats eggs, ever eats dairy, waist-hip ratio, body fat percentage, number of morbidities, prefrail/frail status, cardiovascular disease, hypertension, diabetes , chronic kidney disease, cancer, anemia, positive rheumatoid factor, vitamin D deficiency, history of fractures, falls in the last year, lipid-lowering medications, aspirin, glucocorticoids, vitamin D supplements, and calcium supplements.

# Supplementary Table 11. Associations between serum lipid traits and osteoporosis risk, stratified by CVD.

| **Lipid traits** | **Non-CVD (ref)** | | | | **CVD** | | | | **Ratio of HR  (95% CI)** | **P-value** |
| --- | --- | --- | --- | --- | --- | --- | --- | --- | --- | --- |
|  | **Event** | **Crude incidence rate** | **HR (95% CI)** | **P-value** | **Event** | **Crude incidence rate** | **HR (95% CI)** | **P-value** |  |  |
| **Apolipoprotein A** |  |  |  |  |  |  |  |  |  |  |
| Quintile 1 | 751 | 1.63 | Reference | | 154 | 2.55 | Reference | | Reference | |
| Quintile 2 | 943 | 2.01 | 0.94 (0.84, 1.06) | 0.316 | 139 | 3.50 | 1.12 (0.85, 1.47) | 0.436 | 1.08 (0.81, 1.45) | 0.595 |
| Quintile 3 | 1275 | 2.68 | 0.99 (0.89, 1.10) | 0.812 | 136 | 4.53 | 1.33 (1.00, 1.76) | 0.051 | 1.16 (0.87, 1.54) | 0.322 |
| Quintile 4 | 1677 | 3.57 | 1.01 (0.91, 1.12) | 0.829 | 144 | 6.38 | 1.57 (1.17, 2.11) | 0.003 | 1.23 (0.93, 1.63) | 0.149 |
| Quintile 5 | 2223 | 4.85 | 1.08 (0.97, 1.20) | 0.179 | 149 | 8.85 | 1.65 (1.21, 2.25) | 0.001 | 1.16 (0.88, 1.54) | 0.284 |
| Per 1-SD increase | NA | NA | 1.03 (1.00, 1.06) | 0.035 | NA | NA | 1.18 (1.08, 1.30) | <0.001 | 1.05 (0.97, 1.15) | 0.207 |
| **Apolipoprotein B** |  |  |  |  |  |  |  |  |  |  |
| Quintile 1 | 1218 | 2.90 | Reference | | 329 | 4.34 | Reference | | Reference | |
| Quintile 2 | 1376 | 3.00 | 0.96 (0.88, 1.05) | 0.426 | 159 | 3.92 | 0.77 (0.62, 0.96) | 0.023 | 0.85 (0.67, 1.07) | 0.164 |
| Quintile 3 | 1416 | 3.00 | 0.93 (0.85, 1.02) | 0.147 | 102 | 4.16 | 0.84 (0.64, 1.09) | 0.189 | 0.97 (0.74, 1.27) | 0.841 |
| Quintile 4 | 1415 | 2.93 | 0.89 (0.81, 0.98) | 0.016 | 77 | 4.93 | 0.85 (0.63, 1.16) | 0.311 | 1.04 (0.77, 1.40) | 0.808 |
| Quintile 5 | 1444 | 2.89 | 0.88 (0.80, 0.97) | 0.007 | 55 | 4.26 | 0.71 (0.50, 1.02) | 0.061 | 0.90 (0.64, 1.26) | 0.530 |
| Per 1-SD increase | NA | NA | 0.96 (0.93, 0.98) | 0.003 | NA | NA | 0.90 (0.81, 0.99) | 0.035 | 0.98 (0.89, 1.07) | 0.598 |
| **Total cholesterol** |  |  |  |  |  |  |  |  |  |  |
| Quintile 1 | 937 | 2.29 | Reference | | 345 | 3.63 | Reference | | Reference | |
| Quintile 2 | 1212 | 2.61 | 1.01 (0.91, 1.12) | 0.825 | 153 | 4.51 | 0.96 (0.76, 1.21) | 0.716 | 0.96 (0.75, 1.22) | 0.720 |
| Quintile 3 | 1416 | 2.95 | 1.04 (0.94, 1.15) | 0.465 | 101 | 5.60 | 0.93 (0.70, 1.24) | 0.632 | 0.96 (0.73, 1.27) | 0.767 |
| Quintile 4 | 1548 | 3.18 | 0.99 (0.89, 1.09) | 0.803 | 69 | 5.43 | 0.96 (0.70, 1.32) | 0.809 | 1.00 (0.74, 1.35) | 0.979 |
| Quintile 5 | 1756 | 3.56 | 0.95 (0.86, 1.06) | 0.357 | 54 | 5.53 | 0.95 (0.67, 1.35) | 0.776 | 1.03 (0.75, 1.43) | 0.841 |
| Per 1-SD increase | NA | NA | 0.98 (0.95, 1.01) | 0.222 | NA | NA | 0.96 (0.87, 1.07) | 0.483 | 1.00 (0.91, 1.09) | 0.971 |
| **HDL cholesterol** |  |  |  |  |  |  |  |  |  |  |
| Quintile 1 | 670 | 1.48 | Reference | | 160 | 2.32 | Reference | | Reference | |
| Quintile 2 | 944 | 2.01 | 0.99 (0.88, 1.11) | 0.852 | 154 | 3.90 | 1.32 (1.01, 1.73) | 0.045 | 1.20 (0.91, 1.60) | 0.200 |
| Quintile 3 | 1222 | 2.58 | 1.03 (0.92, 1.15) | 0.616 | 133 | 4.80 | 1.45 (1.09, 1.94) | 0.011 | 1.16 (0.87, 1.55) | 0.319 |
| Quintile 4 | 1713 | 3.61 | 1.13 (1.01, 1.27) | 0.031 | 129 | 6.66 | 1.93 (1.43, 2.60) | <0.001 | 1.37 (1.03, 1.81) | 0.031 |
| Quintile 5 | 2320 | 5.01 | 1.22 (1.09, 1.37) | <0.001 | 146 | 10.48 | 2.13 (1.55, 2.92) | <0.001 | 1.27 (0.97, 1.68) | 0.087 |
| Per 1-SD increase | NA | NA | 1.07 (1.04, 1.11) | <0.001 | NA | NA | 1.23 (1.12, 1.36) | <0.001 | 1.06 (0.97, 1.15) | 0.184 |
| **LDL cholesterol** |  |  |  |  |  |  |  |  |  |  |
| Quintile 1 | 1088 | 2.66 | Reference | | 377 | 4.11 | Reference | | Reference | |
| Quintile 2 | 1350 | 2.94 | 1.04 (0.95, 1.14) | 0.421 | 141 | 3.95 | 0.72 (0.57, 0.91) | 0.006 | 0.74 (0.58, 0.95) | 0.017 |
| Quintile 3 | 1432 | 3.00 | 1.03 (0.93, 1.13) | 0.602 | 93 | 5.00 | 0.83 (0.63, 1.11) | 0.206 | 0.89 (0.68, 1.18) | 0.428 |
| Quintile 4 | 1434 | 2.93 | 0.94 (0.85, 1.04) | 0.235 | 57 | 4.44 | 0.75 (0.53, 1.07) | 0.115 | 0.89 (0.64, 1.24) | 0.481 |
| Quintile 5 | 1565 | 3.14 | 0.95 (0.86, 1.04) | 0.270 | 54 | 5.11 | 0.81 (0.57, 1.15) | 0.240 | 0.98 (0.70, 1.35) | 0.883 |
| Per 1-SD increase | NA | NA | 0.96 (0.93, 0.99) | 0.016 | NA | NA | 0.90 (0.81, 1.00) | 0.048 | 0.98 (0.89, 1.07) | 0.594 |
| **Triglycerides** |  |  |  |  |  |  |  |  |  |  |
| Quintile 1 | 1588 | 3.41 | Reference | | 143 | 5.99 | Reference | | Reference | |
| Quintile 2 | 1577 | 3.39 | 0.92 (0.85, 1.00) | 0.043 | 155 | 5.20 | 0.83 (0.64, 1.07) | 0.154 | 0.93 (0.71, 1.22) | 0.612 |
| Quintile 3 | 1437 | 3.11 | 0.89 (0.82, 0.97) | 0.008 | 134 | 3.91 | 0.62 (0.46, 0.82) | <0.001 | 0.73 (0.55, 0.97) | 0.032 |
| Quintile 4 | 1299 | 2.77 | 0.87 (0.79, 0.95) | 0.001 | 169 | 4.37 | 0.70 (0.53, 0.92) | 0.011 | 0.88 (0.67, 1.14) | 0.330 |
| Quintile 5 | 968 | 2.06 | 0.77 (0.70, 0.85) | <0.001 | 121 | 2.82 | 0.55 (0.41, 0.75) | <0.001 | 0.80 (0.60, 1.07) | 0.130 |
| Per 1-SD increase | NA | NA | 0.92 (0.88, 0.95) | <0.001 | NA | NA | 0.83 (0.75, 0.93) | <0.001 | 0.94 (0.85, 1.04) | 0.252 |
| **Lipoprotein A** |  |  |  |  |  |  |  |  |  |  |
| Quintile 1 | 1291 | 2.73 | Reference | | 141 | 4.49 | Reference | | Reference | |
| Quintile 2 | 1293 | 2.73 | 0.98 (0.90, 1.06) | 0.590 | 109 | 3.55 | 0.80 (0.59, 1.08) | 0.147 | 0.83 (0.61, 1.13) | 0.242 |
| Quintile 3 | 1389 | 2.96 | 0.93 (0.85, 1.01) | 0.084 | 148 | 4.65 | 0.98 (0.74, 1.29) | 0.897 | 1.07 (0.80, 1.42) | 0.649 |
| Quintile 4 | 1455 | 3.16 | 1.01 (0.93, 1.10) | 0.779 | 141 | 4.39 | 1.08 (0.82, 1.43) | 0.572 | 1.06 (0.80, 1.41) | 0.680 |
| Quintile 5 | 1441 | 3.15 | 0.97 (0.89, 1.05) | 0.435 | 183 | 4.21 | 1.01 (0.78, 1.32) | 0.928 | 1.03 (0.78, 1.35) | 0.839 |
| Per 1-SD increase | NA | NA | 1.00 (0.97, 1.02) | 0.846 | NA | NA | 1.03 (0.95, 1.11) | 0.506 | 1.02 (0.94, 1.10) | 0.680 |

Notes: CVD = Cardiovascular disease; HR = Hazard ratio; CI = Confidence interval; HDL = High density lipoprotein; LDL = Low density lipoprotein; NA= Not available. The unit of crude incidence rate: events per 1,000 person-years. Model was adjusted for age, sex, ethnicity, deprivation index, current smokers, alcohol intake, sleep duration, total physical activity, total sedentary behavior, fruit and vegetables intake, red meat intake, processed meat intake, oily fish intake, ever eats eggs, ever eats dairy, body-mass index, waist-hip ratio, body fat percentage, number of morbidities, prefrail/frail status, hypertension, diabetes , chronic kidney disease, cancer, anemia, positive rheumatoid factor, vitamin D deficiency, history of fractures, falls in the last year, lipid-lowering medications, aspirin, glucocorticoids, vitamin D supplements, and calcium supplements.

# Supplementary Table 12. Associations between serum lipid concentrations and osteoporosis risk, stratified by use of lipid-lowering medications.

| **Lipid traits** | **Not taking LLM (ref)** | | | | **Taking LLM** | | | | **Ratio of HR  (95% CI)** | **P-value** |
| --- | --- | --- | --- | --- | --- | --- | --- | --- | --- | --- |
|  | **Event** | **Crude incidence rate** | **HR (95% CI)** | **P-value** | **Event** | **Crude incidence rate** | **HR (95% CI)** | **P-value** |  |  |
| **Apolipoprotein A** |  |  |  |  |  |  |  |  |  |  |
| Quintile 1 | 657 | 1.64 | Reference | | 248 | 2.03 | Reference | | Reference | |
| Quintile 2 | 831 | 2.03 | 0.95 (0.84, 1.07) | 0.390 | 251 | 2.54 | 1.02 (0.82, 1.26) | 0.871 | 1.05 (0.83, 1.33) | 0.700 |
| Quintile 3 | 1125 | 2.68 | 0.98 (0.87, 1.10) | 0.735 | 286 | 3.34 | 1.18 (0.96, 1.45) | 0.124 | 1.16 (0.93, 1.46) | 0.195 |
| Quintile 4 | 1530 | 3.65 | 1.03 (0.92, 1.15) | 0.652 | 291 | 4.01 | 1.15 (0.93, 1.43) | 0.196 | 1.07 (0.85, 1.34) | 0.584 |
| Quintile 5 | 1948 | 4.71 | 1.03 (0.92, 1.16) | 0.554 | 424 | 6.90 | 1.60 (1.29, 1.98) | <0.001 | 1.45 (1.17, 1.79) | <0.001 |
| Per 1-SD increase | NA | NA | 1.01 (0.98, 1.05) | 0.370 | NA | NA | 1.17 (1.10, 1.25) | <0.001 | 1.13 (1.06, 1.20) | <0.001 |
| **Apolipoprotein B** |  |  |  |  |  |  |  |  |  |  |
| Quintile 1 | 836 | 2.79 | Reference | | 711 | 3.61 | Reference | | Reference | |
| Quintile 2 | 1181 | 3.06 | 0.96 (0.87, 1.06) | 0.422 | 354 | 3.11 | 0.84 (0.72, 0.98) | 0.027 | 0.89 (0.74, 1.06) | 0.200 |
| Quintile 3 | 1308 | 3.04 | 0.91 (0.83, 1.01) | 0.072 | 210 | 3.18 | 0.92 (0.76, 1.10) | 0.346 | 1.01 (0.82, 1.24) | 0.918 |
| Quintile 4 | 1365 | 2.96 | 0.86 (0.78, 0.95) | 0.003 | 127 | 3.34 | 1.02 (0.82, 1.27) | 0.852 | 1.19 (0.94, 1.50) | 0.150 |
| Quintile 5 | 1401 | 2.88 | 0.84 (0.76, 0.93) | <0.001 | 98 | 3.83 | 1.15 (0.91, 1.46) | 0.250 | 1.42 (1.10, 1.83) | 0.007 |
| Per 1-SD increase | NA | NA | 0.94 (0.91, 0.97) | <0.001 | NA | NA | 1.00 (0.94, 1.08) | 0.898 | 1.08 (1.00, 1.16) | 0.054 |
| **Total cholesterol** |  |  |  |  |  |  |  |  |  |  |
| Quintile 1 | 589 | 2.22 | Reference | | 693 | 2.89 | Reference | | Reference | |
| Quintile 2 | 995 | 2.52 | 0.94 (0.84, 1.06) | 0.311 | 370 | 3.58 | 0.99 (0.85, 1.15) | 0.880 | 1.06 (0.88, 1.28) | 0.557 |
| Quintile 3 | 1298 | 2.91 | 0.93 (0.83, 1.04) | 0.193 | 219 | 4.21 | 1.19 (0.99, 1.42) | 0.065 | 1.29 (1.04, 1.58) | 0.018 |
| Quintile 4 | 1493 | 3.16 | 0.88 (0.79, 0.99) | 0.029 | 124 | 4.58 | 1.24 (0.99, 1.55) | 0.055 | 1.41 (1.11, 1.80) | 0.006 |
| Quintile 5 | 1716 | 3.54 | 0.85 (0.76, 0.95) | 0.005 | 94 | 5.12 | 1.43 (1.12, 1.83) | 0.004 | 1.71 (1.32, 2.22) | <0.001 |
| Per 1-SD increase | NA | NA | 0.95 (0.92, 0.99) | 0.005 | NA | NA | 1.10 (1.03, 1.18) | 0.007 | 1.16 (1.08, 1.25) | <0.001 |
| **HDL cholesterol** |  |  |  |  |  |  |  |  |  |  |
| Quintile 1 | 538 | 1.46 | Reference | | 292 | 1.93 | Reference | | Reference | |
| Quintile 2 | 806 | 1.99 | 0.98 (0.86, 1.11) | 0.710 | 292 | 2.79 | 1.14 (0.93, 1.38) | 0.201 | 1.15 (0.92, 1.45) | 0.223 |
| Quintile 3 | 1078 | 2.56 | 1.01 (0.89, 1.14) | 0.859 | 277 | 3.44 | 1.20 (0.98, 1.47) | 0.081 | 1.16 (0.92, 1.45) | 0.212 |
| Quintile 4 | 1556 | 3.58 | 1.10 (0.97, 1.24) | 0.126 | 286 | 4.87 | 1.55 (1.26, 1.91) | <0.001 | 1.34 (1.07, 1.67) | 0.010 |
| Quintile 5 | 2113 | 4.89 | 1.17 (1.04, 1.33) | 0.012 | 353 | 7.84 | 1.83 (1.48, 2.28) | <0.001 | 1.46 (1.17, 1.80) | <0.001 |
| Per 1-SD increase | NA | NA | 1.06 (1.03, 1.09) | <0.001 | NA | NA | 1.22 (1.15, 1.30) | <0.001 | 1.12 (1.06, 1.19) | <0.001 |
| **LDL cholesterol** |  |  |  |  |  |  |  |  |  |  |
| Quintile 1 | 665 | 2.56 | Reference | | 800 | 3.33 | Reference | | Reference | |
| Quintile 2 | 1135 | 2.93 | 0.99 (0.89, 1.10) | 0.820 | 356 | 3.33 | 0.92 (0.79, 1.07) | 0.258 | 0.94 (0.78, 1.13) | 0.496 |
| Quintile 3 | 1354 | 3.03 | 0.97 (0.87, 1.08) | 0.543 | 171 | 3.40 | 0.96 (0.79, 1.17) | 0.717 | 1.02 (0.82, 1.26) | 0.889 |
| Quintile 4 | 1390 | 2.92 | 0.87 (0.78, 0.97) | 0.013 | 101 | 4.03 | 1.14 (0.89, 1.45) | 0.295 | 1.32 (1.02, 1.71) | 0.036 |
| Quintile 5 | 1547 | 3.15 | 0.88 (0.79, 0.98) | 0.022 | 72 | 4.00 | 1.23 (0.95, 1.61) | 0.122 | 1.43 (1.08, 1.90) | 0.013 |
| Per 1-SD increase | NA | NA | 0.94 (0.91, 0.97) | <0.001 | NA | NA | 1.03 (0.96, 1.11) | 0.354 | 1.11 (1.03, 1.19) | 0.009 |
| **Triglycerides** |  |  |  |  |  |  |  |  |  |  |
| Quintile 1 | 1443 | 3.33 | Reference | | 288 | 5.17 | Reference | | Reference | |
| Quintile 2 | 1455 | 3.44 | 0.95 (0.88, 1.03) | 0.252 | 277 | 3.80 | 0.72 (0.60, 0.88) | 0.001 | 0.78 (0.63, 0.96) | 0.019 |
| Quintile 3 | 1279 | 3.13 | 0.90 (0.82, 0.98) | 0.016 | 292 | 3.32 | 0.72 (0.59, 0.87) | <0.001 | 0.82 (0.67, 1.01) | 0.066 |
| Quintile 4 | 1140 | 2.82 | 0.88 (0.80, 0.96) | 0.007 | 328 | 3.16 | 0.70 (0.58, 0.85) | <0.001 | 0.83 (0.68, 1.02) | 0.081 |
| Quintile 5 | 774 | 1.97 | 0.75 (0.68, 0.84) | <0.001 | 315 | 2.62 | 0.66 (0.54, 0.81) | <0.001 | 0.90 (0.73, 1.11) | 0.338 |
| Per 1-SD increase | NA | NA | 0.90 (0.87, 0.94) | <0.001 | NA | NA | 0.92 (0.86, 0.98) | 0.013 | 1.01 (0.94, 1.09) | 0.694 |
| **Lipoprotein A** |  |  |  |  |  |  |  |  |  |  |
| Quintile 1 | 1176 | 2.81 | Reference | | 256 | 3.00 | Reference | | Reference | |
| Quintile 2 | 1174 | 2.75 | 0.96 (0.88, 1.05) | 0.383 | 228 | 2.91 | 0.97 (0.78, 1.19) | 0.748 | 1.01 (0.80, 1.26) | 0.944 |
| Quintile 3 | 1240 | 2.97 | 0.91 (0.83, 1.00) | 0.042 | 297 | 3.60 | 1.04 (0.86, 1.27) | 0.665 | 1.15 (0.93, 1.43) | 0.193 |
| Quintile 4 | 1291 | 3.16 | 1.00 (0.92, 1.10) | 0.962 | 305 | 3.61 | 1.10 (0.90, 1.34) | 0.339 | 1.09 (0.88, 1.35) | 0.406 |
| Quintile 5 | 1210 | 3.10 | 0.94 (0.86, 1.03) | 0.213 | 414 | 3.77 | 1.09 (0.90, 1.31) | 0.377 | 1.16 (0.95, 1.42) | 0.149 |
| Per 1-SD increase | NA | NA | 1.00 (0.97, 1.02) | 0.782 | NA | NA | 1.01 (0.96, 1.07) | 0.630 | 1.02 (0.96, 1.08) | 0.522 |

Notes: LLM = Lipid-lowering medications; HR = Hazard ratio; CI = Confidence interval; HDL = High density lipoprotein; LDL = Low density lipoprotein; NA= Not available. The unit of crude incidence rate: events per 1,000 person-years. Model was adjusted for age, sex, ethnicity, deprivation index, current smokers, alcohol intake, sleep duration, total physical activity, total sedentary behavior, fruit and vegetables intake, red meat intake, processed meat intake, oily fish intake, ever eats eggs, ever eats dairy, body-mass index, waist-hip ratio, body fat percentage, number of morbidities, prefrail/frail status, cardiovascular disease, hypertension, diabetes , chronic kidney disease, cancer, anemia, positive rheumatoid factor, vitamin D deficiency, history of fractures, falls in the last year, aspirin, glucocorticoids, vitamin D supplements, and calcium supplements.

# Supplementary Table 13. Associations between serum lipid concentrations and the risk of type-specific fractures in the UK Biobank.

| **Lipid traits** | **Major osteoporotic fracture** | | | | **Hip fracture** | | | | **Clinical vertebral fracture** | | | |
| --- | --- | --- | --- | --- | --- | --- | --- | --- | --- | --- | --- | --- |
|  | **Event** | **Crude incidence rate (95% CI)** | **HR (95% CI)** | **P-value** | **Event** | **Crude incidence rate (95% CI)** | **HR (95% CI)** | **P-value** | **Event** | **Crude incidence rate (95% CI)** | **HR (95% CI)** | **P-value** |
| **Apolipoprotein A** | | | | |  | | | |  | | | |
| Quintile 1 | 1,998 | 1.86 (1.78, 1.95) | Reference | | 694 | 0.64 (0.60, 0.69) | Reference | | 681 | 0.63 (0.58, 0.68) | Reference | |
| Quintile 2 | 2,076 | 1.97 (1.89, 2.06) | 0.94 (0.87, 1.01) | 0.070 | 673 | 0.63 (0.59, 0.68) | 0.92 (0.81, 1.04) | 0.174 | 615 | 0.58 (0.53, 0.63) | 0.96 (0.84, 1.08) | 0.480 |
| Quintile 3 | 2,509 | 2.39 (2.30, 2.49) | 1.04 (0.97, 1.12) | 0.253 | 808 | 0.76 (0.71, 0.82) | 1.03 (0.91, 1.16) | 0.676 | 655 | 0.62 (0.57, 0.67) | 1.03 (0.91, 1.17) | 0.646 |
| Quintile 4 | 2,884 | 2.78 (2.68, 2.88) | 1.08 (1.01, 1.16) | 0.028 | 834 | 0.79 (0.74, 0.85) | 0.94 (0.83, 1.06) | 0.319 | 750 | 0.71 (0.66, 0.77) | 1.15 (1.02, 1.31) | 0.028 |
| Quintile 5 | 3,429 | 3.35 (3.24, 3.47) | 1.12 (1.04, 1.21) | 0.002 | 1,007 | 0.97 (0.91, 1.03) | 0.96 (0.84, 1.09) | 0.529 | 784 | 0.75 (0.70, 0.81) | 1.16 (1.01, 1.33) | 0.031 |
| Per 1-SD increase | NA | NA | 1.06 (1.04, 1.08) | <0.001 | NA | NA | 1.01 (0.97, 1.05) | 0.699 | NA | NA | 1.07 (1.03, 1.12) | 0.002 |
| **Apolipoprotein B** | | | |  |  |  |  |  |  |  |  |  |
| Quintile 1 | 2,827 | 2.69 (2.60, 2.79) | Reference | | 971 | 0.92 (0.86, 0.97) | Reference | | 811 | 0.76 (0.71, 0.82) | Reference | |
| Quintile 2 | 2,636 | 2.51 (2.41, 2.60) | 0.92 (0.87, 0.98) | 0.007 | 833 | 0.78 (0.73, 0.84) | 0.91 (0.82, 1.02) | 0.099 | 711 | 0.67 (0.62, 0.72) | 0.90 (0.80, 1.01) | 0.064 |
| Quintile 3 | 2,509 | 2.40 (2.31, 2.49) | 0.89 (0.84, 0.95) | <0.001 | 753 | 0.71 (0.66, 0.77) | 0.82 (0.73, 0.92) | <0.001 | 664 | 0.63 (0.58, 0.68) | 0.89 (0.79, 1.00) | 0.054 |
| Quintile 4 | 2,476 | 2.37 (2.28, 2.47) | 0.86 (0.81, 0.92) | <0.001 | 731 | 0.69 (0.64, 0.75) | 0.79 (0.70, 0.88) | <0.001 | 664 | 0.63 (0.58, 0.68) | 0.90 (0.80, 1.02) | 0.098 |
| Quintile 5 | 2,448 | 2.35 (2.26, 2.44) | 0.82 (0.76, 0.87) | <0.001 | 728 | 0.69 (0.64, 0.74) | 0.76 (0.68, 0.86) | <0.001 | 635 | 0.60 (0.56, 0.65) | 0.79 (0.70, 0.90) | <0.001 |
| Per 1-SD increase | NA | NA | 0.92 (0.90, 0.95) | <0.001 | NA | NA | 0.89 (0.85, 0.93) | <0.001 | NA | NA | 0.93 (0.89, 0.98) | 0.004 |
| **Total cholesterol** | | |  |  |  |  |  |  |  |  |  |  |
| Quintile 1 | 2,558 | 2.42 (2.33, 2.52) | Reference | | 954 | 0.90 (0.84, 0.95) | Reference | | 789 | 0.74 (0.69, 0.79) | Reference | |
| Quintile 2 | 2,466 | 2.34 (2.25, 2.44) | 0.99 (0.93, 1.06) | 0.863 | 761 | 0.72 (0.67, 0.77) | 0.89 (0.79, 0.99) | 0.039 | 716 | 0.67 (0.63, 0.72) | 1.05 (0.93, 1.18) | 0.451 |
| Quintile 3 | 2,482 | 2.37 (2.28, 2.46) | 0.97 (0.91, 1.04) | 0.405 | 767 | 0.72 (0.67, 0.78) | 0.87 (0.77, 0.98) | 0.023 | 661 | 0.62 (0.58, 0.67) | 1.00 (0.88, 1.13) | 0.969 |
| Quintile 4 | 2,606 | 2.50 (2.40, 2.59) | 0.96 (0.89, 1.03) | 0.243 | 725 | 0.69 (0.64, 0.74) | 0.78 (0.69, 0.89) | <0.001 | 657 | 0.62 (0.58, 0.67) | 0.96 (0.84, 1.10) | 0.555 |
| Quintile 5 | 2,784 | 2.69 (2.59, 2.79) | 0.92 (0.86, 0.99) | 0.020 | 809 | 0.77 (0.72, 0.83) | 0.75 (0.66, 0.85) | <0.001 | 662 | 0.63 (0.58, 0.68) | 0.93 (0.81, 1.06) | 0.279 |
| Per 1-SD increase | NA | NA | 0.96 (0.93, 0.98) | 0.002 | NA | NA | 0.88 (0.84, 0.92) | <0.001 | NA | NA | 0.97 (0.92, 1.02) | 0.236 |
| **HDL cholesterol** | | |  |  |  |  |  |  |  |  |  |  |
| Quintile 1 | 1,980 | 1.85 (1.77, 1.93) | Reference | | 711 | 0.66 (0.61, 0.71) | Reference | | 674 | 0.63 (0.58, 0.67) | Reference | |
| Quintile 2 | 2,095 | 1.98 (1.90, 2.07) | 0.99 (0.92, 1.06) | 0.705 | 657 | 0.62 (0.57, 0.66) | 0.92 (0.81, 1.04) | 0.164 | 668 | 0.63 (0.58, 0.68) | 1.08 (0.95, 1.22) | 0.230 |
| Quintile 3 | 2,474 | 2.37 (2.28, 2.47) | 1.08 (1.00, 1.16) | 0.043 | 756 | 0.72 (0.67, 0.77) | 1.00 (0.89, 1.14) | 0.940 | 688 | 0.65 (0.60, 0.70) | 1.15 (1.01, 1.30) | 0.033 |
| Quintile 4 | 2,872 | 2.76 (2.66, 2.87) | 1.15 (1.07, 1.24) | <0.001 | 862 | 0.82 (0.77, 0.88) | 1.03 (0.91, 1.17) | 0.643 | 712 | 0.68 (0.63, 0.73) | 1.23 (1.08, 1.40) | 0.002 |
| Quintile 5 | 3,475 | 3.40 (3.29, 3.51) | 1.27 (1.17, 1.37) | <0.001 | 1,030 | 0.99 (0.93, 1.05) | 1.09 (0.96, 1.25) | 0.192 | 743 | 0.71 (0.66, 0.77) | 1.29 (1.12, 1.49) | <0.001 |
| Per 1-SD increase | NA | NA | 1.10 (1.07, 1.13) | <0.001 | NA | NA | 1.05 (1.00, 1.09) | 0.037 | NA | NA | 1.10 (1.05, 1.15) | <0.001 |
| **LDL cholesterol** | | |  |  |  |  |  |  |  |  |  |  |
| Quintile 1 | 2,767 | 2.64 (2.54, 2.74) | Reference | | 999 | 0.94 (0.88, 1.00) | Reference | | 832 | 0.78 (0.73, 0.84) | Reference | |
| Quintile 2 | 2,592 | 2.47 (2.38, 2.57) | 0.96 (0.90, 1.02) | 0.176 | 813 | 0.77 (0.72, 0.82) | 0.91 (0.81, 1.01) | 0.085 | 709 | 0.67 (0.62, 0.72) | 0.96 (0.85, 1.08) | 0.480 |
| Quintile 3 | 2,486 | 2.37 (2.28, 2.47) | 0.92 (0.86, 0.99) | 0.019 | 728 | 0.69 (0.64, 0.74) | 0.80 (0.71, 0.91) | <0.001 | 672 | 0.63 (0.59, 0.68) | 0.95 (0.84, 1.08) | 0.449 |
| Quintile 4 | 2,464 | 2.35 (2.26, 2.45) | 0.89 (0.83, 0.95) | <0.001 | 724 | 0.68 (0.64, 0.74) | 0.79 (0.70, 0.90) | <0.001 | 636 | 0.60 (0.56, 0.65) | 0.87 (0.76, 0.99) | 0.036 |
| Quintile 5 | 2,587 | 2.49 (2.39, 2.59) | 0.86 (0.81, 0.93) | <0.001 | 752 | 0.72 (0.67, 0.77) | 0.74 (0.65, 0.84) | <0.001 | 636 | 0.60 (0.56, 0.65) | 0.86 (0.75, 0.99) | 0.030 |
| Per 1-SD increase | NA | NA | 0.93 (0.91, 0.96) | <0.001 | NA | NA | 0.87 (0.83, 0.91) | <0.001 | NA | NA | 0.94 (0.90, 0.99) | 0.020 |
| **Triglycerides** |  |  |  |  |  |  |  |  |  |  |  |  |
| Quintile 1 | 2,830 | 2.70 (2.60, 2.80) | Reference | | 876 | 0.83 (0.77, 0.88) | Reference | | 653 | 0.61 (0.57, 0.66) | Reference | |
| Quintile 2 | 2,732 | 2.62 (2.53, 2.72) | 0.87 (0.82, 0.92) | <0.001 | 856 | 0.81 (0.76, 0.87) | 0.86 (0.77, 0.96) | 0.005 | 684 | 0.65 (0.60, 0.70) | 0.90 (0.79, 1.01) | 0.073 |
| Quintile 3 | 2,677 | 2.57 (2.47, 2.66) | 0.83 (0.78, 0.88) | <0.001 | 811 | 0.77 (0.72, 0.82) | 0.76 (0.68, 0.85) | <0.001 | 722 | 0.68 (0.63, 0.74) | 0.84 (0.74, 0.95) | 0.005 |
| Quintile 4 | 2,477 | 2.37 (2.27, 2.46) | 0.78 (0.73, 0.83) | <0.001 | 783 | 0.74 (0.69, 0.79) | 0.76 (0.67, 0.85) | <0.001 | 728 | 0.69 (0.64, 0.74) | 0.84 (0.74, 0.95) | 0.005 |
| Quintile 5 | 2,180 | 2.07 (1.99, 2.16) | 0.72 (0.67, 0.77) | <0.001 | 690 | 0.65 (0.60, 0.70) | 0.69 (0.61, 0.78) | <0.001 | 698 | 0.66 (0.61, 0.71) | 0.75 (0.66, 0.85) | <0.001 |
| Per 1-SD increase | NA | NA | 0.89 (0.87, 0.92) | <0.001 | NA | NA | 0.88 (0.84, 0.92) | <0.001 | NA | NA | 0.93 (0.89, 0.97) | <0.001 |
| **Lipoprotein A** | | |  |  |  |  |  |  |  |  |  |  |
| Quintile 1 | 2,500 | 2.38 (2.28, 2.47) | Reference | | 810 | 0.76 (0.71, 0.82) | Reference | | 684 | 0.64 (0.60, 0.69) | Reference | |
| Quintile 2 | 2,530 | 2.41 (2.32, 2.51) | 1.02 (0.96, 1.09) | 0.450 | 758 | 0.72 (0.67, 0.77) | 0.97 (0.87, 1.08) | 0.562 | 712 | 0.67 (0.62, 0.72) | 1.04 (0.93, 1.17) | 0.511 |
| Quintile 3 | 2,591 | 2.48 (2.39, 2.58) | 0.99 (0.93, 1.05) | 0.669 | 794 | 0.75 (0.70, 0.81) | 0.95 (0.85, 1.06) | 0.365 | 693 | 0.66 (0.61, 0.71) | 0.99 (0.88, 1.12) | 0.920 |
| Quintile 4 | 2,615 | 2.50 (2.41, 2.60) | 1.00 (0.94, 1.07) | 0.948 | 807 | 0.76 (0.71, 0.82) | 0.96 (0.86, 1.08) | 0.519 | 700 | 0.66 (0.61, 0.71) | 1.01 (0.90, 1.13) | 0.887 |
| Quintile 5 | 2,660 | 2.55 (2.45, 2.65) | 0.98 (0.92, 1.04) | 0.567 | 847 | 0.80 (0.75, 0.86) | 0.96 (0.86, 1.07) | 0.446 | 696 | 0.66 (0.61, 0.71) | 0.97 (0.86, 1.09) | 0.598 |
| Per 1-SD increase | NA | NA | 0.99 (0.97, 1.01) | 0.533 | NA | NA | 0.99 (0.96, 1.03) | 0.776 | NA | NA | 0.98 (0.95, 1.02) | 0.429 |

Notes: HR = Hazard ratio; CI = Confidence interval; HDL = High density lipoprotein; LDL = Low density lipoprotein; NA= Not available. Model was adjusted for age, sex, ethnicity, deprivation index, current smokers, alcohol intake, sleep duration, total physical activity, total sedentary behavior, fruit and vegetables intake, red meat intake, processed meat intake, oily fish intake, ever eats eggs, ever eats dairy, body-mass index, waist-hip ratio, body fat percentage, number of morbidities, prefrail/frail status, cardiovascular disease, hypertension, diabetes , chronic kidney disease, cancer, anemia, positive rheumatoid factor, vitamin D deficiency, osteoporosis, falls in the last year, lipid-lowering medications, aspirin, glucocorticoids, vitamin D supplements, and calcium supplements.

# Supplementary Table 14. Associations between serum lipid concentrations and the risk of major osteoporotic fractures in the UK Biobank, stratified by age or sex.

| **Lipid traits** | **Age ≥ 60 years (ref)** | | **Age < 60 years** | | **Ratio of HR  (95% CI)** | **P-value** | **Women (ref)** | | **Men** | | **Ratio of HR  (95% CI)** | **P-value** |
| --- | --- | --- | --- | --- | --- | --- | --- | --- | --- | --- | --- | --- |
|  | **HR (95% CI)** | **P-value** | **HR (95% CI)** | **P-value** |  |  | **HR (95% CI)** | **P-value** | **HR (95% CI)** | **P-value** |  |  |
| **Apolipoprotein A** | |  |  |  |  |  |  |  |  |  |  |  |
| Quintile 1 | Reference | | Reference | | Reference | | Reference | | Reference | | Reference | |
| Quintile 2 | 0.92 (0.84, 1.01) | 0.072 | 1.00 (0.89, 1.13) | 0.957 | 1.03 (0.90, 1.19) | 0.624 | 0.93 (0.84, 1.04) | 0.207 | 0.95 (0.86, 1.05) | 0.299 | 0.97 (0.85, 1.11) | 0.644 |
| Quintile 3 | 0.97 (0.89, 1.07) | 0.575 | 1.24 (1.11, 1.39) | <0.001 | 1.10 (0.97, 1.25) | 0.144 | 1.02 (0.92, 1.12) | 0.751 | 1.08 (0.98, 1.20) | 0.133 | 0.93 (0.82, 1.07) | 0.318 |
| Quintile 4 | 1.02 (0.93, 1.11) | 0.718 | 1.35 (1.20, 1.51) | <0.001 | 1.13 (0.99, 1.28) | 0.062 | 1.06 (0.96, 1.17) | 0.278 | 1.09 (0.97, 1.22) | 0.128 | 0.97 (0.85, 1.11) | 0.700 |
| Quintile 5 | 1.06 (0.96, 1.16) | 0.251 | 1.49 (1.33, 1.67) | <0.001 | 1.18 (1.04, 1.33) | 0.008 | 1.05 (0.95, 1.16) | 0.312 | 1.33 (1.17, 1.51) | <0.001 | 1.11 (0.97, 1.28) | 0.137 |
| Per 1-SD increase | 1.04 (1.01, 1.07) | 0.007 | 1.16 (1.12, 1.20) | <0.001 | 1.06 (1.02, 1.10) | 0.001 | 1.04 (1.01, 1.06) | 0.008 | 1.11 (1.06, 1.16) | <0.001 | 1.04 (0.99, 1.08) | 0.099 |
| **Apolipoprotein B** | |  |  |  |  |  |  |  |  |  |  |  |
| Quintile 1 | Reference | | Reference | | Reference | | Reference | | Reference | | Reference | |
| Quintile 2 | 0.92 (0.85, 0.99) | 0.031 | 0.98 (0.89, 1.08) | 0.661 | 1.03 (0.92, 1.16) | 0.576 | 0.92 (0.85, 0.99) | 0.023 | 0.94 (0.84, 1.04) | 0.201 | 1.02 (0.91, 1.15) | 0.721 |
| Quintile 3 | 0.86 (0.79, 0.93) | <0.001 | 1.05 (0.95, 1.16) | 0.328 | 1.18 (1.05, 1.33) | 0.006 | 0.91 (0.84, 0.98) | 0.013 | 0.87 (0.78, 0.97) | 0.014 | 0.98 (0.87, 1.11) | 0.802 |
| Quintile 4 | 0.85 (0.79, 0.93) | <0.001 | 1.01 (0.91, 1.12) | 0.876 | 1.13 (1.00, 1.27) | 0.048 | 0.89 (0.82, 0.96) | 0.003 | 0.82 (0.73, 0.92) | <0.001 | 0.93 (0.82, 1.05) | 0.228 |
| Quintile 5 | 0.82 (0.75, 0.89) | <0.001 | 0.96 (0.86, 1.07) | 0.429 | 1.12 (0.99, 1.27) | 0.068 | 0.81 (0.74, 0.88) | <0.001 | 0.85 (0.76, 0.96) | 0.007 | 1.05 (0.92, 1.19) | 0.466 |
| Per 1-SD increase | 0.94 (0.91, 0.96) | <0.001 | 0.99 (0.96, 1.03) | 0.642 | 1.04 (1.00, 1.09) | 0.029 | 0.94 (0.91, 0.96) | <0.001 | 0.94 (0.90, 0.97) | 0.001 | 0.99 (0.95, 1.03) | 0.732 |
| **Total cholesterol** | |  |  |  |  |  |  |  |  |  |  |  |
| Quintile 1 | Reference | | Reference | | Reference | | Reference | | Reference | | Reference | |
| Quintile 2 | 0.99 (0.91, 1.08) | 0.855 | 1.06 (0.95, 1.18) | 0.284 | 0.98 (0.87, 1.11) | 0.808 | 1.03 (0.95, 1.13) | 0.446 | 0.95 (0.86, 1.05) | 0.306 | 0.94 (0.84, 1.06) | 0.339 |
| Quintile 3 | 0.94 (0.86, 1.03) | 0.167 | 1.15 (1.04, 1.28) | 0.008 | 1.13 (0.99, 1.27) | 0.061 | 1.02 (0.93, 1.11) | 0.658 | 0.90 (0.81, 1.01) | 0.071 | 0.92 (0.82, 1.04) | 0.199 |
| Quintile 4 | 0.93 (0.85, 1.02) | 0.133 | 1.18 (1.06, 1.32) | 0.002 | 1.13 (1.00, 1.28) | 0.054 | 1.01 (0.92, 1.10) | 0.895 | 0.89 (0.79, 1.00) | 0.059 | 0.89 (0.78, 1.00) | 0.058 |
| Quintile 5 | 0.88 (0.80, 0.97) | 0.008 | 1.22 (1.09, 1.36) | <0.001 | 1.23 (1.09, 1.39) | 0.001 | 0.94 (0.86, 1.03) | 0.198 | 0.91 (0.81, 1.03) | 0.155 | 0.98 (0.86, 1.11) | 0.746 |
| Per 1-SD increase | 0.95 (0.93, 0.98) | 0.002 | 1.07 (1.03, 1.10) | <0.001 | 1.07 (1.03, 1.11) | <0.001 | 0.97 (0.94, 0.99) | 0.012 | 0.96 (0.92, 1.00) | 0.071 | 0.99 (0.95, 1.03) | 0.580 |
| **HDL cholesterol** | |  |  |  |  |  |  |  |  |  |  |  |
| Quintile 1 | Reference | | Reference | | Reference | | Reference | | Reference | | Reference | |
| Quintile 2 | 0.98 (0.90, 1.07) | 0.681 | 1.04 (0.93, 1.17) | 0.496 | 0.98 (0.85, 1.12) | 0.752 | 0.95 (0.85, 1.07) | 0.395 | 1.01 (0.92, 1.12) | 0.769 | 1.00 (0.87, 1.15) | 0.983 |
| Quintile 3 | 1.04 (0.95, 1.14) | 0.399 | 1.24 (1.10, 1.39) | <0.001 | 1.05 (0.92, 1.20) | 0.471 | 1.02 (0.92, 1.14) | 0.685 | 1.13 (1.02, 1.25) | 0.021 | 1.03 (0.90, 1.17) | 0.712 |
| Quintile 4 | 1.10 (1.01, 1.21) | 0.034 | 1.38 (1.22, 1.55) | <0.001 | 1.02 (0.90, 1.16) | 0.760 | 1.09 (0.98, 1.21) | 0.106 | 1.18 (1.05, 1.33) | 0.004 | 1.00 (0.87, 1.15) | 0.994 |
| Quintile 5 | 1.22 (1.11, 1.34) | <0.001 | 1.61 (1.42, 1.83) | <0.001 | 1.06 (0.94, 1.20) | 0.339 | 1.16 (1.05, 1.29) | 0.005 | 1.46 (1.28, 1.67) | <0.001 | 1.18 (1.02, 1.36) | 0.022 |
| Per 1-SD increase | 1.08 (1.05, 1.11) | <0.001 | 1.19 (1.15, 1.24) | <0.001 | 1.04 (1.00, 1.07) | 0.051 | 1.08 (1.05, 1.11) | <0.001 | 1.13 (1.08, 1.18) | <0.001 | 1.04 (0.99, 1.08) | 0.090 |
| **LDL cholesterol** | |  |  |  |  |  |  |  |  |  |  |  |
| Quintile 1 | Reference | | Reference | | Reference | | Reference | | Reference | | Reference | |
| Quintile 2 | 0.95 (0.87, 1.03) | 0.203 | 1.03 (0.93, 1.14) | 0.545 | 1.01 (0.90, 1.14) | 0.806 | 0.96 (0.88, 1.04) | 0.272 | 0.96 (0.87, 1.07) | 0.491 | 1.03 (0.92, 1.16) | 0.588 |
| Quintile 3 | 0.90 (0.83, 0.98) | 0.019 | 1.07 (0.96, 1.18) | 0.222 | 1.08 (0.95, 1.21) | 0.236 | 0.94 (0.87, 1.03) | 0.171 | 0.89 (0.79, 0.99) | 0.035 | 0.97 (0.86, 1.09) | 0.617 |
| Quintile 4 | 0.84 (0.77, 0.92) | <0.001 | 1.12 (1.01, 1.25) | 0.032 | 1.22 (1.08, 1.37) | 0.001 | 0.91 (0.84, 0.99) | 0.032 | 0.86 (0.76, 0.97) | 0.012 | 0.95 (0.84, 1.07) | 0.379 |
| Quintile 5 | 0.85 (0.78, 0.93) | <0.001 | 1.07 (0.95, 1.19) | 0.259 | 1.13 (1.00, 1.28) | 0.054 | 0.86 (0.79, 0.94) | <0.001 | 0.88 (0.78, 1.00) | 0.046 | 1.01 (0.89, 1.15) | 0.826 |
| Per 1-SD increase | 0.94 (0.91, 0.97) | <0.001 | 1.02 (0.99, 1.06) | 0.257 | 1.05 (1.01, 1.09) | 0.010 | 0.95 (0.92, 0.97) | <0.001 | 0.94 (0.90, 0.98) | 0.002 | 0.99 (0.95, 1.03) | 0.462 |
| **Triglycerides** | |  |  |  |  |  |  |  |  |  |  |  |
| Quintile 1 | Reference | | Reference | | Reference | | Reference | | Reference | | Reference | |
| Quintile 2 | 0.86 (0.79, 0.93) | <0.001 | 0.95 (0.86, 1.04) | 0.237 | 1.11 (0.99, 1.24) | 0.076 | 0.85 (0.80, 0.91) | <0.001 | 0.94 (0.83, 1.05) | 0.264 | 0.99 (0.87, 1.12) | 0.837 |
| Quintile 3 | 0.80 (0.74, 0.87) | <0.001 | 0.96 (0.87, 1.06) | 0.387 | 1.26 (1.12, 1.41) | <0.001 | 0.82 (0.76, 0.88) | <0.001 | 0.87 (0.78, 0.98) | 0.020 | 0.97 (0.85, 1.09) | 0.581 |
| Quintile 4 | 0.75 (0.69, 0.81) | <0.001 | 0.92 (0.83, 1.03) | 0.145 | 1.30 (1.16, 1.47) | <0.001 | 0.75 (0.69, 0.81) | <0.001 | 0.87 (0.78, 0.98) | 0.019 | 1.04 (0.92, 1.18) | 0.517 |
| Quintile 5 | 0.69 (0.63, 0.75) | <0.001 | 0.83 (0.74, 0.93) | 0.002 | 1.26 (1.12, 1.43) | <0.001 | 0.68 (0.62, 0.75) | <0.001 | 0.81 (0.72, 0.91) | <0.001 | 1.05 (0.93, 1.20) | 0.414 |
| Per 1-SD increase | 0.88 (0.86, 0.91) | <0.001 | 0.94 (0.91, 0.98) | 0.002 | 1.09 (1.04, 1.13) | <0.001 | 0.87 (0.84, 0.90) | <0.001 | 0.94 (0.90, 0.97) | <0.001 | 1.04 (1.00, 1.09) | 0.062 |
| **Lipoprotein A** | |  |  |  |  |  |  |  |  |  |  |  |
| Quintile 1 | Reference | | Reference | | Reference | | Reference | | Reference | | Reference | |
| Quintile 2 | 1.01 (0.94, 1.10) | 0.737 | 1.05 (0.95, 1.15) | 0.349 | 1.03 (0.91, 1.16) | 0.662 | 1.00 (0.92, 1.08) | 0.956 | 1.07 (0.97, 1.19) | 0.180 | 1.06 (0.94, 1.20) | 0.347 |
| Quintile 3 | 0.97 (0.90, 1.05) | 0.472 | 1.05 (0.95, 1.16) | 0.348 | 1.05 (0.93, 1.18) | 0.458 | 0.98 (0.91, 1.05) | 0.561 | 1.00 (0.90, 1.12) | 0.963 | 1.02 (0.90, 1.16) | 0.737 |
| Quintile 4 | 1.03 (0.95, 1.11) | 0.514 | 0.99 (0.90, 1.10) | 0.863 | 0.92 (0.81, 1.04) | 0.165 | 0.99 (0.92, 1.07) | 0.865 | 1.01 (0.91, 1.13) | 0.790 | 1.01 (0.89, 1.14) | 0.922 |
| Quintile 5 | 0.99 (0.92, 1.07) | 0.816 | 1.00 (0.90, 1.10) | 0.976 | 0.99 (0.88, 1.11) | 0.838 | 0.99 (0.91, 1.06) | 0.705 | 0.97 (0.87, 1.08) | 0.537 | 0.95 (0.84, 1.07) | 0.402 |
| Per 1-SD increase | 1.00 (0.97, 1.02) | 0.737 | 0.99 (0.96, 1.02) | 0.571 | 0.99 (0.96, 1.03) | 0.654 | 0.99 (0.97, 1.02) | 0.658 | 0.98 (0.95, 1.02) | 0.282 | 0.98 (0.94, 1.02) | 0.242 |

Notes: HR = Hazard ratio; CI = Confidence interval; HDL = High density lipoprotein; LDL = Low density lipoprotein. Model was adjusted for age (excluded in the model stratified by age), sex (excluded in the model stratified by sex), ethnicity, deprivation index, current smokers, alcohol intake, sleep duration, total physical activity, total sedentary behavior, fruit and vegetables intake, red meat intake, processed meat intake, oily fish intake, ever eats eggs, ever eats dairy, body-mass index, waist-hip ratio, body fat percentage, number of morbidities, prefrail/frail status, cardiovascular disease, hypertension, diabetes , chronic kidney disease, cancer, anemia, positive rheumatoid factor, vitamin D deficiency, osteoporosis, falls in the last year, menopause status (included in the model for women), lipid-lowering medications, aspirin, glucocorticoids, vitamin D supplements, and calcium supplements.

# Supplementary Table 15. Associations between serum lipid concentrations and the risk of major osteoporotic fractures in the UK Biobank, stratified by BMI.

| **Lipid traits** | **Normal weight (ref)** | | **Overweight** | | **Ratio of HR  (95% CI)** | **P-value** | **Obese** | | **Ratio of HR  (95% CI)** | **P-value** |
| --- | --- | --- | --- | --- | --- | --- | --- | --- | --- | --- |
|  | **HR (95% CI)** | **P-value** | **HR (95% CI)** | **P-value** |  |  | **HR (95% CI)** | **P-value** |  |  |
| **Apolipoprotein A** |  |  |  |  |  |  |  |  |  |  |
| Quintile 1 | Reference | | Reference | | Reference | | Reference | | Reference | |
| Quintile 2 | 0.94 (0.81, 1.09) | 0.417 | 0.95 (0.85, 1.06) | 0.389 | 1.04 (0.87, 1.23) | 0.692 | 0.91 (0.80, 1.04) | 0.155 | 0.96 (0.80, 1.15) | 0.660 |
| Quintile 3 | 1.05 (0.91, 1.20) | 0.525 | 1.08 (0.97, 1.20) | 0.175 | 1.06 (0.91, 1.25) | 0.452 | 0.99 (0.87, 1.13) | 0.896 | 0.94 (0.79, 1.12) | 0.478 |
| Quintile 4 | 1.05 (0.91, 1.20) | 0.511 | 1.13 (1.01, 1.26) | 0.028 | 1.11 (0.95, 1.29) | 0.186 | 1.05 (0.91, 1.20) | 0.520 | 0.97 (0.82, 1.15) | 0.731 |
| Quintile 5 | 1.06 (0.93, 1.21) | 0.403 | 1.18 (1.05, 1.32) | 0.005 | 1.10 (0.95, 1.27) | 0.221 | 1.12 (0.96, 1.31) | 0.148 | 0.99 (0.83, 1.17) | 0.868 |
| Per 1-SD increase | 1.03 (1.00, 1.07) | 0.048 | 1.06 (1.02, 1.10) | 0.001 | 1.02 (0.98, 1.06) | 0.280 | 1.07 (1.02, 1.13) | 0.009 | 1.01 (0.96, 1.06) | 0.804 |
| **Apolipoprotein B** |  |  |  |  |  |  |  |  |  |  |
| Quintile 1 | Reference | | Reference | | Reference | | Reference | | Reference | |
| Quintile 2 | 0.92 (0.84, 1.01) | 0.099 | 0.93 (0.84, 1.03) | 0.148 | 1.04 (0.91, 1.18) | 0.596 | 0.88 (0.77, 1.00) | 0.058 | 0.96 (0.82, 1.11) | 0.570 |
| Quintile 3 | 0.86 (0.78, 0.95) | 0.004 | 0.95 (0.85, 1.05) | 0.294 | 1.13 (0.99, 1.28) | 0.066 | 0.88 (0.77, 1.02) | 0.092 | 1.04 (0.89, 1.21) | 0.624 |
| Quintile 4 | 0.90 (0.81, 0.99) | 0.035 | 0.85 (0.77, 0.95) | 0.004 | 1.02 (0.90, 1.16) | 0.747 | 0.87 (0.75, 1.01) | 0.067 | 1.04 (0.89, 1.21) | 0.643 |
| Quintile 5 | 0.82 (0.73, 0.91) | <0.001 | 0.81 (0.73, 0.91) | <0.001 | 1.03 (0.91, 1.18) | 0.617 | 0.87 (0.75, 1.00) | 0.054 | 1.08 (0.92, 1.26) | 0.346 |
| Per 1-SD increase | 0.94 (0.90, 0.97) | <0.001 | 0.93 (0.90, 0.97) | <0.001 | 1.01 (0.97, 1.06) | 0.496 | 0.96 (0.92, 1.01) | 0.105 | 1.04 (0.99, 1.09) | 0.132 |
| **Total cholesterol** |  |  |  |  |  |  |  |  |  |  |
| Quintile 1 | Reference | | Reference | | Reference | | Reference | | Reference | |
| Quintile 2 | 0.99 (0.88, 1.10) | 0.802 | 1.04 (0.93, 1.16) | 0.488 | 1.06 (0.92, 1.21) | 0.446 | 0.93 (0.81, 1.06) | 0.258 | 0.96 (0.82, 1.12) | 0.622 |
| Quintile 3 | 1.01 (0.90, 1.12) | 0.928 | 0.99 (0.89, 1.11) | 0.902 | 1.04 (0.90, 1.19) | 0.616 | 0.89 (0.77, 1.03) | 0.108 | 0.91 (0.78, 1.07) | 0.251 |
| Quintile 4 | 0.95 (0.85, 1.07) | 0.387 | 1.01 (0.90, 1.13) | 0.840 | 1.07 (0.94, 1.23) | 0.298 | 0.92 (0.79, 1.07) | 0.300 | 0.98 (0.84, 1.15) | 0.807 |
| Quintile 5 | 0.96 (0.85, 1.08) | 0.460 | 0.90 (0.80, 1.01) | 0.062 | 0.99 (0.86, 1.13) | 0.849 | 0.93 (0.80, 1.08) | 0.338 | 1.02 (0.87, 1.18) | 0.847 |
| Per 1-SD increase | 0.97 (0.93, 1.01) | 0.123 | 0.96 (0.92, 0.99) | 0.021 | 1.00 (0.96, 1.05) | 0.862 | 0.98 (0.93, 1.03) | 0.339 | 1.01 (0.96, 1.06) | 0.632 |
| **HDL cholesterol** |  |  |  |  |  |  |  |  |  |  |
| Quintile 1 | Reference | | Reference | | Reference | | Reference | | Reference | |
| Quintile 2 | 0.96 (0.82, 1.13) | 0.633 | 1.02 (0.91, 1.13) | 0.744 | 1.08 (0.90, 1.30) | 0.397 | 0.97 (0.86, 1.10) | 0.660 | 1.04 (0.86, 1.25) | 0.690 |
| Quintile 3 | 1.03 (0.88, 1.19) | 0.741 | 1.14 (1.02, 1.27) | 0.021 | 1.12 (0.95, 1.33) | 0.183 | 1.04 (0.91, 1.19) | 0.545 | 1.00 (0.84, 1.20) | 0.981 |
| Quintile 4 | 1.10 (0.95, 1.28) | 0.192 | 1.21 (1.08, 1.35) | <0.001 | 1.12 (0.95, 1.32) | 0.167 | 1.11 (0.96, 1.28) | 0.158 | 0.97 (0.81, 1.16) | 0.750 |
| Quintile 5 | 1.18 (1.02, 1.37) | 0.027 | 1.30 (1.15, 1.46) | <0.001 | 1.08 (0.92, 1.26) | 0.353 | 1.33 (1.13, 1.58) | <0.001 | 1.03 (0.85, 1.24) | 0.764 |
| Per 1-SD increase | 1.07 (1.03, 1.10) | <0.001 | 1.10 (1.06, 1.14) | <0.001 | 1.02 (0.98, 1.07) | 0.257 | 1.14 (1.08, 1.21) | <0.001 | 1.02 (0.97, 1.07) | 0.516 |
| **LDL cholesterol** |  |  |  |  |  |  |  |  |  |  |
| Quintile 1 | Reference | | Reference | | Reference | | Reference | | Reference | |
| Quintile 2 | 0.95 (0.86, 1.06) | 0.364 | 0.97 (0.87, 1.08) | 0.597 | 1.04 (0.91, 1.19) | 0.533 | 0.97 (0.85, 1.11) | 0.672 | 1.02 (0.88, 1.19) | 0.787 |
| Quintile 3 | 0.94 (0.85, 1.04) | 0.246 | 0.95 (0.85, 1.06) | 0.360 | 1.04 (0.91, 1.18) | 0.572 | 0.89 (0.76, 1.03) | 0.115 | 0.95 (0.82, 1.11) | 0.540 |
| Quintile 4 | 0.92 (0.83, 1.03) | 0.137 | 0.90 (0.80, 1.01) | 0.062 | 1.02 (0.90, 1.16) | 0.753 | 0.89 (0.76, 1.03) | 0.124 | 1.01 (0.86, 1.17) | 0.940 |
| Quintile 5 | 0.88 (0.78, 0.99) | 0.028 | 0.86 (0.77, 0.96) | 0.010 | 1.02 (0.90, 1.17) | 0.716 | 0.91 (0.78, 1.06) | 0.230 | 1.08 (0.92, 1.25) | 0.348 |
| Per 1-SD increase | 0.95 (0.92, 0.99) | 0.008 | 0.94 (0.91, 0.97) | <0.001 | 1.01 (0.96, 1.05) | 0.771 | 0.96 (0.91, 1.01) | 0.090 | 1.02 (0.97, 1.07) | 0.439 |
| **Triglycerides** |  |  |  |  |  |  |  |  |  |  |
| Quintile 1 | Reference | | Reference | | Reference | | Reference | | Reference | |
| Quintile 2 | 0.88 (0.81, 0.96) | 0.003 | 0.87 (0.79, 0.96) | 0.007 | 1.00 (0.89, 1.13) | 0.943 | 0.86 (0.72, 1.03) | 0.106 | 1.06 (0.88, 1.27) | 0.541 |
| Quintile 3 | 0.84 (0.76, 0.92) | <0.001 | 0.82 (0.74, 0.91) | <0.001 | 0.98 (0.87, 1.11) | 0.780 | 0.84 (0.71, 1.00) | 0.049 | 1.07 (0.90, 1.28) | 0.448 |
| Quintile 4 | 0.82 (0.74, 0.92) | <0.001 | 0.75 (0.68, 0.83) | <0.001 | 0.95 (0.84, 1.09) | 0.486 | 0.79 (0.67, 0.94) | 0.006 | 1.06 (0.89, 1.28) | 0.501 |
| Quintile 5 | 0.77 (0.67, 0.87) | <0.001 | 0.70 (0.63, 0.78) | <0.001 | 0.93 (0.80, 1.09) | 0.382 | 0.71 (0.60, 0.85) | <0.001 | 1.06 (0.87, 1.28) | 0.568 |
| Per 1-SD increase | 0.89 (0.84, 0.93) | <0.001 | 0.90 (0.87, 0.94) | <0.001 | 1.03 (0.97, 1.09) | 0.291 | 0.91 (0.87, 0.95) | <0.001 | 1.07 (1.01, 1.14) | 0.027 |
| **Lipoprotein A** |  |  |  |  |  |  |  |  |  |  |
| Quintile 1 | Reference | | Reference | | Reference | | Reference | | Reference | |
| Quintile 2 | 1.01 (0.91, 1.11) | 0.852 | 1.02 (0.92, 1.12) | 0.710 | 1.00 (0.88, 1.15) | 0.944 | 1.04 (0.91, 1.19) | 0.593 | 1.01 (0.87, 1.19) | 0.864 |
| Quintile 3 | 1.00 (0.91, 1.10) | 1.000 | 0.95 (0.86, 1.05) | 0.281 | 0.97 (0.85, 1.11) | 0.654 | 1.04 (0.91, 1.20) | 0.522 | 1.08 (0.92, 1.26) | 0.351 |
| Quintile 4 | 0.94 (0.85, 1.04) | 0.224 | 1.04 (0.94, 1.15) | 0.427 | 1.11 (0.97, 1.26) | 0.126 | 1.06 (0.93, 1.22) | 0.369 | 1.11 (0.95, 1.30) | 0.196 |
| Quintile 5 | 1.00 (0.90, 1.10) | 0.969 | 0.92 (0.83, 1.01) | 0.086 | 0.96 (0.84, 1.10) | 0.554 | 1.08 (0.94, 1.23) | 0.269 | 1.07 (0.92, 1.25) | 0.362 |
| Per 1-SD increase | 0.99 (0.96, 1.03) | 0.732 | 0.97 (0.94, 1.00) | 0.065 | 1.00 (0.96, 1.04) | 0.841 | 1.02 (0.98, 1.07) | 0.284 | 1.03 (0.98, 1.08) | 0.252 |

Notes: HR = Hazard ratio; CI = Confidence interval; HDL = High density lipoprotein; LDL = Low density lipoprotein. Model was adjusted for age, sex, ethnicity, deprivation index, current smokers, alcohol intake, sleep duration, total physical activity, total sedentary behavior, fruit and vegetables intake, red meat intake, processed meat intake, oily fish intake, ever eats eggs, ever eats dairy, waist-hip ratio, body fat percentage, number of morbidities, prefrail/frail status, cardiovascular disease, hypertension, diabetes , chronic kidney disease, cancer, anemia, positive rheumatoid factor, vitamin D deficiency, osteoporosis, falls in the last year, lipid-lowering medications, aspirin, glucocorticoids, vitamin D supplements, and calcium supplements.

# Supplementary Table 16. Associations between serum lipid concentrations and the risk of major osteoporotic fractures in the UK Biobank, stratified by CVD or use of lipid-lowering medications.

| **Lipid traits** | **Non-CVD (ref)** | | **CVD** | | **Ratio of HR  (95% CI)** | **P-value** | **Not taking LLM (ref)** | | **Taking LLM** | | **Ratio of HR  (95% CI)** | **P-value** |
| --- | --- | --- | --- | --- | --- | --- | --- | --- | --- | --- | --- | --- |
|  | **HR (95% CI)** | **P-value** | **HR (95% CI)** | **P-value** |  |  | **HR (95% CI)** | **P-value** | **HR (95% CI)** | **P-value** |  |  |
| **Apolipoprotein A** | |  |  |  |  |  |  |  |  |  |  |  |
| Quintile 1 | Reference | | Reference | | Reference | | Reference | | Reference | | Reference | |
| Quintile 2 | 0.93 (0.86, 1.01) | 0.070 | 0.96 (0.79, 1.17) | 0.682 | 1.02 (0.84, 1.23) | 0.870 | 0.92 (0.84, 1.00) | 0.042 | 0.99 (0.86, 1.13) | 0.842 | 1.08 (0.93, 1.25) | 0.322 |
| Quintile 3 | 1.03 (0.95, 1.11) | 0.462 | 1.15 (0.95, 1.41) | 0.156 | 1.07 (0.89, 1.30) | 0.458 | 1.04 (0.96, 1.12) | 0.392 | 1.04 (0.90, 1.20) | 0.587 | 1.01 (0.87, 1.17) | 0.876 |
| Quintile 4 | 1.08 (1.00, 1.16) | 0.051 | 1.09 (0.87, 1.35) | 0.457 | 1.02 (0.84, 1.25) | 0.810 | 1.05 (0.97, 1.14) | 0.201 | 1.17 (1.01, 1.36) | 0.031 | 1.11 (0.96, 1.28) | 0.170 |
| Quintile 5 | 1.12 (1.04, 1.21) | 0.004 | 1.11 (0.88, 1.40) | 0.387 | 1.00 (0.82, 1.22) | 0.993 | 1.11 (1.02, 1.20) | 0.015 | 1.15 (0.98, 1.34) | 0.082 | 1.02 (0.88, 1.18) | 0.801 |
| Per 1-SD increase | 1.06 (1.03, 1.08) | <0.001 | 1.06 (0.98, 1.14) | 0.161 | 1.00 (0.94, 1.06) | 0.996 | 1.05 (1.03, 1.08) | <0.001 | 1.08 (1.03, 1.14) | 0.001 | 1.02 (0.98, 1.07) | 0.358 |
| **Apolipoprotein B** | |  |  |  |  |  |  |  |  |  |  |  |
| Quintile 1 | Reference | | Reference | | Reference | | Reference | | Reference | | Reference | |
| Quintile 2 | 0.89 (0.84, 0.95) | <0.001 | 1.09 (0.92, 1.28) | 0.316 | 1.27 (1.08, 1.50) | 0.004 | 0.93 (0.86, 1.00) | 0.049 | 0.86 (0.77, 0.96) | 0.008 | 0.96 (0.84, 1.08) | 0.465 |
| Quintile 3 | 0.87 (0.82, 0.93) | <0.001 | 1.00 (0.82, 1.23) | 0.969 | 1.24 (1.02, 1.50) | 0.031 | 0.86 (0.80, 0.93) | <0.001 | 0.99 (0.87, 1.12) | 0.874 | 1.15 (1.00, 1.32) | 0.049 |
| Quintile 4 | 0.85 (0.79, 0.91) | <0.001 | 0.87 (0.68, 1.11) | 0.268 | 1.19 (0.95, 1.49) | 0.124 | 0.84 (0.78, 0.91) | <0.001 | 0.90 (0.76, 1.06) | 0.198 | 1.13 (0.96, 1.33) | 0.145 |
| Quintile 5 | 0.80 (0.74, 0.86) | <0.001 | 0.94 (0.72, 1.23) | 0.651 | 1.42 (1.12, 1.79) | 0.003 | 0.79 (0.73, 0.85) | <0.001 | 1.07 (0.89, 1.29) | 0.445 | 1.42 (1.18, 1.70) | <0.001 |
| Per 1-SD increase | 0.93 (0.91, 0.96) | <0.001 | 0.96 (0.89, 1.04) | 0.345 | 1.10 (1.03, 1.18) | 0.005 | 0.92 (0.90, 0.94) | <0.001 | 1.01 (0.95, 1.06) | 0.843 | 1.10 (1.05, 1.16) | <0.001 |
| **Total cholesterol** | |  |  |  |  |  |  |  |  |  |  |  |
| Quintile 1 | Reference | | Reference | | Reference | | Reference | | Reference | | Reference | |
| Quintile 2 | 1.00 (0.93, 1.07) | 0.968 | 0.97 (0.81, 1.15) | 0.699 | 1.01 (0.85, 1.20) | 0.902 | 0.97 (0.89, 1.06) | 0.500 | 0.99 (0.89, 1.10) | 0.843 | 1.01 (0.89, 1.15) | 0.897 |
| Quintile 3 | 0.98 (0.91, 1.05) | 0.583 | 0.85 (0.68, 1.07) | 0.171 | 1.02 (0.83, 1.27) | 0.821 | 0.94 (0.86, 1.02) | 0.129 | 1.00 (0.87, 1.14) | 0.948 | 1.11 (0.96, 1.28) | 0.172 |
| Quintile 4 | 0.96 (0.89, 1.04) | 0.296 | 0.98 (0.76, 1.27) | 0.882 | 1.28 (1.03, 1.60) | 0.026 | 0.92 (0.85, 1.00) | 0.054 | 1.02 (0.85, 1.22) | 0.835 | 1.18 (0.99, 1.41) | 0.068 |
| Quintile 5 | 0.92 (0.86, 0.99) | 0.036 | 0.93 (0.70, 1.23) | 0.600 | 1.24 (0.97, 1.58) | 0.083 | 0.87 (0.80, 0.95) | 0.001 | 1.33 (1.10, 1.61) | 0.003 | 1.59 (1.32, 1.91) | <0.001 |
| Per 1-SD increase | 0.96 (0.94, 0.99) | 0.002 | 0.99 (0.91, 1.07) | 0.710 | 1.09 (1.03, 1.17) | 0.006 | 0.95 (0.92, 0.97) | <0.001 | 1.05 (1.00, 1.11) | 0.075 | 1.12 (1.07, 1.18) | <0.001 |
| **HDL cholesterol** | |  |  |  |  |  |  |  |  |  |  |  |
| Quintile 1 | Reference | | Reference | | Reference | | Reference | | Reference | | Reference | |
| Quintile 2 | 0.98 (0.91, 1.06) | 0.599 | 1.01 (0.84, 1.23) | 0.898 | 1.00 (0.83, 1.21) | 0.970 | 0.95 (0.87, 1.04) | 0.243 | 1.05 (0.93, 1.20) | 0.417 | 1.11 (0.96, 1.28) | 0.166 |
| Quintile 3 | 1.06 (0.99, 1.15) | 0.109 | 1.13 (0.92, 1.39) | 0.244 | 1.03 (0.85, 1.25) | 0.773 | 1.05 (0.96, 1.14) | 0.257 | 1.10 (0.96, 1.27) | 0.167 | 1.07 (0.92, 1.23) | 0.376 |
| Quintile 4 | 1.13 (1.05, 1.22) | 0.002 | 1.32 (1.06, 1.64) | 0.014 | 1.14 (0.94, 1.39) | 0.172 | 1.10 (1.01, 1.19) | 0.034 | 1.30 (1.12, 1.50) | <0.001 | 1.13 (0.98, 1.30) | 0.100 |
| Quintile 5 | 1.25 (1.15, 1.35) | <0.001 | 1.37 (1.08, 1.76) | 0.011 | 1.14 (0.94, 1.40) | 0.186 | 1.21 (1.11, 1.32) | <0.001 | 1.42 (1.21, 1.66) | <0.001 | 1.17 (1.02, 1.36) | 0.029 |
| Per 1-SD increase | 1.10 (1.07, 1.12) | <0.001 | 1.12 (1.03, 1.21) | 0.006 | 1.03 (0.97, 1.09) | 0.370 | 1.09 (1.06, 1.11) | <0.001 | 1.14 (1.08, 1.20) | <0.001 | 1.04 (1.00, 1.09) | 0.049 |
| **LDL cholesterol** | |  |  |  |  |  |  |  |  |  |  |  |
| Quintile 1 | Reference | | Reference | | Reference | | Reference | | Reference | | Reference | |
| Quintile 2 | 0.94 (0.88, 1.01) | 0.103 | 1.04 (0.88, 1.23) | 0.668 | 1.17 (0.99, 1.38) | 0.062 | 0.95 (0.87, 1.02) | 0.173 | 0.93 (0.83, 1.03) | 0.159 | 1.00 (0.89, 1.13) | 0.970 |
| Quintile 3 | 0.92 (0.86, 0.99) | 0.029 | 0.81 (0.64, 1.02) | 0.077 | 1.01 (0.82, 1.26) | 0.896 | 0.89 (0.82, 0.97) | 0.005 | 0.96 (0.83, 1.11) | 0.593 | 1.10 (0.95, 1.28) | 0.193 |
| Quintile 4 | 0.89 (0.82, 0.95) | <0.001 | 0.91 (0.70, 1.18) | 0.466 | 1.25 (0.99, 1.58) | 0.055 | 0.86 (0.79, 0.93) | <0.001 | 0.98 (0.81, 1.19) | 0.859 | 1.25 (1.04, 1.51) | 0.016 |
| Quintile 5 | 0.86 (0.80, 0.92) | <0.001 | 0.98 (0.74, 1.30) | 0.894 | 1.42 (1.12, 1.80) | 0.004 | 0.82 (0.76, 0.89) | <0.001 | 1.32 (1.09, 1.61) | 0.005 | 1.65 (1.36, 2.00) | <0.001 |
| Per 1-SD increase | 0.94 (0.92, 0.96) | <0.001 | 0.96 (0.89, 1.04) | 0.315 | 1.10 (1.03, 1.17) | 0.005 | 0.93 (0.90, 0.95) | <0.001 | 1.02 (0.97, 1.08) | 0.435 | 1.12 (1.06, 1.18) | <0.001 |
| **Triglycerides** | |  |  |  |  |  |  |  |  |  |  |  |
| Quintile 1 | Reference | | Reference | | Reference | | Reference | | Reference | | Reference | |
| Quintile 2 | 0.87 (0.82, 0.93) | <0.001 | 0.90 (0.73, 1.11) | 0.327 | 0.99 (0.81, 1.22) | 0.938 | 0.88 (0.82, 0.94) | <0.001 | 0.83 (0.72, 0.96) | 0.014 | 0.95 (0.82, 1.10) | 0.475 |
| Quintile 3 | 0.83 (0.78, 0.89) | <0.001 | 0.84 (0.67, 1.04) | 0.104 | 0.96 (0.79, 1.18) | 0.700 | 0.86 (0.80, 0.92) | <0.001 | 0.71 (0.61, 0.82) | <0.001 | 0.78 (0.67, 0.90) | <0.001 |
| Quintile 4 | 0.77 (0.72, 0.83) | <0.001 | 0.83 (0.67, 1.04) | 0.101 | 0.99 (0.81, 1.21) | 0.955 | 0.77 (0.72, 0.83) | <0.001 | 0.78 (0.67, 0.90) | <0.001 | 0.98 (0.85, 1.12) | 0.729 |
| Quintile 5 | 0.72 (0.67, 0.78) | <0.001 | 0.74 (0.59, 0.92) | 0.008 | 1.00 (0.82, 1.23) | 0.983 | 0.71 (0.65, 0.76) | <0.001 | 0.74 (0.64, 0.86) | <0.001 | 1.04 (0.90, 1.20) | 0.583 |
| Per 1-SD increase | 0.90 (0.88, 0.92) | <0.001 | 0.89 (0.83, 0.96) | 0.002 | 0.99 (0.92, 1.06) | 0.675 | 0.89 (0.86, 0.92) | <0.001 | 0.92 (0.88, 0.97) | <0.001 | 1.03 (0.99, 1.08) | 0.164 |
| **Lipoprotein A** | |  |  |  |  |  |  |  |  |  |  |  |
| Quintile 1 | Reference | | Reference | | Reference | | Reference | | Reference | | Reference | |
| Quintile 2 | 1.01 (0.95, 1.08) | 0.737 | 1.18 (0.95, 1.46) | 0.130 | 1.13 (0.92, 1.39) | 0.237 | 1.00 (0.93, 1.07) | 0.909 | 1.15 (1.00, 1.33) | 0.054 | 1.16 (1.00, 1.34) | 0.052 |
| Quintile 3 | 0.98 (0.92, 1.05) | 0.590 | 1.04 (0.84, 1.30) | 0.691 | 1.04 (0.85, 1.29) | 0.693 | 0.97 (0.90, 1.03) | 0.312 | 1.07 (0.93, 1.24) | 0.321 | 1.13 (0.98, 1.31) | 0.104 |
| Quintile 4 | 0.99 (0.93, 1.06) | 0.857 | 1.10 (0.89, 1.36) | 0.385 | 1.12 (0.91, 1.38) | 0.282 | 0.96 (0.90, 1.03) | 0.288 | 1.18 (1.03, 1.35) | 0.021 | 1.21 (1.04, 1.39) | 0.011 |
| Quintile 5 | 0.99 (0.92, 1.05) | 0.649 | 0.98 (0.80, 1.20) | 0.833 | 0.99 (0.81, 1.21) | 0.932 | 0.97 (0.91, 1.04) | 0.419 | 1.04 (0.91, 1.19) | 0.549 | 1.07 (0.93, 1.23) | 0.336 |
| Per 1-SD increase | 0.99 (0.97, 1.01) | 0.535 | 0.97 (0.92, 1.03) | 0.337 | 0.98 (0.93, 1.04) | 0.571 | 0.99 (0.97, 1.01) | 0.417 | 0.99 (0.96, 1.03) | 0.733 | 1.00 (0.96, 1.04) | 0.960 |

Notes: CVD = Cardiovascular disease; LLM = Lipid-lowering medications; HR = Hazard ratio; CI = Confidence interval; HDL = High density lipoprotein; LDL = Low density lipoprotein. Model was adjusted for age, sex (excluded in the model stratified by sex), ethnicity, deprivation index, current smokers, alcohol intake, sleep duration, total physical activity, total sedentary behavior, fruit and vegetables intake, red meat intake, processed meat intake, oily fish intake, ever eats eggs, ever eats dairy, body-mass index, waist-hip ratio, body fat percentage, number of morbidities, prefrail/frail status, CVD (excluded in the model stratified by CVD), hypertension, diabetes , chronic kidney disease, cancer, anemia, positive rheumatoid factor, vitamin D deficiency, osteoporosis, falls in the last year, lipid-lowering medications (excluded in the model stratified by use of LLM), aspirin, glucocorticoids, vitamin D supplements, and calcium supplements.

# Supplementary Table 17. Associations between serum lipid concentrations and the risk of hip fractures in the UK Biobank, stratified by age or sex.

| **Lipid traits** | **Age ≥ 60 years (ref)** | | **Age < 60 years** | | **Ratio of HR  (95% CI)** | **P-value** | **Women (ref)** | | **Men** | | **Ratio of HR  (95% CI)** | **P-value** |
| --- | --- | --- | --- | --- | --- | --- | --- | --- | --- | --- | --- | --- |
|  | **HR (95% CI)** | **P-value** | **HR (95% CI)** | **P-value** |  |  | **HR (95% CI)** | **P-value** | **HR (95% CI)** | **P-value** |  |  |
| **Apolipoprotein A** | |  |  |  |  |  |  |  |  |  |  |  |
| Quintile 1 | Reference | | Reference | | Reference | | Reference | | Reference | | Reference | |
| Quintile 2 | 0.90 (0.78, 1.03) | 0.135 | 1.07 (0.82, 1.39) | 0.621 | 1.06 (0.81, 1.39) | 0.679 | 0.96 (0.79, 1.16) | 0.643 | 0.87 (0.74, 1.03) | 0.110 | 0.92 (0.72, 1.16) | 0.483 |
| Quintile 3 | 0.95 (0.82, 1.09) | 0.445 | 1.49 (1.16, 1.92) | 0.002 | 1.13 (0.87, 1.46) | 0.367 | 1.03 (0.86, 1.24) | 0.741 | 1.00 (0.84, 1.19) | 0.991 | 0.93 (0.74, 1.18) | 0.566 |
| Quintile 4 | 0.86 (0.75, 1.00) | 0.046 | 1.51 (1.17, 1.95) | 0.002 | 1.30 (1.01, 1.68) | 0.043 | 0.94 (0.78, 1.12) | 0.464 | 0.92 (0.75, 1.12) | 0.391 | 1.02 (0.80, 1.29) | 0.878 |
| Quintile 5 | 0.90 (0.77, 1.04) | 0.144 | 1.65 (1.26, 2.15) | <0.001 | 1.21 (0.94, 1.56) | 0.130 | 0.93 (0.78, 1.11) | 0.427 | 1.09 (0.88, 1.36) | 0.431 | 1.16 (0.91, 1.49) | 0.238 |
| Per 1-SD increase | 0.99 (0.94, 1.03) | 0.558 | 1.19 (1.10, 1.28) | <0.001 | 1.07 (1.00, 1.15) | 0.062 | 0.99 (0.94, 1.04) | 0.603 | 1.04 (0.96, 1.12) | 0.333 | 1.06 (0.99, 1.15) | 0.095 |
| **Apolipoprotein B** | |  |  |  |  |  |  |  |  |  |  |  |
| Quintile 1 | Reference | | Reference | | Reference | | Reference | | Reference | | Reference | |
| Quintile 2 | 0.91 (0.80, 1.03) | 0.122 | 1.00 (0.81, 1.24) | 0.977 | 1.00 (0.80, 1.25) | 0.984 | 0.90 (0.78, 1.03) | 0.137 | 0.94 (0.80, 1.12) | 0.502 | 1.07 (0.87, 1.30) | 0.517 |
| Quintile 3 | 0.82 (0.71, 0.93) | 0.003 | 0.98 (0.78, 1.22) | 0.827 | 1.08 (0.86, 1.37) | 0.499 | 0.87 (0.75, 1.01) | 0.060 | 0.74 (0.61, 0.90) | 0.002 | 0.91 (0.74, 1.12) | 0.383 |
| Quintile 4 | 0.79 (0.69, 0.91) | <0.001 | 0.95 (0.75, 1.19) | 0.641 | 1.02 (0.80, 1.30) | 0.861 | 0.83 (0.71, 0.96) | 0.011 | 0.72 (0.59, 0.88) | 0.001 | 0.93 (0.75, 1.15) | 0.493 |
| Quintile 5 | 0.79 (0.68, 0.90) | <0.001 | 0.87 (0.68, 1.12) | 0.294 | 0.92 (0.71, 1.18) | 0.494 | 0.77 (0.66, 0.90) | <0.001 | 0.76 (0.62, 0.94) | 0.010 | 1.04 (0.83, 1.29) | 0.746 |
| Per 1-SD increase | 0.93 (0.89, 0.97) | 0.001 | 0.93 (0.86, 1.01) | 0.096 | 0.94 (0.87, 1.02) | 0.147 | 0.91 (0.87, 0.96) | <0.001 | 0.90 (0.84, 0.96) | 0.003 | 1.00 (0.93, 1.07) | 0.962 |
| **Total cholesterol** | |  |  |  |  |  |  |  |  |  |  |  |
| Quintile 1 | Reference | | Reference | | Reference | | Reference | | Reference | | Reference | |
| Quintile 2 | 0.89 (0.78, 1.01) | 0.079 | 0.96 (0.77, 1.21) | 0.741 | 0.85 (0.67, 1.07) | 0.169 | 0.95 (0.81, 1.11) | 0.538 | 0.81 (0.68, 0.97) | 0.018 | 0.95 (0.77, 1.16) | 0.601 |
| Quintile 3 | 0.86 (0.75, 1.00) | 0.044 | 1.08 (0.86, 1.36) | 0.500 | 1.00 (0.79, 1.26) | 0.974 | 0.96 (0.81, 1.12) | 0.588 | 0.77 (0.63, 0.93) | 0.006 | 0.94 (0.76, 1.16) | 0.571 |
| Quintile 4 | 0.78 (0.68, 0.91) | 0.001 | 1.03 (0.81, 1.31) | 0.806 | 0.98 (0.77, 1.25) | 0.876 | 0.86 (0.73, 1.01) | 0.072 | 0.70 (0.57, 0.86) | <0.001 | 0.90 (0.72, 1.13) | 0.366 |
| Quintile 5 | 0.75 (0.64, 0.87) | <0.001 | 1.05 (0.82, 1.35) | 0.684 | 0.93 (0.73, 1.20) | 0.595 | 0.80 (0.67, 0.94) | 0.007 | 0.73 (0.58, 0.91) | 0.005 | 1.05 (0.83, 1.31) | 0.701 |
| Per 1-SD increase | 0.91 (0.87, 0.95) | <0.001 | 1.00 (0.92, 1.09) | 0.949 | 0.97 (0.89, 1.05) | 0.447 | 0.91 (0.87, 0.96) | <0.001 | 0.89 (0.83, 0.96) | 0.001 | 1.02 (0.95, 1.09) | 0.586 |
| **HDL cholesterol** | |  |  |  |  |  |  |  |  |  |  |  |
| Quintile 1 | Reference | | Reference | | Reference | | Reference | | Reference | | Reference | |
| Quintile 2 | 0.93 (0.81, 1.07) | 0.331 | 0.94 (0.72, 1.23) | 0.657 | 0.82 (0.62, 1.09) | 0.168 | 0.85 (0.70, 1.03) | 0.095 | 0.92 (0.78, 1.09) | 0.349 | 1.08 (0.86, 1.37) | 0.514 |
| Quintile 3 | 0.97 (0.84, 1.12) | 0.650 | 1.32 (1.02, 1.72) | 0.034 | 1.00 (0.77, 1.30) | 0.989 | 0.91 (0.76, 1.09) | 0.301 | 1.04 (0.87, 1.24) | 0.694 | 1.17 (0.93, 1.47) | 0.180 |
| Quintile 4 | 0.98 (0.85, 1.14) | 0.824 | 1.47 (1.13, 1.92) | 0.005 | 0.98 (0.76, 1.26) | 0.851 | 0.95 (0.79, 1.13) | 0.543 | 1.02 (0.83, 1.24) | 0.872 | 1.14 (0.90, 1.44) | 0.289 |
| Quintile 5 | 1.04 (0.89, 1.21) | 0.597 | 1.73 (1.31, 2.29) | <0.001 | 1.04 (0.81, 1.32) | 0.774 | 0.96 (0.80, 1.16) | 0.689 | 1.36 (1.09, 1.70) | 0.006 | 1.60 (1.26, 2.03) | <0.001 |
| Per 1-SD increase | 1.03 (0.98, 1.08) | 0.203 | 1.20 (1.11, 1.30) | <0.001 | 1.03 (0.96, 1.11) | 0.437 | 1.03 (0.98, 1.08) | 0.250 | 1.08 (1.00, 1.16) | 0.053 | 1.11 (1.03, 1.19) | 0.005 |
| **LDL cholesterol** | |  |  |  |  |  |  |  |  |  |  |  |
| Quintile 1 | Reference | | Reference | | Reference | | Reference | | Reference | | Reference | |
| Quintile 2 | 0.90 (0.79, 1.03) | 0.117 | 1.01 (0.81, 1.25) | 0.953 | 0.92 (0.73, 1.15) | 0.456 | 0.95 (0.82, 1.10) | 0.490 | 0.85 (0.72, 1.01) | 0.069 | 0.97 (0.79, 1.18) | 0.763 |
| Quintile 3 | 0.81 (0.71, 0.93) | 0.004 | 0.94 (0.74, 1.18) | 0.569 | 0.95 (0.75, 1.20) | 0.665 | 0.89 (0.76, 1.04) | 0.133 | 0.69 (0.57, 0.84) | <0.001 | 0.92 (0.74, 1.14) | 0.436 |
| Quintile 4 | 0.77 (0.67, 0.89) | <0.001 | 1.07 (0.85, 1.36) | 0.548 | 1.09 (0.86, 1.38) | 0.472 | 0.86 (0.73, 1.01) | 0.061 | 0.70 (0.57, 0.86) | <0.001 | 0.91 (0.73, 1.13) | 0.389 |
| Quintile 5 | 0.76 (0.66, 0.88) | <0.001 | 0.90 (0.70, 1.16) | 0.402 | 0.84 (0.66, 1.09) | 0.195 | 0.78 (0.66, 0.91) | 0.002 | 0.70 (0.57, 0.87) | 0.002 | 1.03 (0.83, 1.29) | 0.790 |
| Per 1-SD increase | 0.91 (0.86, 0.95) | <0.001 | 0.95 (0.88, 1.03) | 0.217 | 0.94 (0.87, 1.02) | 0.134 | 0.91 (0.86, 0.95) | <0.001 | 0.87 (0.81, 0.94) | <0.001 | 1.00 (0.93, 1.07) | 0.987 |
| **Triglycerides** | |  |  |  |  |  |  |  |  |  |  |  |
| Quintile 1 | Reference | | Reference | | Reference | | Reference | | Reference | | Reference | |
| Quintile 2 | 0.88 (0.78, 1.00) | 0.048 | 0.89 (0.72, 1.10) | 0.278 | 1.07 (0.86, 1.34) | 0.550 | 0.87 (0.77, 1.00) | 0.044 | 0.82 (0.68, 0.99) | 0.044 | 0.81 (0.66, 1.01) | 0.058 |
| Quintile 3 | 0.76 (0.67, 0.87) | <0.001 | 0.89 (0.71, 1.11) | 0.303 | 1.28 (1.01, 1.61) | 0.039 | 0.77 (0.67, 0.88) | <0.001 | 0.73 (0.60, 0.88) | 0.001 | 0.80 (0.64, 0.99) | 0.039 |
| Quintile 4 | 0.76 (0.66, 0.87) | <0.001 | 0.90 (0.71, 1.14) | 0.389 | 1.31 (1.04, 1.65) | 0.024 | 0.76 (0.66, 0.88) | <0.001 | 0.75 (0.61, 0.90) | 0.003 | 0.75 (0.61, 0.93) | 0.009 |
| Quintile 5 | 0.70 (0.61, 0.81) | <0.001 | 0.72 (0.55, 0.93) | 0.011 | 1.15 (0.90, 1.47) | 0.268 | 0.69 (0.59, 0.81) | <0.001 | 0.69 (0.57, 0.84) | <0.001 | 0.80 (0.64, 1.00) | 0.045 |
| Per 1-SD increase | 0.88 (0.84, 0.93) | <0.001 | 0.92 (0.85, 1.01) | 0.066 | 1.09 (1.01, 1.18) | 0.033 | 0.87 (0.82, 0.92) | <0.001 | 0.91 (0.85, 0.97) | 0.004 | 0.96 (0.89, 1.04) | 0.299 |
| **Lipoprotein A** | |  |  |  |  |  |  |  |  |  |  |  |
| Quintile 1 | Reference | | Reference | | Reference | | Reference | | Reference | | Reference | |
| Quintile 2 | 0.97 (0.85, 1.10) | 0.650 | 0.99 (0.79, 1.23) | 0.904 | 0.99 (0.78, 1.25) | 0.922 | 0.90 (0.78, 1.04) | 0.152 | 1.07 (0.90, 1.28) | 0.428 | 1.18 (0.95, 1.46) | 0.128 |
| Quintile 3 | 0.92 (0.80, 1.04) | 0.179 | 1.15 (0.93, 1.43) | 0.198 | 1.15 (0.91, 1.45) | 0.255 | 0.90 (0.78, 1.04) | 0.141 | 1.03 (0.86, 1.24) | 0.728 | 1.17 (0.94, 1.45) | 0.157 |
| Quintile 4 | 0.99 (0.87, 1.12) | 0.824 | 0.97 (0.77, 1.22) | 0.785 | 0.90 (0.70, 1.15) | 0.387 | 0.95 (0.83, 1.10) | 0.513 | 0.96 (0.80, 1.16) | 0.677 | 1.01 (0.81, 1.25) | 0.952 |
| Quintile 5 | 0.99 (0.87, 1.12) | 0.833 | 0.92 (0.73, 1.16) | 0.482 | 0.85 (0.67, 1.09) | 0.199 | 0.97 (0.84, 1.11) | 0.611 | 0.91 (0.76, 1.10) | 0.340 | 0.93 (0.75, 1.16) | 0.516 |
| Per 1-SD increase | 1.01 (0.97, 1.05) | 0.748 | 0.96 (0.90, 1.04) | 0.338 | 0.95 (0.88, 1.02) | 0.170 | 1.01 (0.97, 1.05) | 0.696 | 0.96 (0.90, 1.02) | 0.179 | 0.95 (0.88, 1.01) | 0.115 |

Notes: HR = Hazard ratio; CI = Confidence interval; HDL = High density lipoprotein; LDL = Low density lipoprotein. Model was adjusted for age (excluded in the model stratified by age), sex (excluded in the model stratified by sex), ethnicity, deprivation index, current smokers, alcohol intake, sleep duration, total physical activity, total sedentary behavior, fruit and vegetables intake, red meat intake, processed meat intake, oily fish intake, ever eats eggs, ever eats dairy, body-mass index, waist-hip ratio, body fat percentage, number of morbidities, prefrail/frail status, cardiovascular disease, hypertension, diabetes , chronic kidney disease, cancer, anemia, positive rheumatoid factor, vitamin D deficiency, osteoporosis, falls in the last year, menopause status (included in the model for women), lipid-lowering medications, aspirin, glucocorticoids, vitamin D supplements, and calcium supplements.

# Supplementary Table 18. Associations between serum lipid concentrations and the risk of hip fractures in the UK Biobank, stratified by BMI.

| **Lipid traits** | **Normal weight (ref)** | | **Overweight** | | **Ratio of HR  (95% CI)** | **P-value** | **Obese** | | **Ratio of HR  (95% CI)** | **P-value** |
| --- | --- | --- | --- | --- | --- | --- | --- | --- | --- | --- |
|  | **HR (95% CI)** | **P-value** | **HR (95% CI)** | **P-value** |  |  | **HR (95% CI)** | **P-value** |  |  |
| **Apolipoprotein A** |  |  |  |  |  |  |  |  |  |  |
| Quintile 1 | Reference | | Reference | | Reference | | Reference | | Reference | |
| Quintile 2 | 0.94 (0.75, 1.19) | 0.624 | 0.93 (0.76, 1.13) | 0.446 | 1.03 (0.77, 1.36) | 0.862 | 0.85 (0.67, 1.07) | 0.164 | 0.98 (0.72, 1.33) | 0.898 |
| Quintile 3 | 1.04 (0.84, 1.30) | 0.700 | 1.09 (0.90, 1.31) | 0.397 | 1.16 (0.89, 1.52) | 0.262 | 0.83 (0.65, 1.06) | 0.136 | 1.06 (0.79, 1.42) | 0.702 |
| Quintile 4 | 0.92 (0.74, 1.14) | 0.441 | 1.00 (0.82, 1.23) | 0.964 | 1.25 (0.96, 1.61) | 0.097 | 0.75 (0.57, 0.99) | 0.040 | 0.96 (0.71, 1.30) | 0.776 |
| Quintile 5 | 0.95 (0.77, 1.18) | 0.665 | 1.01 (0.82, 1.24) | 0.947 | 1.20 (0.93, 1.54) | 0.157 | 0.84 (0.62, 1.14) | 0.259 | 1.00 (0.73, 1.37) | 0.989 |
| Per 1-SD increase | 1.01 (0.95, 1.07) | 0.784 | 1.01 (0.95, 1.08) | 0.674 | 1.05 (0.98, 1.13) | 0.157 | 0.91 (0.82, 1.01) | 0.080 | 0.97 (0.88, 1.07) | 0.505 |
| **Apolipoprotein B** |  |  |  |  |  |  |  |  |  |  |
| Quintile 1 | Reference | | Reference | | Reference | | Reference | | Reference | |
| Quintile 2 | 0.84 (0.71, 0.98) | 0.030 | 0.93 (0.77, 1.12) | 0.463 | 1.18 (0.94, 1.47) | 0.156 | 1.10 (0.86, 1.39) | 0.458 | 1.29 (0.99, 1.68) | 0.055 |
| Quintile 3 | 0.72 (0.60, 0.86) | <0.001 | 0.94 (0.78, 1.14) | 0.532 | 1.34 (1.07, 1.69) | 0.011 | 0.97 (0.74, 1.27) | 0.829 | 1.32 (1.00, 1.75) | 0.049 |
| Quintile 4 | 0.77 (0.65, 0.92) | 0.004 | 0.84 (0.69, 1.03) | 0.091 | 1.20 (0.95, 1.51) | 0.120 | 0.87 (0.65, 1.15) | 0.326 | 1.18 (0.89, 1.56) | 0.256 |
| Quintile 5 | 0.71 (0.58, 0.85) | <0.001 | 0.85 (0.69, 1.03) | 0.102 | 1.28 (1.02, 1.61) | 0.037 | 0.89 (0.67, 1.19) | 0.433 | 1.28 (0.97, 1.69) | 0.085 |
| Per 1-SD increase | 0.89 (0.83, 0.95) | <0.001 | 0.94 (0.88, 1.00) | 0.068 | 1.09 (1.02, 1.18) | 0.017 | 0.95 (0.86, 1.04) | 0.236 | 1.08 (0.99, 1.18) | 0.091 |
| **Total cholesterol** |  |  |  |  |  |  |  |  |  |  |
| Quintile 1 | Reference | | Reference | | Reference | | Reference | | Reference | |
| Quintile 2 | 0.85 (0.71, 1.02) | 0.079 | 0.88 (0.72, 1.07) | 0.186 | 1.00 (0.79, 1.27) | 0.999 | 0.92 (0.72, 1.17) | 0.480 | 1.09 (0.84, 1.43) | 0.516 |
| Quintile 3 | 0.87 (0.72, 1.05) | 0.143 | 0.89 (0.72, 1.08) | 0.241 | 1.06 (0.84, 1.34) | 0.598 | 0.84 (0.64, 1.11) | 0.220 | 0.98 (0.74, 1.30) | 0.906 |
| Quintile 4 | 0.71 (0.58, 0.86) | <0.001 | 0.92 (0.75, 1.13) | 0.432 | 1.35 (1.07, 1.70) | 0.011 | 0.76 (0.57, 1.02) | 0.072 | 1.17 (0.88, 1.55) | 0.272 |
| Quintile 5 | 0.77 (0.63, 0.93) | 0.008 | 0.76 (0.61, 0.94) | 0.011 | 1.11 (0.88, 1.39) | 0.373 | 0.77 (0.57, 1.03) | 0.076 | 1.05 (0.79, 1.38) | 0.753 |
| Per 1-SD increase | 0.90 (0.84, 0.96) | 0.002 | 0.92 (0.86, 0.99) | 0.023 | 1.08 (1.00, 1.16) | 0.039 | 0.91 (0.82, 1.00) | 0.048 | 1.05 (0.96, 1.14) | 0.315 |
| **HDL cholesterol** |  |  |  |  |  |  |  |  |  |  |
| Quintile 1 | Reference | | Reference | | Reference | | Reference | | Reference | |
| Quintile 2 | 0.88 (0.69, 1.14) | 0.341 | 0.94 (0.77, 1.14) | 0.511 | 1.12 (0.83, 1.50) | 0.459 | 0.87 (0.70, 1.09) | 0.231 | 1.11 (0.82, 1.52) | 0.491 |
| Quintile 3 | 1.03 (0.81, 1.30) | 0.821 | 1.06 (0.87, 1.28) | 0.580 | 1.17 (0.89, 1.54) | 0.256 | 0.78 (0.60, 1.00) | 0.055 | 0.98 (0.73, 1.33) | 0.922 |
| Quintile 4 | 1.01 (0.80, 1.28) | 0.927 | 1.05 (0.86, 1.29) | 0.621 | 1.25 (0.96, 1.63) | 0.103 | 0.99 (0.75, 1.29) | 0.928 | 1.10 (0.81, 1.50) | 0.541 |
| Quintile 5 | 1.10 (0.87, 1.39) | 0.445 | 1.10 (0.89, 1.36) | 0.391 | 1.17 (0.90, 1.52) | 0.237 | 0.90 (0.64, 1.27) | 0.559 | 0.97 (0.69, 1.37) | 0.874 |
| Per 1-SD increase | 1.04 (0.98, 1.10) | 0.212 | 1.05 (0.98, 1.13) | 0.154 | 1.05 (0.98, 1.13) | 0.151 | 0.97 (0.87, 1.09) | 0.629 | 0.98 (0.88, 1.08) | 0.628 |
| **LDL cholesterol** |  |  |  |  |  |  |  |  |  |  |
| Quintile 1 | Reference | | Reference | | Reference | | Reference | | Reference | |
| Quintile 2 | 0.84 (0.71, 1.00) | 0.045 | 1.00 (0.83, 1.21) | 0.993 | 1.22 (0.97, 1.53) | 0.083 | 0.98 (0.77, 1.24) | 0.843 | 1.15 (0.88, 1.50) | 0.297 |
| Quintile 3 | 0.77 (0.64, 0.92) | 0.004 | 0.83 (0.68, 1.02) | 0.079 | 1.16 (0.92, 1.46) | 0.213 | 0.96 (0.73, 1.27) | 0.782 | 1.21 (0.92, 1.59) | 0.177 |
| Quintile 4 | 0.75 (0.62, 0.90) | 0.002 | 0.91 (0.74, 1.12) | 0.392 | 1.27 (1.01, 1.60) | 0.038 | 0.79 (0.58, 1.06) | 0.113 | 1.08 (0.82, 1.44) | 0.578 |
| Quintile 5 | 0.73 (0.60, 0.88) | 0.001 | 0.79 (0.64, 0.98) | 0.031 | 1.24 (0.99, 1.56) | 0.062 | 0.83 (0.62, 1.12) | 0.227 | 1.19 (0.90, 1.57) | 0.224 |
| Per 1-SD increase | 0.89 (0.83, 0.95) | <0.001 | 0.92 (0.86, 0.98) | 0.009 | 1.07 (1.00, 1.15) | 0.054 | 0.91 (0.83, 1.01) | 0.069 | 1.05 (0.96, 1.15) | 0.252 |
| **Triglycerides** |  |  |  |  |  |  |  |  |  |  |
| Quintile 1 | Reference | | Reference | | Reference | | Reference | | Reference | |
| Quintile 2 | 0.87 (0.75, 1.00) | 0.049 | 0.83 (0.69, 1.01) | 0.061 | 1.03 (0.83, 1.29) | 0.777 | 1.05 (0.72, 1.55) | 0.792 | 1.33 (0.91, 1.94) | 0.141 |
| Quintile 3 | 0.76 (0.64, 0.89) | <0.001 | 0.74 (0.61, 0.89) | 0.002 | 1.03 (0.82, 1.29) | 0.807 | 0.97 (0.67, 1.41) | 0.870 | 1.34 (0.92, 1.94) | 0.124 |
| Quintile 4 | 0.74 (0.61, 0.88) | <0.001 | 0.73 (0.60, 0.88) | <0.001 | 1.01 (0.80, 1.28) | 0.947 | 1.04 (0.72, 1.48) | 0.848 | 1.56 (1.09, 2.24) | 0.016 |
| Quintile 5 | 0.71 (0.57, 0.88) | 0.002 | 0.68 (0.56, 0.83) | <0.001 | 0.97 (0.74, 1.25) | 0.790 | 0.87 (0.60, 1.24) | 0.433 | 1.38 (0.95, 2.01) | 0.095 |
| Per 1-SD increase | 0.84 (0.77, 0.91) | <0.001 | 0.91 (0.85, 0.97) | 0.004 | 1.07 (0.97, 1.17) | 0.187 | 0.93 (0.85, 1.01) | 0.066 | 1.12 (1.01, 1.25) | 0.027 |
| **Lipoprotein A** |  |  |  |  |  |  |  |  |  |  |
| Quintile 1 | Reference | | Reference | | Reference | | Reference | | Reference | |
| Quintile 2 | 0.99 (0.83, 1.17) | 0.877 | 0.92 (0.76, 1.10) | 0.358 | 1.00 (0.79, 1.27) | 0.995 | 1.00 (0.78, 1.29) | 0.996 | 0.99 (0.74, 1.31) | 0.920 |
| Quintile 3 | 0.94 (0.80, 1.12) | 0.517 | 0.95 (0.80, 1.14) | 0.602 | 1.09 (0.87, 1.38) | 0.453 | 0.97 (0.75, 1.26) | 0.834 | 1.03 (0.77, 1.37) | 0.841 |
| Quintile 4 | 0.95 (0.80, 1.14) | 0.602 | 0.99 (0.83, 1.18) | 0.915 | 1.12 (0.89, 1.41) | 0.350 | 0.97 (0.75, 1.25) | 0.796 | 0.98 (0.74, 1.31) | 0.914 |
| Quintile 5 | 0.98 (0.83, 1.16) | 0.824 | 0.85 (0.71, 1.02) | 0.081 | 0.97 (0.77, 1.22) | 0.775 | 1.17 (0.92, 1.49) | 0.209 | 1.21 (0.92, 1.59) | 0.166 |
| Per 1-SD increase | 1.00 (0.94, 1.05) | 0.899 | 0.97 (0.92, 1.03) | 0.272 | 0.99 (0.93, 1.07) | 0.880 | 1.05 (0.97, 1.13) | 0.202 | 1.07 (0.98, 1.16) | 0.141 |

Notes: HR = Hazard ratio; CI = Confidence interval; HDL = High density lipoprotein; LDL = Low density lipoprotein. Model was adjusted for age, sex, ethnicity, deprivation index, current smokers, alcohol intake, sleep duration, total physical activity, total sedentary behavior, fruit and vegetables intake, red meat intake, processed meat intake, oily fish intake, ever eats eggs, ever eats dairy, waist-hip ratio, body fat percentage, number of morbidities, prefrail/frail status, cardiovascular disease, hypertension, diabetes , chronic kidney disease, cancer, anemia, positive rheumatoid factor, vitamin D deficiency, osteoporosis, falls in the last year, lipid-lowering medications, aspirin, glucocorticoids, vitamin D supplements, and calcium supplements.

# Supplementary Table 19. Associations between serum lipid concentrations and the risk of hip fractures in the UK Biobank, stratified by CVD or use of lipid-lowering medications.

| **Lipid traits** | **Non-CVD (ref)** | | **CVD** | | **Ratio of HR  (95% CI)** | **P-value** | **Not taking LLM (ref)** | | **Taking LLM** | | **Ratio of HR  (95% CI)** | **P-value** |
| --- | --- | --- | --- | --- | --- | --- | --- | --- | --- | --- | --- | --- |
|  | **HR (95% CI)** | **P-value** | **HR (95% CI)** | **P-value** |  |  | **HR (95% CI)** | **P-value** | **HR (95% CI)** | **P-value** |  |  |
| **Apolipoprotein A** | |  |  |  |  |  |  |  |  |  |  |  |
| Quintile 1 | Reference | | Reference | | Reference | | Reference | | Reference | | Reference | |
| Quintile 2 | 0.92 (0.80, 1.05) | 0.219 | 0.89 (0.65, 1.21) | 0.443 | 0.95 (0.70, 1.30) | 0.766 | 0.92 (0.79, 1.07) | 0.268 | 0.90 (0.72, 1.12) | 0.351 | 1.02 (0.80, 1.31) | 0.865 |
| Quintile 3 | 1.01 (0.88, 1.15) | 0.902 | 1.12 (0.82, 1.53) | 0.470 | 1.06 (0.78, 1.43) | 0.719 | 1.00 (0.87, 1.16) | 0.981 | 1.08 (0.87, 1.35) | 0.482 | 1.12 (0.88, 1.42) | 0.347 |
| Quintile 4 | 0.93 (0.81, 1.06) | 0.270 | 1.01 (0.71, 1.42) | 0.972 | 1.15 (0.85, 1.58) | 0.365 | 0.93 (0.80, 1.08) | 0.339 | 0.95 (0.75, 1.21) | 0.681 | 1.11 (0.87, 1.41) | 0.412 |
| Quintile 5 | 0.95 (0.82, 1.09) | 0.449 | 1.04 (0.72, 1.50) | 0.850 | 1.09 (0.80, 1.50) | 0.577 | 0.95 (0.82, 1.11) | 0.531 | 0.98 (0.76, 1.26) | 0.869 | 1.07 (0.84, 1.36) | 0.581 |
| Per 1-SD increase | 1.00 (0.96, 1.04) | 0.930 | 1.02 (0.91, 1.15) | 0.697 | 1.03 (0.94, 1.14) | 0.506 | 0.99 (0.95, 1.04) | 0.819 | 1.04 (0.96, 1.12) | 0.361 | 1.05 (0.97, 1.12) | 0.224 |
| **Apolipoprotein B** | |  |  |  |  |  |  |  |  |  |  |  |
| Quintile 1 | Reference | | Reference | | Reference | | Reference | | Reference | | Reference | |
| Quintile 2 | 0.85 (0.76, 0.96) | 0.009 | 1.23 (0.96, 1.58) | 0.102 | 1.46 (1.13, 1.88) | 0.004 | 0.88 (0.77, 1.02) | 0.084 | 0.92 (0.77, 1.10) | 0.353 | 1.05 (0.85, 1.29) | 0.653 |
| Quintile 3 | 0.80 (0.71, 0.90) | <0.001 | 0.80 (0.57, 1.14) | 0.226 | 1.11 (0.80, 1.55) | 0.532 | 0.76 (0.66, 0.88) | <0.001 | 0.96 (0.78, 1.19) | 0.725 | 1.18 (0.93, 1.50) | 0.162 |
| Quintile 4 | 0.75 (0.66, 0.85) | <0.001 | 1.03 (0.70, 1.51) | 0.873 | 1.48 (1.04, 2.10) | 0.028 | 0.74 (0.64, 0.84) | <0.001 | 0.89 (0.67, 1.18) | 0.406 | 1.31 (0.99, 1.73) | 0.056 |
| Quintile 5 | 0.73 (0.64, 0.83) | <0.001 | 0.96 (0.62, 1.50) | 0.871 | 1.42 (0.97, 2.09) | 0.075 | 0.71 (0.62, 0.82) | <0.001 | 0.97 (0.69, 1.35) | 0.855 | 1.51 (1.10, 2.07) | 0.010 |
| Per 1-SD increase | 0.90 (0.86, 0.94) | <0.001 | 0.98 (0.86, 1.11) | 0.701 | 1.12 (1.00, 1.24) | 0.043 | 0.89 (0.85, 0.93) | <0.001 | 0.97 (0.89, 1.06) | 0.501 | 1.11 (1.02, 1.22) | 0.015 |
| **Total cholesterol** | |  |  |  |  |  |  |  |  |  |  |  |
| Quintile 1 | Reference | | Reference | | Reference | | Reference | | Reference | | Reference | |
| Quintile 2 | 0.90 (0.79, 1.02) | 0.102 | 0.81 (0.61, 1.08) | 0.156 | 0.92 (0.70, 1.22) | 0.576 | 0.87 (0.75, 1.02) | 0.078 | 0.83 (0.69, 1.00) | 0.053 | 0.93 (0.75, 1.15) | 0.504 |
| Quintile 3 | 0.89 (0.78, 1.01) | 0.074 | 0.65 (0.43, 0.97) | 0.034 | 0.87 (0.61, 1.25) | 0.456 | 0.84 (0.72, 0.97) | 0.019 | 0.84 (0.66, 1.07) | 0.160 | 1.06 (0.82, 1.36) | 0.659 |
| Quintile 4 | 0.78 (0.68, 0.89) | <0.001 | 1.02 (0.68, 1.52) | 0.930 | 1.61 (1.14, 2.25) | 0.006 | 0.73 (0.63, 0.85) | <0.001 | 0.96 (0.71, 1.31) | 0.814 | 1.37 (1.01, 1.84) | 0.041 |
| Quintile 5 | 0.74 (0.65, 0.85) | <0.001 | 0.88 (0.56, 1.41) | 0.606 | 1.27 (0.86, 1.89) | 0.236 | 0.68 (0.59, 0.80) | <0.001 | 1.36 (1.00, 1.86) | 0.053 | 2.04 (1.51, 2.77) | <0.001 |
| Per 1-SD increase | 0.89 (0.85, 0.93) | <0.001 | 0.99 (0.87, 1.12) | 0.882 | 1.13 (1.02, 1.26) | 0.016 | 0.87 (0.83, 0.92) | <0.001 | 1.01 (0.92, 1.10) | 0.890 | 1.18 (1.08, 1.28) | <0.001 |
| **HDL cholesterol** | |  |  |  |  |  |  |  |  |  |  |  |
| Quintile 1 | Reference | | Reference | | Reference | | Reference | | Reference | | Reference | |
| Quintile 2 | 0.92 (0.80, 1.06) | 0.243 | 0.84 (0.61, 1.15) | 0.270 | 0.90 (0.66, 1.23) | 0.498 | 0.87 (0.74, 1.01) | 0.071 | 0.97 (0.79, 1.20) | 0.781 | 1.11 (0.87, 1.41) | 0.401 |
| Quintile 3 | 0.98 (0.85, 1.12) | 0.757 | 1.12 (0.81, 1.55) | 0.485 | 1.10 (0.81, 1.48) | 0.558 | 0.96 (0.82, 1.11) | 0.555 | 1.06 (0.85, 1.33) | 0.617 | 1.13 (0.89, 1.43) | 0.302 |
| Quintile 4 | 1.00 (0.87, 1.15) | 0.968 | 1.27 (0.90, 1.79) | 0.172 | 1.31 (0.97, 1.77) | 0.082 | 0.96 (0.82, 1.12) | 0.599 | 1.19 (0.94, 1.51) | 0.160 | 1.21 (0.95, 1.54) | 0.119 |
| Quintile 5 | 1.06 (0.92, 1.23) | 0.400 | 1.34 (0.92, 1.97) | 0.132 | 1.30 (0.95, 1.77) | 0.101 | 1.02 (0.87, 1.19) | 0.840 | 1.31 (1.02, 1.70) | 0.038 | 1.36 (1.07, 1.72) | 0.012 |
| Per 1-SD increase | 1.03 (0.99, 1.08) | 0.126 | 1.13 (1.00, 1.27) | 0.057 | 1.09 (0.99, 1.19) | 0.083 | 1.02 (0.97, 1.07) | 0.417 | 1.13 (1.04, 1.23) | 0.004 | 1.11 (1.03, 1.19) | 0.005 |
| **LDL cholesterol** | |  |  |  |  |  |  |  |  |  |  |  |
| Quintile 1 | Reference | | Reference | | Reference | | Reference | | Reference | | Reference | |
| Quintile 2 | 0.90 (0.80, 1.02) | 0.094 | 0.90 (0.68, 1.18) | 0.445 | 1.05 (0.80, 1.37) | 0.714 | 0.90 (0.78, 1.05) | 0.171 | 0.85 (0.71, 1.01) | 0.070 | 0.93 (0.75, 1.15) | 0.483 |
| Quintile 3 | 0.79 (0.70, 0.90) | <0.001 | 0.83 (0.57, 1.21) | 0.340 | 1.19 (0.85, 1.66) | 0.314 | 0.77 (0.67, 0.89) | <0.001 | 0.82 (0.64, 1.06) | 0.126 | 1.07 (0.82, 1.39) | 0.613 |
| Quintile 4 | 0.78 (0.68, 0.89) | <0.001 | 0.79 (0.50, 1.24) | 0.299 | 1.10 (0.73, 1.64) | 0.656 | 0.74 (0.64, 0.86) | <0.001 | 1.02 (0.74, 1.39) | 0.923 | 1.47 (1.08, 1.99) | 0.013 |
| Quintile 5 | 0.71 (0.62, 0.82) | <0.001 | 1.15 (0.74, 1.77) | 0.532 | 1.63 (1.12, 2.37) | 0.010 | 0.68 (0.59, 0.79) | <0.001 | 1.28 (0.91, 1.80) | 0.155 | 1.87 (1.34, 2.61) | <0.001 |
| Per 1-SD increase | 0.88 (0.85, 0.92) | <0.001 | 0.97 (0.85, 1.10) | 0.615 | 1.12 (1.01, 1.25) | 0.030 | 0.87 (0.83, 0.91) | <0.001 | 0.97 (0.89, 1.07) | 0.544 | 1.14 (1.04, 1.25) | 0.004 |
| **Triglycerides** | |  |  |  |  |  |  |  |  |  |  |  |
| Quintile 1 | Reference | | Reference | | Reference | | Reference | | Reference | | Reference | |
| Quintile 2 | 0.86 (0.77, 0.97) | 0.010 | 0.82 (0.59, 1.15) | 0.258 | 0.81 (0.59, 1.12) | 0.204 | 0.87 (0.77, 0.98) | 0.022 | 0.81 (0.64, 1.02) | 0.073 | 0.85 (0.67, 1.08) | 0.187 |
| Quintile 3 | 0.75 (0.67, 0.85) | <0.001 | 0.80 (0.57, 1.12) | 0.186 | 0.91 (0.67, 1.25) | 0.565 | 0.79 (0.70, 0.90) | <0.001 | 0.64 (0.50, 0.81) | <0.001 | 0.75 (0.59, 0.96) | 0.020 |
| Quintile 4 | 0.74 (0.66, 0.84) | <0.001 | 0.86 (0.61, 1.20) | 0.370 | 0.89 (0.65, 1.21) | 0.467 | 0.75 (0.65, 0.85) | <0.001 | 0.75 (0.60, 0.95) | 0.016 | 0.90 (0.71, 1.13) | 0.350 |
| Quintile 5 | 0.68 (0.60, 0.78) | <0.001 | 0.75 (0.53, 1.07) | 0.116 | 0.92 (0.67, 1.26) | 0.611 | 0.66 (0.57, 0.77) | <0.001 | 0.74 (0.58, 0.93) | 0.012 | 1.02 (0.81, 1.29) | 0.870 |
| Per 1-SD increase | 0.89 (0.85, 0.93) | <0.001 | 0.88 (0.78, 0.99) | 0.033 | 0.94 (0.84, 1.05) | 0.290 | 0.87 (0.82, 0.92) | <0.001 | 0.93 (0.86, 1.00) | 0.053 | 1.04 (0.96, 1.13) | 0.289 |
| **Lipoprotein A** | |  |  |  |  |  |  |  |  |  |  |  |
| Quintile 1 | Reference | | Reference | | Reference | | Reference | | Reference | | Reference | |
| Quintile 2 | 0.96 (0.85, 1.08) | 0.459 | 1.06 (0.75, 1.50) | 0.721 | 1.07 (0.76, 1.50) | 0.712 | 0.94 (0.83, 1.07) | 0.327 | 1.07 (0.85, 1.36) | 0.559 | 1.17 (0.92, 1.50) | 0.205 |
| Quintile 3 | 0.94 (0.84, 1.06) | 0.320 | 1.03 (0.73, 1.45) | 0.884 | 1.14 (0.81, 1.59) | 0.454 | 0.91 (0.80, 1.03) | 0.131 | 1.09 (0.87, 1.38) | 0.444 | 1.19 (0.93, 1.52) | 0.169 |
| Quintile 4 | 0.95 (0.85, 1.07) | 0.417 | 1.10 (0.78, 1.54) | 0.582 | 1.15 (0.82, 1.60) | 0.425 | 0.91 (0.80, 1.03) | 0.145 | 1.17 (0.93, 1.46) | 0.186 | 1.22 (0.95, 1.55) | 0.115 |
| Quintile 5 | 0.94 (0.83, 1.06) | 0.291 | 1.11 (0.81, 1.53) | 0.502 | 1.24 (0.91, 1.69) | 0.176 | 0.92 (0.81, 1.05) | 0.213 | 1.08 (0.87, 1.35) | 0.476 | 1.19 (0.95, 1.51) | 0.136 |
| Per 1-SD increase | 0.99 (0.95, 1.03) | 0.553 | 1.03 (0.94, 1.12) | 0.585 | 1.05 (0.96, 1.15) | 0.267 | 0.98 (0.94, 1.03) | 0.468 | 1.01 (0.95, 1.08) | 0.663 | 1.03 (0.96, 1.11) | 0.357 |

Notes: CVD = Cardiovascular disease; LLM = Lipid-lowering medications; HR = Hazard ratio; CI = Confidence interval; HDL = High density lipoprotein; LDL = Low density lipoprotein. Model was adjusted for age, sex (excluded in the model stratified by sex), ethnicity, deprivation index, current smokers, alcohol intake, sleep duration, total physical activity, total sedentary behavior, fruit and vegetables intake, red meat intake, processed meat intake, oily fish intake, ever eats eggs, ever eats dairy, body-mass index, waist-hip ratio, body fat percentage, number of morbidities, prefrail/frail status, CVD (excluded in the model stratified by CVD), hypertension, diabetes , chronic kidney disease, cancer, anemia, positive rheumatoid factor, vitamin D deficiency, osteoporosis, falls in the last year, lipid-lowering medications (excluded in the model stratified by use of LLM), aspirin, glucocorticoids, vitamin D supplements, and calcium supplements.

# Supplementary Table 20. Associations between serum lipid concentrations and the risk of clinical vertebral fractures in the UK Biobank, stratified by age or sex.

| **Lipid traits** | **Age ≥ 60 years (ref)** | | **Age < 60 years** | | **Ratio of HR  (95% CI)** | **P-value** | **Women (ref)** | | **Men** | | **Ratio of HR  (95% CI)** | **P-value** |
| --- | --- | --- | --- | --- | --- | --- | --- | --- | --- | --- | --- | --- |
|  | **HR (95% CI)** | **P-value** | **HR (95% CI)** | **P-value** |  |  | **HR (95% CI)** | **P-value** | **HR (95% CI)** | **P-value** |  |  |
| **Apolipoprotein A** | |  |  |  |  |  |  |  |  |  |  |  |
| Quintile 1 | Reference | | Reference | | Reference | | Reference | | Reference | | Reference | |
| Quintile 2 | 0.93 (0.79, 1.09) | 0.348 | 1.05 (0.85, 1.29) | 0.664 | 1.07 (0.84, 1.36) | 0.572 | 0.85 (0.68, 1.07) | 0.169 | 0.99 (0.85, 1.15) | 0.857 | 1.10 (0.85, 1.41) | 0.466 |
| Quintile 3 | 0.99 (0.84, 1.16) | 0.900 | 1.17 (0.95, 1.44) | 0.139 | 1.06 (0.84, 1.35) | 0.604 | 0.89 (0.72, 1.10) | 0.281 | 1.08 (0.92, 1.27) | 0.343 | 1.12 (0.88, 1.42) | 0.363 |
| Quintile 4 | 1.09 (0.93, 1.28) | 0.293 | 1.41 (1.14, 1.74) | 0.001 | 1.08 (0.86, 1.36) | 0.513 | 1.00 (0.82, 1.23) | 0.965 | 1.20 (1.01, 1.43) | 0.042 | 1.21 (0.95, 1.54) | 0.118 |
| Quintile 5 | 1.17 (0.99, 1.38) | 0.067 | 1.32 (1.05, 1.65) | 0.019 | 1.03 (0.82, 1.29) | 0.816 | 0.91 (0.74, 1.11) | 0.349 | 1.53 (1.27, 1.86) | <0.001 | 1.55 (1.22, 1.98) | <0.001 |
| Per 1-SD increase | 1.07 (1.02, 1.13) | 0.009 | 1.14 (1.06, 1.22) | <0.001 | 1.02 (0.95, 1.09) | 0.590 | 1.00 (0.95, 1.06) | 0.938 | 1.17 (1.10, 1.25) | <0.001 | 1.13 (1.05, 1.22) | <0.001 |
| **Apolipoprotein B** | |  |  |  |  |  |  |  |  |  |  |  |
| Quintile 1 | Reference | | Reference | | Reference | | Reference | | Reference | | Reference | |
| Quintile 2 | 0.90 (0.78, 1.05) | 0.175 | 0.90 (0.74, 1.09) | 0.295 | 0.98 (0.78, 1.23) | 0.870 | 0.90 (0.76, 1.07) | 0.237 | 0.89 (0.76, 1.05) | 0.159 | 1.02 (0.82, 1.26) | 0.877 |
| Quintile 3 | 0.89 (0.76, 1.04) | 0.131 | 0.92 (0.76, 1.13) | 0.438 | 1.00 (0.79, 1.25) | 0.967 | 0.88 (0.74, 1.05) | 0.158 | 0.89 (0.75, 1.06) | 0.185 | 1.10 (0.89, 1.36) | 0.383 |
| Quintile 4 | 0.93 (0.79, 1.09) | 0.351 | 0.91 (0.74, 1.11) | 0.359 | 1.05 (0.84, 1.32) | 0.669 | 0.91 (0.77, 1.09) | 0.309 | 0.88 (0.74, 1.05) | 0.167 | 1.05 (0.84, 1.30) | 0.685 |
| Quintile 5 | 0.83 (0.71, 0.98) | 0.026 | 0.78 (0.63, 0.97) | 0.025 | 0.98 (0.77, 1.23) | 0.839 | 0.76 (0.63, 0.92) | 0.004 | 0.83 (0.69, 1.00) | 0.045 | 1.19 (0.95, 1.48) | 0.125 |
| Per 1-SD increase | 0.95 (0.90, 1.00) | 0.039 | 0.93 (0.87, 1.00) | 0.049 | 1.01 (0.93, 1.08) | 0.895 | 0.93 (0.87, 0.98) | 0.010 | 0.94 (0.88, 1.00) | 0.044 | 1.05 (0.98, 1.12) | 0.209 |
| **Total cholesterol** | |  |  |  |  |  |  |  |  |  |  |  |
| Quintile 1 | Reference | | Reference | | Reference | | Reference | | Reference | | Reference | |
| Quintile 2 | 1.14 (0.98, 1.32) | 0.082 | 0.92 (0.75, 1.13) | 0.425 | 0.82 (0.65, 1.03) | 0.083 | 1.00 (0.83, 1.21) | 0.965 | 1.07 (0.91, 1.25) | 0.417 | 1.10 (0.88, 1.36) | 0.404 |
| Quintile 3 | 1.00 (0.85, 1.18) | 0.987 | 1.03 (0.85, 1.27) | 0.741 | 1.05 (0.83, 1.32) | 0.695 | 1.01 (0.83, 1.22) | 0.951 | 0.97 (0.81, 1.15) | 0.716 | 1.07 (0.86, 1.34) | 0.527 |
| Quintile 4 | 0.99 (0.83, 1.17) | 0.890 | 0.99 (0.80, 1.22) | 0.901 | 1.00 (0.80, 1.27) | 0.977 | 0.94 (0.78, 1.14) | 0.547 | 0.96 (0.80, 1.16) | 0.704 | 1.11 (0.89, 1.39) | 0.345 |
| Quintile 5 | 0.98 (0.82, 1.16) | 0.811 | 0.94 (0.75, 1.16) | 0.547 | 0.98 (0.77, 1.23) | 0.835 | 0.86 (0.70, 1.04) | 0.119 | 1.03 (0.85, 1.25) | 0.726 | 1.32 (1.06, 1.66) | 0.015 |
| Per 1-SD increase | 0.97 (0.92, 1.03) | 0.361 | 0.98 (0.92, 1.05) | 0.614 | 1.00 (0.93, 1.08) | 0.992 | 0.94 (0.88, 1.00) | 0.034 | 0.99 (0.93, 1.06) | 0.842 | 1.08 (1.01, 1.16) | 0.027 |
| **HDL cholesterol** | |  |  |  |  |  |  |  |  |  |  |  |
| Quintile 1 | Reference | | Reference | | Reference | | Reference | | Reference | | Reference | |
| Quintile 2 | 1.07 (0.92, 1.24) | 0.403 | 1.15 (0.93, 1.42) | 0.187 | 1.03 (0.81, 1.31) | 0.783 | 1.04 (0.83, 1.30) | 0.750 | 1.05 (0.91, 1.22) | 0.487 | 1.00 (0.78, 1.29) | 0.985 |
| Quintile 3 | 1.10 (0.94, 1.29) | 0.221 | 1.32 (1.07, 1.64) | 0.010 | 1.08 (0.85, 1.36) | 0.548 | 1.03 (0.83, 1.28) | 0.801 | 1.17 (1.00, 1.38) | 0.052 | 1.11 (0.87, 1.42) | 0.416 |
| Quintile 4 | 1.18 (1.00, 1.39) | 0.052 | 1.47 (1.18, 1.83) | <0.001 | 1.04 (0.83, 1.32) | 0.726 | 1.02 (0.82, 1.27) | 0.870 | 1.40 (1.18, 1.67) | <0.001 | 1.35 (1.06, 1.73) | 0.017 |
| Quintile 5 | 1.30 (1.09, 1.54) | 0.004 | 1.49 (1.17, 1.90) | 0.001 | 0.98 (0.78, 1.24) | 0.883 | 1.08 (0.87, 1.35) | 0.478 | 1.53 (1.25, 1.89) | <0.001 | 1.40 (1.09, 1.81) | 0.009 |
| Per 1-SD increase | 1.09 (1.04, 1.16) | 0.001 | 1.16 (1.08, 1.25) | <0.001 | 1.00 (0.94, 1.07) | 0.933 | 1.03 (0.97, 1.09) | 0.409 | 1.21 (1.13, 1.29) | <0.001 | 1.14 (1.06, 1.23) | <0.001 |
| **LDL cholesterol** | |  |  |  |  |  |  |  |  |  |  |  |
| Quintile 1 | Reference | | Reference | | Reference | | Reference | | Reference | | Reference | |
| Quintile 2 | 0.98 (0.84, 1.13) | 0.778 | 0.94 (0.77, 1.15) | 0.570 | 0.95 (0.76, 1.19) | 0.684 | 0.90 (0.75, 1.07) | 0.214 | 1.01 (0.86, 1.19) | 0.904 | 1.16 (0.94, 1.44) | 0.162 |
| Quintile 3 | 0.98 (0.83, 1.14) | 0.755 | 0.96 (0.78, 1.18) | 0.689 | 0.94 (0.75, 1.18) | 0.609 | 0.91 (0.76, 1.09) | 0.306 | 0.98 (0.82, 1.17) | 0.844 | 1.18 (0.95, 1.46) | 0.139 |
| Quintile 4 | 0.84 (0.71, 1.00) | 0.046 | 0.97 (0.79, 1.20) | 0.800 | 1.16 (0.92, 1.46) | 0.221 | 0.84 (0.70, 1.01) | 0.065 | 0.88 (0.73, 1.07) | 0.197 | 1.12 (0.90, 1.40) | 0.300 |
| Quintile 5 | 0.90 (0.76, 1.07) | 0.246 | 0.86 (0.69, 1.07) | 0.177 | 0.96 (0.76, 1.21) | 0.709 | 0.77 (0.64, 0.93) | 0.008 | 0.98 (0.81, 1.18) | 0.802 | 1.35 (1.08, 1.68) | 0.007 |
| Per 1-SD increase | 0.96 (0.90, 1.01) | 0.103 | 0.95 (0.88, 1.02) | 0.126 | 0.99 (0.92, 1.07) | 0.827 | 0.93 (0.87, 0.98) | 0.012 | 0.96 (0.90, 1.02) | 0.204 | 1.07 (1.00, 1.15) | 0.054 |
| **Triglycerides** | |  |  |  |  |  |  |  |  |  |  |  |
| Quintile 1 | Reference | | Reference | | Reference | | Reference | | Reference | | Reference | |
| Quintile 2 | 0.91 (0.78, 1.07) | 0.254 | 0.90 (0.74, 1.09) | 0.272 | 1.06 (0.84, 1.33) | 0.637 | 0.92 (0.78, 1.08) | 0.309 | 0.86 (0.71, 1.03) | 0.098 | 0.90 (0.72, 1.13) | 0.365 |
| Quintile 3 | 0.85 (0.73, 0.99) | 0.036 | 0.87 (0.71, 1.07) | 0.190 | 1.15 (0.91, 1.44) | 0.238 | 0.85 (0.72, 1.01) | 0.064 | 0.81 (0.68, 0.97) | 0.021 | 0.91 (0.73, 1.14) | 0.432 |
| Quintile 4 | 0.81 (0.69, 0.95) | 0.009 | 0.95 (0.78, 1.17) | 0.633 | 1.32 (1.05, 1.65) | 0.017 | 0.83 (0.69, 0.99) | 0.034 | 0.83 (0.69, 0.98) | 0.033 | 0.93 (0.74, 1.16) | 0.497 |
| Quintile 5 | 0.75 (0.64, 0.89) | <0.001 | 0.76 (0.61, 0.94) | 0.012 | 1.18 (0.94, 1.49) | 0.149 | 0.76 (0.63, 0.93) | 0.007 | 0.72 (0.60, 0.86) | <0.001 | 0.83 (0.66, 1.05) | 0.119 |
| Per 1-SD increase | 0.91 (0.86, 0.96) | <0.001 | 0.91 (0.85, 0.98) | 0.011 | 1.05 (0.98, 1.14) | 0.167 | 0.92 (0.86, 0.98) | 0.016 | 0.91 (0.86, 0.96) | <0.001 | 0.94 (0.87, 1.02) | 0.120 |
| **Lipoprotein A** | |  |  |  |  |  |  |  |  |  |  |  |
| Quintile 1 | Reference | | Reference | | Reference | | Reference | | Reference | | Reference | |
| Quintile 2 | 1.05 (0.90, 1.22) | 0.544 | 1.04 (0.86, 1.25) | 0.707 | 1.02 (0.82, 1.28) | 0.842 | 0.89 (0.75, 1.06) | 0.192 | 1.18 (1.01, 1.38) | 0.040 | 1.29 (1.04, 1.61) | 0.022 |
| Quintile 3 | 1.08 (0.93, 1.25) | 0.310 | 0.88 (0.72, 1.07) | 0.204 | 0.82 (0.65, 1.03) | 0.089 | 0.99 (0.84, 1.17) | 0.916 | 0.98 (0.83, 1.16) | 0.802 | 0.99 (0.79, 1.23) | 0.909 |
| Quintile 4 | 1.10 (0.95, 1.27) | 0.207 | 0.88 (0.72, 1.07) | 0.205 | 0.78 (0.62, 0.98) | 0.034 | 0.95 (0.80, 1.12) | 0.544 | 1.06 (0.90, 1.26) | 0.474 | 1.09 (0.87, 1.36) | 0.440 |
| Quintile 5 | 0.98 (0.84, 1.14) | 0.787 | 0.97 (0.80, 1.18) | 0.785 | 0.96 (0.76, 1.20) | 0.692 | 0.90 (0.76, 1.07) | 0.226 | 1.03 (0.87, 1.22) | 0.715 | 1.12 (0.90, 1.39) | 0.314 |
| Per 1-SD increase | 0.98 (0.93, 1.02) | 0.359 | 0.99 (0.93, 1.05) | 0.739 | 0.99 (0.92, 1.07) | 0.804 | 0.98 (0.93, 1.03) | 0.381 | 0.99 (0.93, 1.04) | 0.606 | 1.00 (0.94, 1.08) | 0.900 |

Notes: HR = Hazard ratio; CI = Confidence interval; HDL = High density lipoprotein; LDL = Low density lipoprotein. Model was adjusted for age (excluded in the model stratified by age), sex (excluded in the model stratified by sex), ethnicity, deprivation index, current smokers, alcohol intake, sleep duration, total physical activity, total sedentary behavior, fruit and vegetables intake, red meat intake, processed meat intake, oily fish intake, ever eats eggs, ever eats dairy, body-mass index, waist-hip ratio, body fat percentage, number of morbidities, prefrail/frail status, cardiovascular disease, hypertension, diabetes , chronic kidney disease, cancer, anemia, positive rheumatoid factor, vitamin D deficiency, osteoporosis, falls in the last year, menopause status (included in the model for women), lipid-lowering medications, aspirin, glucocorticoids, vitamin D supplements, and calcium supplements.

# Supplementary Table 21. Associations between serum lipid concentrations and the risk of clinical vertebral fractures in the UK Biobank, stratified by BMI.

| **Lipid traits** | **Normal weight (ref)** | | **Overweight** | | **Ratio of HR  (95% CI)** | **P-value** | **Obese** | | **Ratio of HR  (95% CI)** | **P-value** |
| --- | --- | --- | --- | --- | --- | --- | --- | --- | --- | --- |
|  | **HR (95% CI)** | **P-value** | **HR (95% CI)** | **P-value** |  |  | **HR (95% CI)** | **P-value** |  |  |
| **Apolipoprotein A** |  |  |  |  |  |  |  |  |  |  |
| Quintile 1 | Reference | | Reference | | Reference | | Reference | | Reference | |
| Quintile 2 | 0.99 (0.75, 1.31) | 0.951 | 1.01 (0.84, 1.21) | 0.928 | 1.05 (0.77, 1.42) | 0.778 | 0.84 (0.67, 1.04) | 0.111 | 0.88 (0.64, 1.22) | 0.458 |
| Quintile 3 | 1.06 (0.81, 1.37) | 0.686 | 1.02 (0.84, 1.23) | 0.849 | 1.03 (0.77, 1.38) | 0.849 | 1.01 (0.81, 1.27) | 0.902 | 0.95 (0.69, 1.30) | 0.736 |
| Quintile 4 | 1.10 (0.85, 1.43) | 0.451 | 1.14 (0.94, 1.39) | 0.194 | 1.08 (0.81, 1.43) | 0.609 | 1.20 (0.94, 1.52) | 0.138 | 1.13 (0.83, 1.53) | 0.436 |
| Quintile 5 | 1.03 (0.79, 1.34) | 0.844 | 1.29 (1.05, 1.59) | 0.015 | 1.19 (0.91, 1.58) | 0.208 | 1.20 (0.91, 1.57) | 0.196 | 1.20 (0.88, 1.65) | 0.252 |
| Per 1-SD increase | 1.01 (0.94, 1.08) | 0.875 | 1.10 (1.03, 1.18) | 0.004 | 1.06 (0.98, 1.14) | 0.176 | 1.13 (1.03, 1.24) | 0.008 | 1.10 (1.00, 1.21) | 0.042 |
| **Apolipoprotein B** |  |  |  |  |  |  |  |  |  |  |
| Quintile 1 | Reference | | Reference | | Reference | | Reference | | Reference | |
| Quintile 2 | 0.91 (0.74, 1.11) | 0.335 | 0.88 (0.73, 1.06) | 0.187 | 0.95 (0.74, 1.21) | 0.675 | 0.87 (0.69, 1.11) | 0.262 | 0.98 (0.74, 1.29) | 0.869 |
| Quintile 3 | 0.91 (0.74, 1.12) | 0.372 | 0.91 (0.76, 1.10) | 0.344 | 1.03 (0.80, 1.31) | 0.833 | 0.84 (0.65, 1.08) | 0.184 | 0.96 (0.72, 1.28) | 0.787 |
| Quintile 4 | 0.83 (0.67, 1.04) | 0.100 | 0.86 (0.71, 1.05) | 0.135 | 1.09 (0.85, 1.40) | 0.505 | 1.07 (0.84, 1.37) | 0.597 | 1.37 (1.03, 1.82) | 0.028 |
| Quintile 5 | 0.71 (0.56, 0.90) | 0.005 | 0.76 (0.62, 0.93) | 0.007 | 1.08 (0.83, 1.42) | 0.555 | 0.97 (0.75, 1.25) | 0.799 | 1.39 (1.04, 1.87) | 0.028 |
| Per 1-SD increase | 0.88 (0.81, 0.95) | 0.001 | 0.93 (0.87, 0.99) | 0.030 | 1.06 (0.97, 1.15) | 0.213 | 1.01 (0.93, 1.09) | 0.898 | 1.15 (1.04, 1.26) | 0.004 |
| **Total cholesterol** |  |  |  |  |  |  |  |  |  |  |
| Quintile 1 | Reference | | Reference | | Reference | | Reference | | Reference | |
| Quintile 2 | 0.97 (0.78, 1.21) | 0.789 | 1.14 (0.95, 1.38) | 0.165 | 1.12 (0.87, 1.46) | 0.377 | 0.98 (0.78, 1.24) | 0.895 | 1.05 (0.79, 1.40) | 0.730 |
| Quintile 3 | 1.01 (0.81, 1.27) | 0.906 | 1.05 (0.86, 1.28) | 0.653 | 1.03 (0.80, 1.34) | 0.804 | 0.90 (0.69, 1.16) | 0.408 | 0.90 (0.67, 1.21) | 0.496 |
| Quintile 4 | 0.96 (0.76, 1.21) | 0.728 | 0.93 (0.75, 1.15) | 0.499 | 0.95 (0.74, 1.24) | 0.726 | 1.03 (0.79, 1.33) | 0.840 | 1.07 (0.80, 1.42) | 0.661 |
| Quintile 5 | 0.79 (0.62, 1.01) | 0.063 | 0.94 (0.76, 1.16) | 0.560 | 1.17 (0.90, 1.53) | 0.241 | 1.12 (0.86, 1.45) | 0.410 | 1.48 (1.11, 1.98) | 0.007 |
| Per 1-SD increase | 0.91 (0.84, 0.98) | 0.019 | 0.96 (0.90, 1.03) | 0.239 | 1.04 (0.95, 1.12) | 0.400 | 1.03 (0.95, 1.13) | 0.427 | 1.13 (1.03, 1.23) | 0.010 |
| **HDL cholesterol** |  |  |  |  |  |  |  |  |  |  |
| Quintile 1 | Reference | | Reference | | Reference | | Reference | | Reference | |
| Quintile 2 | 1.04 (0.78, 1.40) | 0.772 | 1.11 (0.93, 1.34) | 0.244 | 1.02 (0.74, 1.41) | 0.901 | 1.03 (0.84, 1.27) | 0.757 | 0.99 (0.71, 1.39) | 0.970 |
| Quintile 3 | 1.08 (0.81, 1.43) | 0.613 | 1.14 (0.94, 1.38) | 0.172 | 1.07 (0.79, 1.46) | 0.655 | 1.18 (0.94, 1.48) | 0.152 | 1.10 (0.80, 1.53) | 0.557 |
| Quintile 4 | 1.16 (0.88, 1.53) | 0.300 | 1.30 (1.06, 1.58) | 0.010 | 1.11 (0.83, 1.50) | 0.476 | 1.10 (0.85, 1.43) | 0.475 | 0.96 (0.69, 1.33) | 0.788 |
| Quintile 5 | 1.14 (0.86, 1.51) | 0.366 | 1.33 (1.07, 1.65) | 0.011 | 1.05 (0.78, 1.41) | 0.764 | 1.56 (1.17, 2.08) | 0.003 | 1.35 (0.97, 1.89) | 0.080 |
| Per 1-SD increase | 1.02 (0.95, 1.10) | 0.502 | 1.12 (1.04, 1.20) | 0.002 | 1.03 (0.95, 1.12) | 0.450 | 1.20 (1.09, 1.33) | <0.001 | 1.12 (1.02, 1.24) | 0.016 |
| **LDL cholesterol** |  |  |  |  |  |  |  |  |  |  |
| Quintile 1 | Reference | | Reference | | Reference | | Reference | | Reference | |
| Quintile 2 | 0.90 (0.73, 1.11) | 0.327 | 0.99 (0.82, 1.20) | 0.934 | 1.04 (0.81, 1.33) | 0.785 | 1.01 (0.80, 1.27) | 0.958 | 1.07 (0.81, 1.41) | 0.647 |
| Quintile 3 | 0.93 (0.75, 1.16) | 0.529 | 1.02 (0.84, 1.24) | 0.826 | 1.03 (0.81, 1.32) | 0.799 | 0.89 (0.68, 1.15) | 0.362 | 0.91 (0.68, 1.21) | 0.506 |
| Quintile 4 | 0.88 (0.70, 1.10) | 0.260 | 0.78 (0.63, 0.97) | 0.024 | 0.96 (0.74, 1.24) | 0.754 | 1.03 (0.79, 1.34) | 0.826 | 1.23 (0.92, 1.63) | 0.163 |
| Quintile 5 | 0.73 (0.57, 0.93) | 0.010 | 0.88 (0.71, 1.08) | 0.213 | 1.16 (0.89, 1.51) | 0.282 | 1.06 (0.81, 1.37) | 0.684 | 1.47 (1.10, 1.97) | 0.009 |
| Per 1-SD increase | 0.90 (0.83, 0.97) | 0.007 | 0.93 (0.87, 1.00) | 0.042 | 1.04 (0.96, 1.13) | 0.367 | 1.02 (0.93, 1.11) | 0.720 | 1.13 (1.03, 1.24) | 0.008 |
| **Triglycerides** |  |  |  |  |  |  |  |  |  |  |
| Quintile 1 | Reference | | Reference | | Reference | | Reference | | Reference | |
| Quintile 2 | 0.90 (0.75, 1.08) | 0.268 | 0.89 (0.73, 1.08) | 0.228 | 1.00 (0.79, 1.28) | 0.994 | 0.85 (0.62, 1.17) | 0.325 | 0.96 (0.68, 1.34) | 0.793 |
| Quintile 3 | 0.86 (0.71, 1.05) | 0.144 | 0.82 (0.67, 0.99) | 0.041 | 0.98 (0.77, 1.26) | 0.903 | 0.82 (0.60, 1.11) | 0.201 | 0.95 (0.68, 1.33) | 0.778 |
| Quintile 4 | 0.87 (0.70, 1.08) | 0.197 | 0.77 (0.63, 0.93) | 0.008 | 0.98 (0.75, 1.27) | 0.866 | 0.87 (0.65, 1.17) | 0.364 | 1.05 (0.76, 1.47) | 0.760 |
| Quintile 5 | 0.90 (0.70, 1.16) | 0.431 | 0.69 (0.57, 0.84) | <0.001 | 0.78 (0.58, 1.03) | 0.080 | 0.72 (0.54, 0.97) | 0.028 | 0.81 (0.57, 1.14) | 0.229 |
| Per 1-SD increase | 0.95 (0.87, 1.05) | 0.310 | 0.91 (0.86, 0.97) | 0.006 | 0.97 (0.87, 1.07) | 0.503 | 0.89 (0.83, 0.96) | 0.002 | 0.97 (0.87, 1.08) | 0.602 |
| **Lipoprotein A** |  |  |  |  |  |  |  |  |  |  |
| Quintile 1 | Reference | | Reference | | Reference | | Reference | | Reference | |
| Quintile 2 | 1.05 (0.86, 1.29) | 0.616 | 1.11 (0.92, 1.34) | 0.276 | 1.06 (0.82, 1.37) | 0.659 | 0.92 (0.74, 1.15) | 0.487 | 0.88 (0.66, 1.17) | 0.384 |
| Quintile 3 | 0.96 (0.78, 1.18) | 0.710 | 1.15 (0.96, 1.39) | 0.132 | 1.19 (0.92, 1.55) | 0.179 | 0.83 (0.66, 1.05) | 0.126 | 0.93 (0.70, 1.24) | 0.629 |
| Quintile 4 | 0.92 (0.74, 1.13) | 0.424 | 1.17 (0.97, 1.41) | 0.098 | 1.27 (0.98, 1.65) | 0.076 | 0.92 (0.73, 1.15) | 0.469 | 1.02 (0.76, 1.36) | 0.917 |
| Quintile 5 | 0.88 (0.71, 1.09) | 0.251 | 1.11 (0.93, 1.34) | 0.252 | 1.21 (0.93, 1.57) | 0.152 | 0.89 (0.71, 1.11) | 0.291 | 1.05 (0.79, 1.40) | 0.725 |
| Per 1-SD increase | 0.97 (0.91, 1.04) | 0.374 | 0.99 (0.94, 1.05) | 0.761 | 1.02 (0.94, 1.11) | 0.622 | 0.98 (0.91, 1.06) | 0.627 | 1.04 (0.95, 1.14) | 0.414 |

Notes: HR = Hazard ratio; CI = Confidence interval; HDL = High density lipoprotein; LDL = Low density lipoprotein. Model was adjusted for age, sex, ethnicity, deprivation index, current smokers, alcohol intake, sleep duration, total physical activity, total sedentary behavior, fruit and vegetables intake, red meat intake, processed meat intake, oily fish intake, ever eats eggs, ever eats dairy, waist-hip ratio, body fat percentage, number of morbidities, prefrail/frail status, cardiovascular disease, hypertension, diabetes , chronic kidney disease, cancer, anemia, positive rheumatoid factor, vitamin D deficiency, osteoporosis, falls in the last year, lipid-lowering medications, aspirin, glucocorticoids, vitamin D supplements, and calcium supplements.

# Supplementary Table 22. Associations between serum lipid concentrations and the risk of clinical vertebral fractures in the UK Biobank, stratified by CVD or use of lipid-lowering medications.

| **Lipid traits** | **Non-CVD (ref)** | | **CVD** | | **Ratio of HR  (95% CI)** | **P-value** | **Not taking LLM (ref)** | | **Taking LLM** | | **Ratio of HR  (95% CI)** | **P-value** |
| --- | --- | --- | --- | --- | --- | --- | --- | --- | --- | --- | --- | --- |
|  | **HR (95% CI)** | **P-value** | **HR (95% CI)** | **P-value** |  |  | **HR (95% CI)** | **P-value** | **HR (95% CI)** | **P-value** |  |  |
| **Apolipoprotein A** | |  |  |  |  |  |  |  |  |  |  |  |
| Quintile 1 | Reference | | Reference | | Reference | | Reference | | Reference | | Reference | |
| Quintile 2 | 0.97 (0.85, 1.11) | 0.672 | 0.86 (0.62, 1.18) | 0.342 | 0.86 (0.63, 1.19) | 0.374 | 0.94 (0.81, 1.09) | 0.404 | 0.99 (0.79, 1.25) | 0.961 | 1.07 (0.83, 1.37) | 0.624 |
| Quintile 3 | 1.03 (0.89, 1.18) | 0.717 | 1.07 (0.77, 1.48) | 0.706 | 1.03 (0.75, 1.42) | 0.853 | 1.08 (0.93, 1.25) | 0.318 | 0.86 (0.67, 1.10) | 0.227 | 0.88 (0.68, 1.14) | 0.331 |
| Quintile 4 | 1.16 (1.01, 1.33) | 0.034 | 1.09 (0.76, 1.56) | 0.646 | 1.01 (0.73, 1.40) | 0.956 | 1.10 (0.95, 1.28) | 0.219 | 1.32 (1.04, 1.68) | 0.025 | 1.27 (0.99, 1.63) | 0.056 |
| Quintile 5 | 1.16 (1.01, 1.35) | 0.041 | 1.08 (0.73, 1.60) | 0.707 | 0.93 (0.66, 1.32) | 0.697 | 1.12 (0.96, 1.32) | 0.152 | 1.27 (0.98, 1.65) | 0.075 | 1.14 (0.88, 1.46) | 0.319 |
| Per 1-SD increase | 1.08 (1.03, 1.13) | 0.001 | 1.03 (0.91, 1.17) | 0.650 | 0.98 (0.88, 1.09) | 0.660 | 1.06 (1.01, 1.11) | 0.025 | 1.12 (1.03, 1.22) | 0.009 | 1.05 (0.98, 1.14) | 0.181 |
| **Apolipoprotein B** | |  |  |  |  |  |  |  |  |  |  |  |
| Quintile 1 | Reference | | Reference | | Reference | | Reference | | Reference | | Reference | |
| Quintile 2 | 0.86 (0.75, 0.97) | 0.018 | 1.08 (0.82, 1.42) | 0.583 | 1.29 (0.97, 1.71) | 0.081 | 0.90 (0.78, 1.05) | 0.188 | 0.82 (0.67, 0.99) | 0.039 | 0.88 (0.70, 1.10) | 0.255 |
| Quintile 3 | 0.84 (0.74, 0.96) | 0.011 | 1.23 (0.89, 1.69) | 0.211 | 1.54 (1.12, 2.11) | 0.007 | 0.83 (0.72, 0.96) | 0.014 | 1.03 (0.82, 1.28) | 0.817 | 1.26 (0.99, 1.61) | 0.065 |
| Quintile 4 | 0.89 (0.78, 1.01) | 0.075 | 0.76 (0.49, 1.16) | 0.204 | 1.10 (0.74, 1.65) | 0.633 | 0.86 (0.75, 1.00) | 0.051 | 0.96 (0.72, 1.27) | 0.760 | 1.17 (0.87, 1.56) | 0.301 |
| Quintile 5 | 0.77 (0.67, 0.88) | <0.001 | 0.93 (0.59, 1.45) | 0.745 | 1.60 (1.07, 2.37) | 0.021 | 0.75 (0.64, 0.87) | <0.001 | 1.10 (0.79, 1.51) | 0.574 | 1.55 (1.12, 2.14) | 0.008 |
| Per 1-SD increase | 0.93 (0.89, 0.97) | 0.002 | 0.96 (0.84, 1.09) | 0.498 | 1.13 (1.01, 1.26) | 0.039 | 0.91 (0.87, 0.95) | <0.001 | 1.03 (0.94, 1.13) | 0.501 | 1.14 (1.04, 1.25) | 0.004 |
| **Total cholesterol** | |  |  |  |  |  |  |  |  |  |  |  |
| Quintile 1 | Reference | | Reference | | Reference | | Reference | | Reference | | Reference | |
| Quintile 2 | 1.04 (0.91, 1.19) | 0.577 | 1.05 (0.79, 1.40) | 0.736 | 1.11 (0.84, 1.49) | 0.457 | 0.94 (0.80, 1.10) | 0.413 | 1.16 (0.96, 1.39) | 0.125 | 1.25 (1.00, 1.57) | 0.049 |
| Quintile 3 | 0.98 (0.85, 1.12) | 0.731 | 1.16 (0.82, 1.64) | 0.398 | 1.40 (1.00, 1.96) | 0.048 | 0.92 (0.79, 1.08) | 0.302 | 1.00 (0.78, 1.28) | 0.988 | 1.16 (0.89, 1.52) | 0.275 |
| Quintile 4 | 0.95 (0.83, 1.10) | 0.518 | 0.98 (0.64, 1.51) | 0.929 | 1.34 (0.91, 1.99) | 0.140 | 0.88 (0.75, 1.03) | 0.105 | 1.06 (0.76, 1.46) | 0.745 | 1.31 (0.94, 1.81) | 0.105 |
| Quintile 5 | 0.94 (0.81, 1.09) | 0.407 | 0.62 (0.35, 1.09) | 0.095 | 1.04 (0.64, 1.67) | 0.878 | 0.84 (0.71, 0.98) | 0.028 | 1.37 (0.97, 1.92) | 0.074 | 1.72 (1.22, 2.42) | 0.002 |
| Per 1-SD increase | 0.96 (0.92, 1.01) | 0.118 | 0.97 (0.85, 1.10) | 0.616 | 1.12 (1.00, 1.25) | 0.046 | 0.93 (0.89, 0.98) | 0.006 | 1.08 (0.98, 1.18) | 0.113 | 1.17 (1.07, 1.28) | <0.001 |
| **HDL cholesterol** | |  |  |  |  |  |  |  |  |  |  |  |
| Quintile 1 | Reference | | Reference | | Reference | | Reference | | Reference | | Reference | |
| Quintile 2 | 1.07 (0.93, 1.22) | 0.354 | 1.09 (0.81, 1.48) | 0.557 | 1.09 (0.80, 1.47) | 0.594 | 1.06 (0.91, 1.24) | 0.445 | 1.09 (0.88, 1.35) | 0.416 | 1.07 (0.84, 1.36) | 0.583 |
| Quintile 3 | 1.15 (1.00, 1.31) | 0.053 | 1.11 (0.79, 1.56) | 0.559 | 0.98 (0.71, 1.36) | 0.915 | 1.19 (1.02, 1.38) | 0.028 | 0.98 (0.77, 1.25) | 0.885 | 0.94 (0.73, 1.21) | 0.634 |
| Quintile 4 | 1.21 (1.05, 1.39) | 0.009 | 1.34 (0.93, 1.93) | 0.113 | 1.11 (0.80, 1.55) | 0.523 | 1.18 (1.01, 1.38) | 0.040 | 1.37 (1.07, 1.75) | 0.013 | 1.19 (0.92, 1.52) | 0.184 |
| Quintile 5 | 1.28 (1.10, 1.49) | 0.001 | 1.24 (0.81, 1.91) | 0.327 | 1.08 (0.75, 1.55) | 0.670 | 1.25 (1.06, 1.48) | 0.009 | 1.44 (1.09, 1.91) | 0.010 | 1.18 (0.91, 1.54) | 0.212 |
| Per 1-SD increase | 1.10 (1.05, 1.15) | <0.001 | 1.07 (0.93, 1.22) | 0.353 | 0.99 (0.89, 1.11) | 0.901 | 1.09 (1.04, 1.15) | <0.001 | 1.13 (1.03, 1.23) | 0.010 | 1.04 (0.96, 1.12) | 0.360 |
| **LDL cholesterol** | |  |  |  |  |  |  |  |  |  |  |  |
| Quintile 1 | Reference | | Reference | | Reference | | Reference | | Reference | | Reference | |
| Quintile 2 | 0.94 (0.82, 1.07) | 0.355 | 1.07 (0.81, 1.41) | 0.627 | 1.30 (0.98, 1.71) | 0.069 | 0.89 (0.76, 1.03) | 0.126 | 1.00 (0.83, 1.21) | 0.989 | 1.15 (0.92, 1.44) | 0.222 |
| Quintile 3 | 0.96 (0.84, 1.10) | 0.589 | 0.77 (0.51, 1.14) | 0.191 | 0.90 (0.61, 1.33) | 0.600 | 0.89 (0.76, 1.03) | 0.122 | 0.97 (0.75, 1.25) | 0.816 | 1.16 (0.89, 1.52) | 0.279 |
| Quintile 4 | 0.86 (0.74, 0.99) | 0.033 | 1.02 (0.67, 1.56) | 0.925 | 1.71 (1.17, 2.48) | 0.005 | 0.80 (0.69, 0.93) | 0.005 | 1.02 (0.73, 1.43) | 0.887 | 1.44 (1.03, 2.00) | 0.032 |
| Quintile 5 | 0.87 (0.75, 1.00) | 0.047 | 0.80 (0.49, 1.32) | 0.383 | 1.40 (0.92, 2.15) | 0.120 | 0.79 (0.67, 0.92) | 0.003 | 1.31 (0.92, 1.86) | 0.139 | 1.77 (1.24, 2.52) | 0.002 |
| Per 1-SD increase | 0.94 (0.90, 0.99) | 0.012 | 0.94 (0.83, 1.08) | 0.400 | 1.13 (1.01, 1.27) | 0.030 | 0.91 (0.87, 0.96) | <0.001 | 1.05 (0.96, 1.15) | 0.308 | 1.17 (1.07, 1.29) | 0.001 |
| **Triglycerides** | |  |  |  |  |  |  |  |  |  |  |  |
| Quintile 1 | Reference | | Reference | | Reference | | Reference | | Reference | | Reference | |
| Quintile 2 | 0.89 (0.79, 1.02) | 0.088 | 0.91 (0.64, 1.30) | 0.605 | 1.04 (0.73, 1.48) | 0.827 | 0.92 (0.80, 1.05) | 0.211 | 0.80 (0.61, 1.05) | 0.109 | 0.89 (0.67, 1.17) | 0.397 |
| Quintile 3 | 0.86 (0.76, 0.98) | 0.027 | 0.70 (0.48, 1.01) | 0.056 | 0.87 (0.61, 1.24) | 0.427 | 0.86 (0.75, 0.99) | 0.040 | 0.75 (0.58, 0.98) | 0.037 | 0.87 (0.66, 1.14) | 0.309 |
| Quintile 4 | 0.86 (0.75, 0.98) | 0.021 | 0.72 (0.50, 1.03) | 0.075 | 0.88 (0.62, 1.24) | 0.462 | 0.81 (0.70, 0.93) | 0.003 | 0.90 (0.70, 1.15) | 0.397 | 1.13 (0.87, 1.46) | 0.363 |
| Quintile 5 | 0.75 (0.65, 0.86) | <0.001 | 0.75 (0.52, 1.08) | 0.125 | 1.09 (0.78, 1.53) | 0.620 | 0.71 (0.61, 0.83) | <0.001 | 0.82 (0.63, 1.06) | 0.127 | 1.17 (0.90, 1.51) | 0.249 |
| Per 1-SD increase | 0.91 (0.87, 0.96) | <0.001 | 0.94 (0.84, 1.05) | 0.274 | 1.04 (0.93, 1.15) | 0.523 | 0.89 (0.85, 0.94) | <0.001 | 0.96 (0.89, 1.04) | 0.346 | 1.08 (1.00, 1.17) | 0.062 |
| **Lipoprotein A** | |  |  |  |  |  |  |  |  |  |  |  |
| Quintile 1 | Reference | | Reference | | Reference | | Reference | | Reference | | Reference | |
| Quintile 2 | 1.00 (0.88, 1.13) | 0.998 | 1.48 (1.04, 2.12) | 0.032 | 1.40 (0.98, 2.00) | 0.062 | 1.02 (0.89, 1.16) | 0.822 | 1.12 (0.88, 1.42) | 0.360 | 1.08 (0.84, 1.40) | 0.546 |
| Quintile 3 | 0.97 (0.86, 1.10) | 0.634 | 1.25 (0.86, 1.81) | 0.234 | 1.21 (0.84, 1.74) | 0.304 | 1.00 (0.87, 1.14) | 0.975 | 0.98 (0.77, 1.26) | 0.896 | 0.99 (0.76, 1.29) | 0.944 |
| Quintile 4 | 0.97 (0.86, 1.10) | 0.627 | 1.41 (0.98, 2.01) | 0.062 | 1.47 (1.03, 2.09) | 0.032 | 0.98 (0.85, 1.12) | 0.733 | 1.12 (0.88, 1.41) | 0.363 | 1.14 (0.88, 1.47) | 0.323 |
| Quintile 5 | 0.97 (0.85, 1.10) | 0.619 | 1.04 (0.73, 1.49) | 0.829 | 1.10 (0.77, 1.56) | 0.600 | 0.98 (0.85, 1.12) | 0.732 | 0.97 (0.77, 1.22) | 0.784 | 1.01 (0.79, 1.29) | 0.956 |
| Per 1-SD increase | 0.99 (0.95, 1.03) | 0.489 | 0.96 (0.86, 1.06) | 0.370 | 0.99 (0.90, 1.09) | 0.829 | 0.98 (0.94, 1.03) | 0.505 | 0.98 (0.91, 1.05) | 0.510 | 1.00 (0.93, 1.08) | 0.967 |

Notes: CVD = Cardiovascular disease; LLM = Lipid-lowering medications; HR = Hazard ratio; CI = Confidence interval; HDL = High density lipoprotein; LDL = Low density lipoprotein. Model was adjusted for age, sex (excluded in the model stratified by sex), ethnicity, deprivation index, current smokers, alcohol intake, sleep duration, total physical activity, total sedentary behavior, fruit and vegetables intake, red meat intake, processed meat intake, oily fish intake, ever eats eggs, ever eats dairy, body-mass index, waist-hip ratio, body fat percentage, number of morbidities, prefrail/frail status, CVD (excluded in the model stratified by CVD), hypertension, diabetes , chronic kidney disease, cancer, anemia, positive rheumatoid factor, vitamin D deficiency, osteoporosis, falls in the last year, lipid-lowering medications (excluded in the model stratified by use of LLM), aspirin, glucocorticoids, vitamin D supplements, and calcium supplements.

# Supplementary Figure 1. The flow diagram for exclusion and inclusion.


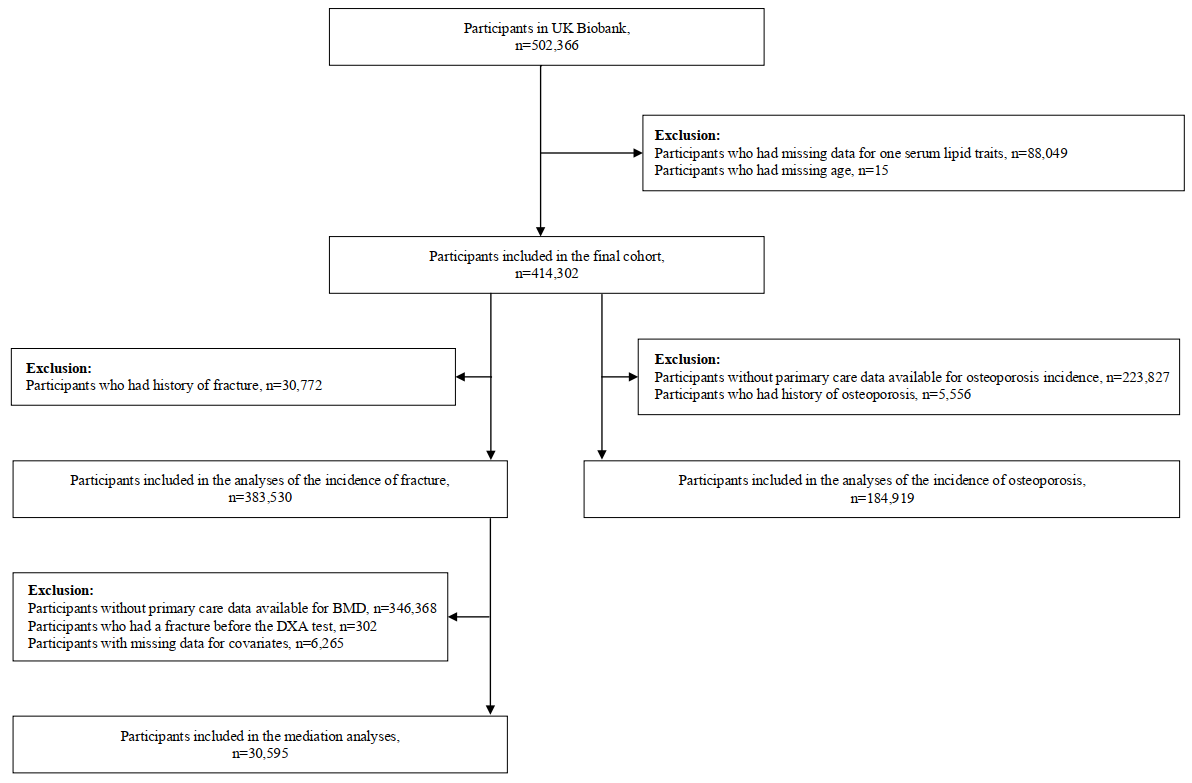


Notes: BMD = Bone Mineral Density; DXA = Dual-energy X-ray Absorptiometry.

# Supplemental Figure 2. Directed acyclic graph (DAG) of the mediation analysis


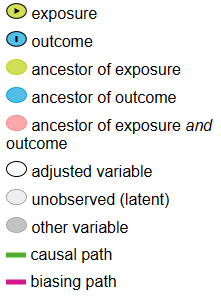

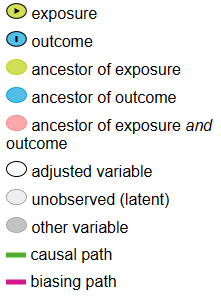


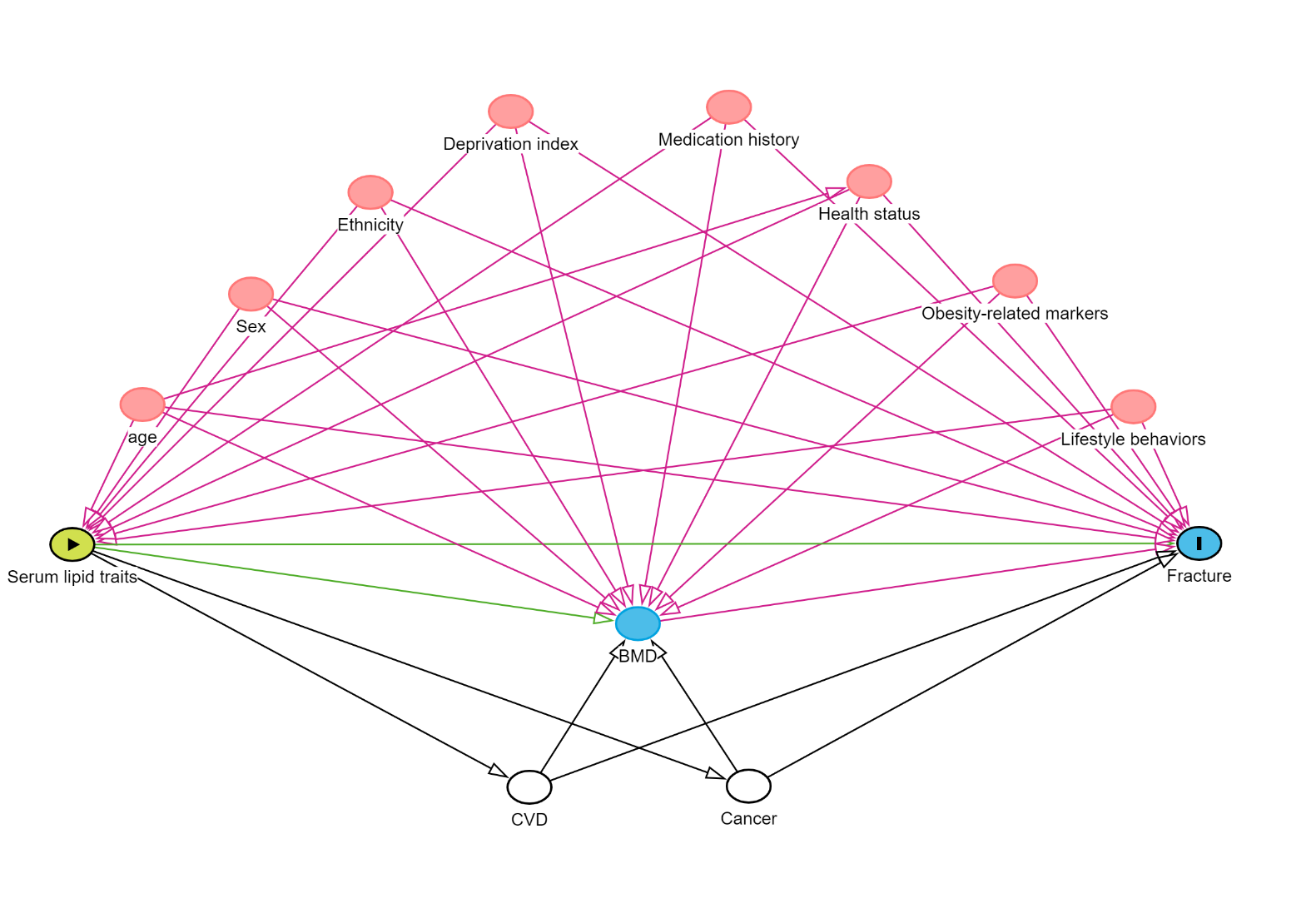

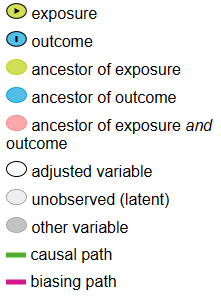


Notes: CVD = Cardiovascular disease; BMD = Bone mineral density. Cardiovascular disease and cancer were accounted as post-exposure confounders.

# Supplementary Figure 3. Associations between lipid traits and the risk of (A) fracture and (B) osteoporosis in minimally adjusted models.

(A)


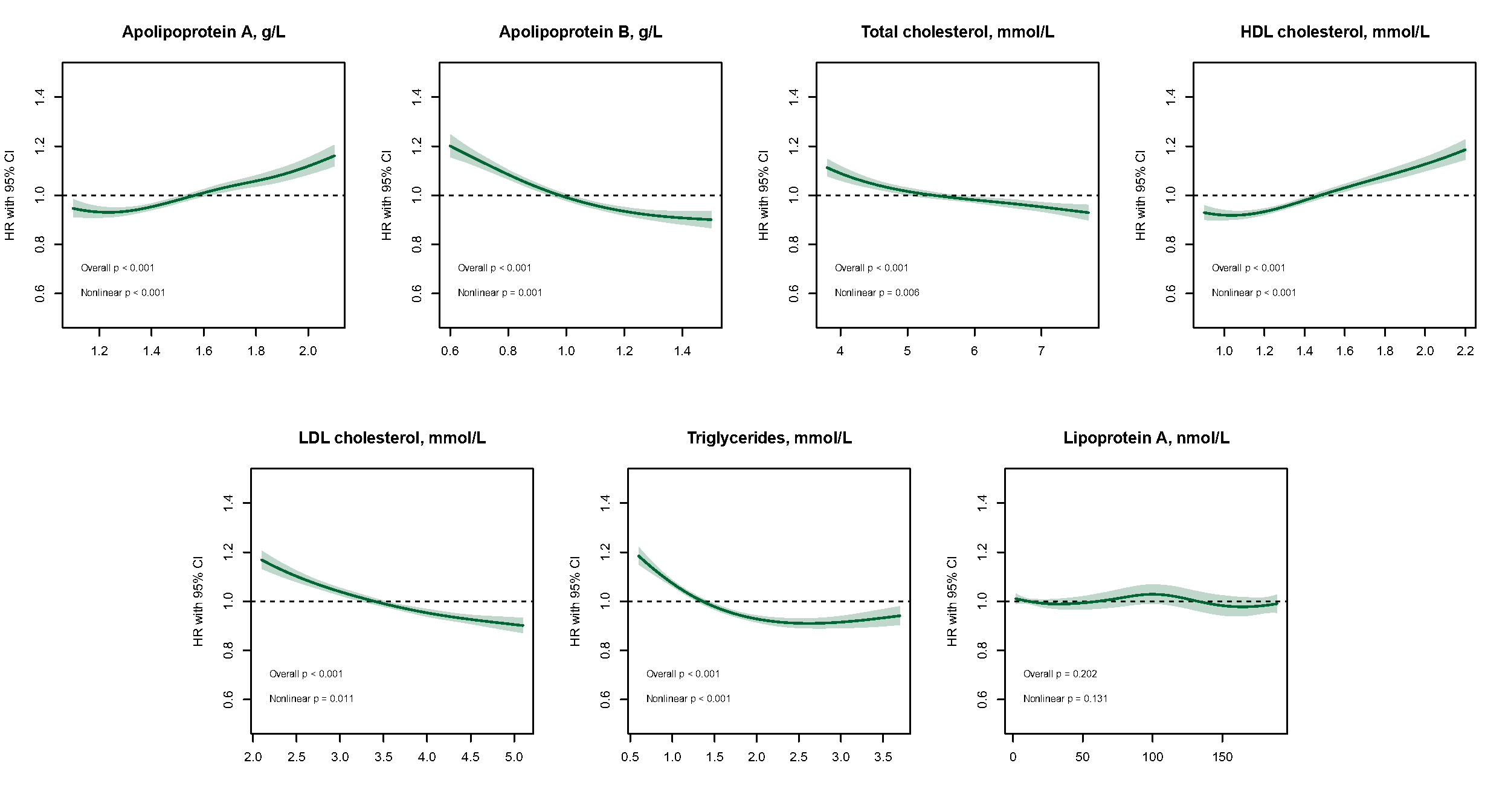


(B)


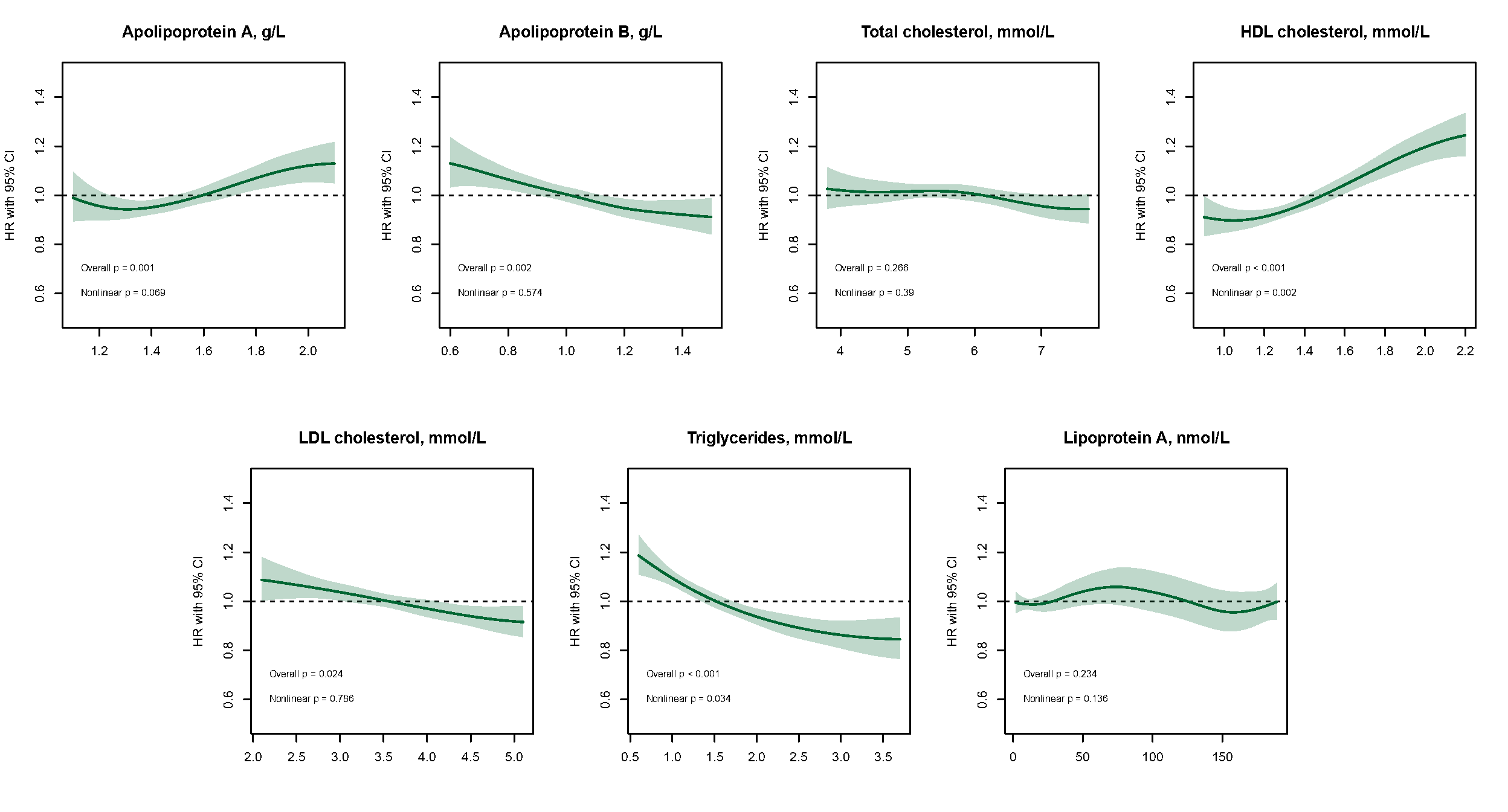


Notes: Model was adjusted for age, sex, ethnicity, deprivation index. Extreme values in the upper and lower 5% of the lipid trait distribution were excluded.

# Supplementary Figure 4. Associations between lipid traits and fracture risk by age, sex, BMI, and cardiovascular disease, and use of lipid-lowering medications.


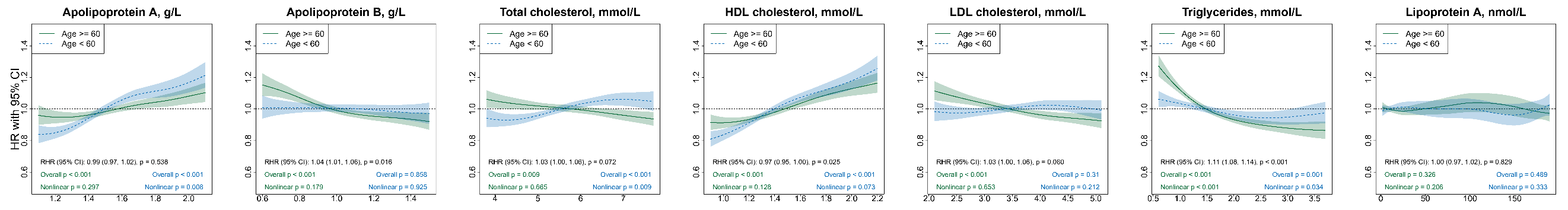


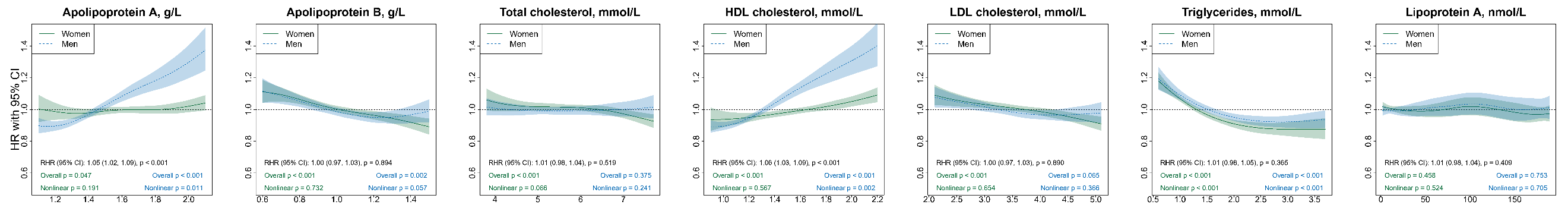


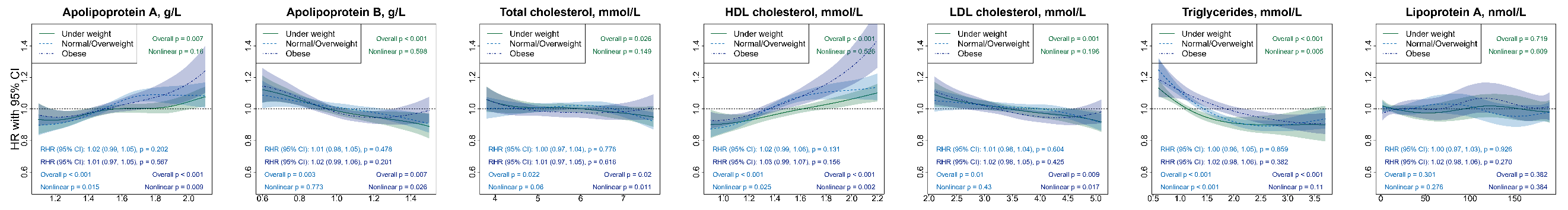


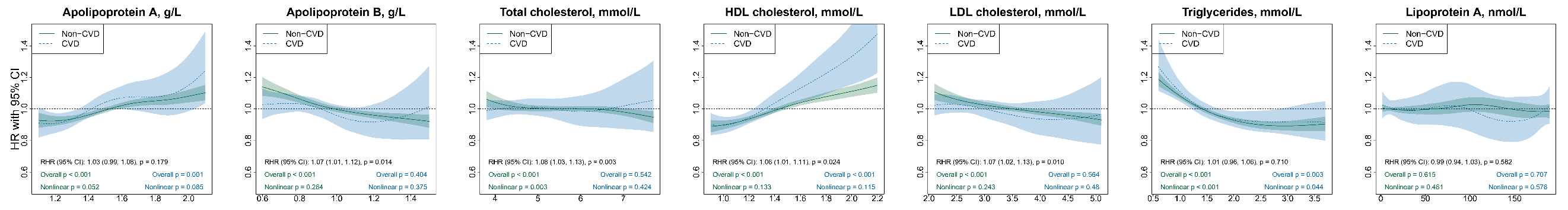


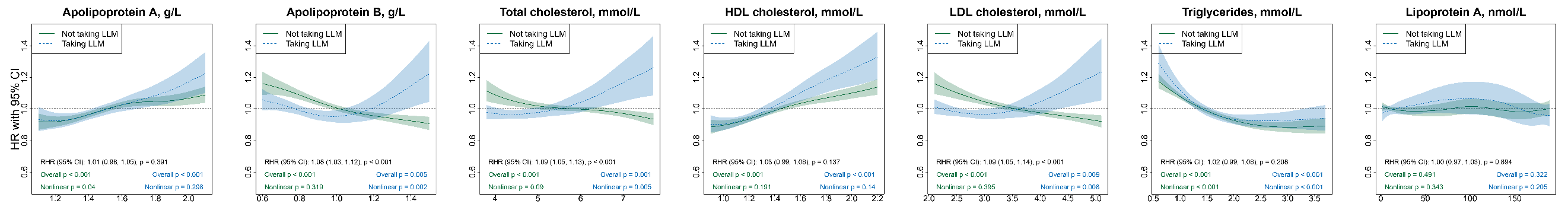


Notes: BMI = Body-mass index; CVD = Cardiovascular disease; LLM = Lipid-lowering medications. The ratio of hazard ratios (RHR) for each risk factor was obtained from the interaction term between each risk factor (as continuous variables) and the potential effect modifier. Model was adjusted for age, sex, ethnicity, deprivation index, current smokers, alcohol intake, sleep duration, total physical activity, total sedentary behavior, fruit and vegetables intake, red meat intake, processed meat intake, oily fish intake, ever eats eggs, ever eats dairy, body-mass index, waist-hip ratio, body fat percentage, number of morbidities, prefrail/frail status, cardiovascular disease, hypertension, diabetes , chronic kidney disease, cancer, anemia, positive rheumatoid factor, vitamin D deficiency, osteoporosis, falls in the last year, menopause status (included in the model for women), lipid-lowering medications, aspirin, glucocorticoids, vitamin D supplements, and calcium supplements. Extreme values in the upper and lower 5% of the lipid trait distribution were excluded.

#
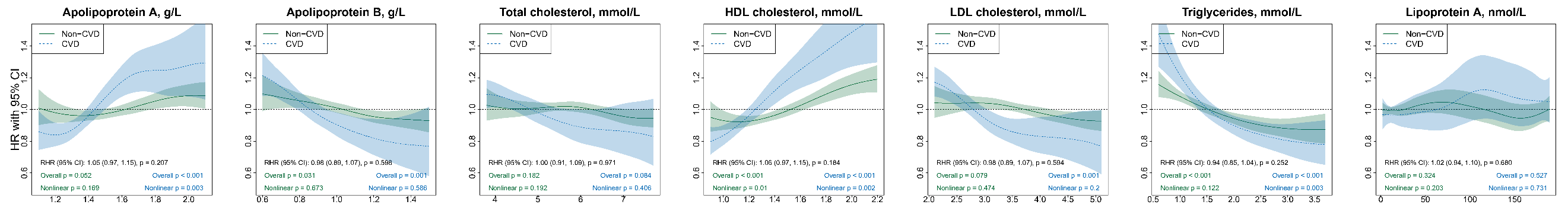

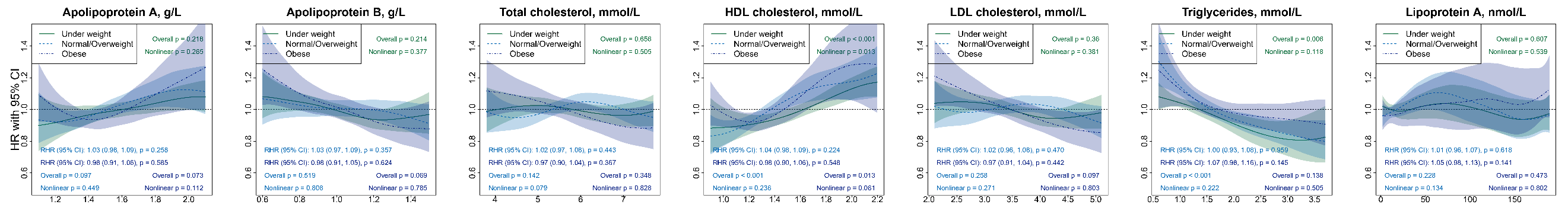

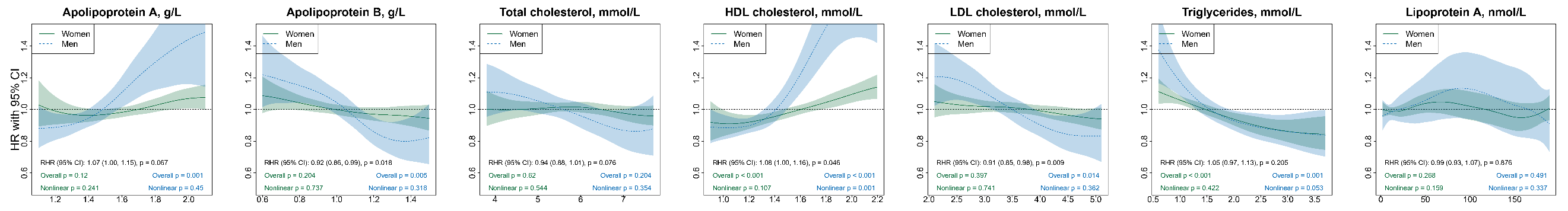

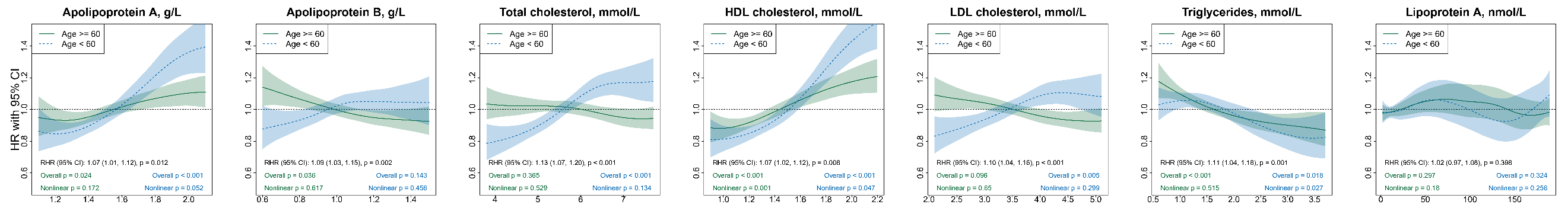
Supplementary Figure 5. Associations between lipid traits and osteoporosis risk by age, sex, BMI, cardiovascular disease, and use of lipid-lowering medications.


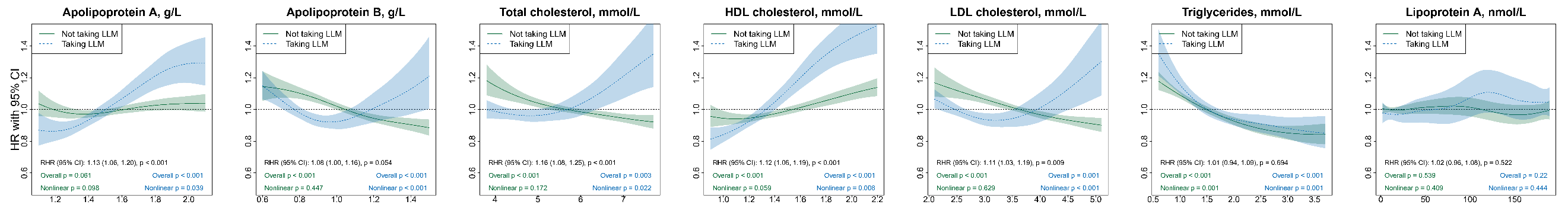


Notes: BMI = Body-mass index; CVD = Cardiovascular disease; LLM = Lipid-lowering medications. The ratio of hazard ratios (RHR) for each risk factor was obtained from the interaction term between each risk factor (as continuous variables) and the potential effect modifier. Model was adjusted for age, sex, ethnicity, deprivation index, current smokers, alcohol intake, sleep duration, total physical activity, total sedentary behavior, fruit and vegetables intake, red meat intake, processed meat intake, oily fish intake, ever eats eggs, ever eats dairy, body-mass index, waist-hip ratio, body fat percentage, number of morbidities, prefrail/frail status, cardiovascular disease, hypertension, diabetes , chronic kidney disease, cancer, anemia, positive rheumatoid factor, vitamin D deficiency, history of fractures, falls in the last year, menopause status (included in the model for women), lipid-lowering medications, aspirin, glucocorticoids, vitamin D supplements, and calcium supplements. Extreme values in the upper and lower 5% of the lipid trait distribution were excluded.
